# Drought Intensity Shapes Soil Legacy Effects on Grassland Plant and Soil Microbial Communities and Their Responses to Future Drought

**DOI:** 10.1111/gcb.70495

**Published:** 2025-09-18

**Authors:** Natalie J. Oram, Nadine Praeg, Richard D. Bardgett, Fiona Brennan, Tancredi Caruso, Paul Illmer, Johannes Ingrisch, Michael Bahn

**Affiliations:** ^1^ Department of Ecology Universität Innsbruck Innsbruck Austria; ^2^ Environment, Soils and Land Use Department, Teagasc Johnstown Castle Ireland; ^3^ Department of Microbiology Universität Innsbruck Innsbruck Austria; ^4^ Department of Earth and Environmental Sciences The University of Manchester Manchester UK; ^5^ Lancaster Environment Centre Lancaster University Lancaster UK; ^6^ School of Biology & Environmental Science University College Dublin Dublin 4, Belfield Ireland; ^7^ Institute for Biodiversity and Ecosystem Dynamics (IBED) University of Amsterdam Amsterdam Netherlands

**Keywords:** climate change, drought resilience, extreme weather, plant–soil interactions, soil microbiome, temperate grassland

## Abstract

Drought can have long‐lasting legacy effects on terrestrial ecosystems via persistent shifts in soil microbial community structure and function. Yet, the role drought intensity plays in the formation of soil‐mediated drought legacies and in determining plant and microbial responses to subsequent droughts is unknown. Here, we evaluate how soil‐mediated drought legacies shaped by the intensity of an initial drought event influence plant and microbial communities in the following year and their response to a subsequent experimental drought. We determined these responses in two model grassland communities with contrasting resource acquisition strategies. We found that the intensity of the initial (i.e., past) drought shaped the composition, network structure and functioning of soil microbial communities, with stronger effects on prokaryotes than fungi. Moreover, drought intensity determined soil‐mediated legacy effects on plant responses to a subsequent drought: increasing past drought intensity decreased the drought resistance of the slow‐strategy plant community and reduced productivity overshoot in the fast‐strategy community after re‐wetting. Our findings demonstrate that increasing drought intensity can lead to distinct legacies in soil microbial community composition and function with impacts on plant responses to future droughts.

## Graphical abstract

Drought can have lasting effects on grasslands above‐ and belowground. We found that drought left soil legacies that persisted into the following year, affecting plant community response to a subsequent drought. These effects depended on the intensity of the initial drought and the plant community resource acquisition strategy. Our findings demonstrate that increasing drought intensity can lead to distinct legacies in soil microbial community composition and function with impacts on plant responses to future droughts.

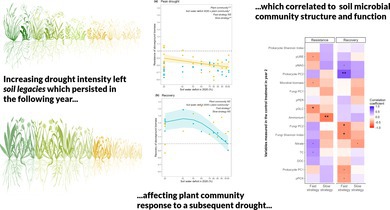

## 1 Introduction

Extreme droughts are becoming more intense and frequent with climate change (IPCC 2023) and threaten to irrevocably change the structure and functioning of terrestrial ecosystems. Recent evidence shows that increasing drought intensity can lead to abrupt changes in plant and soil microbial community composition and functioning, e.g., carbon (C) and nitrogen (N) cycling (Bardgett and Caruso 2020; Cordero et al. 2023; Ingrisch et al. 2023; Oram et al. 2023, 2025). Moreover, apart from initial adverse effects of drought, evidence is mounting that drought causes lasting effects (i.e., legacies) that have consequences for plant and soil microbial communities and modify their responses to subsequent drought (Müller and Bahn 2022; Vilonen et al. 2022; Xi et al. 2022). The intensity of a drought is likely a crucial driver of drought legacy effects on plant and microbial communities, governing the extent to which they recover to their initial state. Differences in drought intensity could also underlie why some previous studies have found drought legacy effects (Canarini et al. 2021; De Long, Jackson, et al. 2019; De Long, Semchenko, et al. 2019), while others have not (De Nijs et al. 2019; Rousk et al. 2013). Thus, explicitly considering drought intensity could improve our ability to predict ecosystem drought response with ongoing increases in drought severity and recurrence. Furthermore, there is mounting evidence that soil‐mediated legacies drive the overall effect of climatic legacies (De Long, Jackson, et al. 2019; De Long, Semchenko, et al. 2019). Here, we determine how increasing drought intensity shapes soil‐mediated drought legacy effects on grassland plant and soil microbial (prokaryote and fungal) communities, soil functioning, and their response to a subsequent drought event.

Soil‐mediated drought legacies manifest as changes in soil microbial community composition and function that persist post‐drought (Canarini et al. 2021; Kaisermann et al. 2017; Leizeaga et al. 2021; Meisner et al. 2018). Previous research has shown that drought leaves legacy effects on soil functioning, decreasing soil multifunctionality (enzymatic activities, soil carbon and nutrient pools and microbial biomass stoichiometry) (Canarini et al. 2021) and reducing soil respiration in the year following drought (Lellei‐Kovács et al. 2025). While drought legacy effects on microbial community composition have been reported to improve microbial community resistance to subsequent drought (Canarini et al. 2021; Leizeaga et al. 2021; Tang et al. 2023) by selecting for microbial drought tolerance traits (Evans and Wallenstein 2014; Ochoa‐Hueso et al. 2018), the legacies of more intense droughts could compromise microbial community resistance and recovery. Increasing drought intensity has been shown to induce a sudden shift in bacterial and fungal community composition, reducing microbial community complexity, leading to impaired functioning, and reducing bacterial regrowth after a subsequent drought (Cordero et al. 2023). These abrupt shifts could alter the strength and direction of the longer‐term drought effects on microbial and plant communities and their responses to subsequent stress. Therefore, a better understanding of how drought intensity shapes microbial and plant responses to subsequent drought stress is crucial to predict grassland functioning with an increasingly extreme climate.

Soil drought legacies are potentially mediated by the plant community's position on the resource economic spectrum, i.e., whether the plant community prioritises defense or fast growth (Grime 1977; Reich 2014). A plant community's strategy could affect drought legacies for two main reasons: differences in the initial microbial community that they foster (Semchenko et al. 2018; Spitzer et al. 2021; Sweeney et al. 2021) and differences in plant drought response (Ingrisch et al. 2018; Oram et al. 2023; Pérez‐Ramos et al. 2013), which likely has knock‐on effects for the microbial community (Fahey et al. 2020; Koyama et al. 2018; Williams and de Vries 2020). For instance, slow‐strategy grassland plant communities are known to harbour soil communities with a higher fungal‐to‐bacterial ratio, compared to fast‐strategy plant communities (de Vries et al. 2012; Orwin et al. 2010). Fungi are generally more drought resistant, with less pronounced shifts in their community composition than bacterial communities in response to drought (Cordero et al. 2023; de Vries et al. 2018; Oram et al. 2025; Preece et al. 2019). Furthermore, slow‐strategy plant communities have generally higher drought resistance than fast‐strategy plant communities, i.e., their productivity is less affected by drought (Ingrisch et al. 2018; Oram et al. 2023; Pérez‐Ramos et al. 2013). What remains unknown is whether fast‐ and slow‐strategy plant communities alter soil drought legacies and whether this is affected by increasing drought intensity.

**FIGURE 1:**
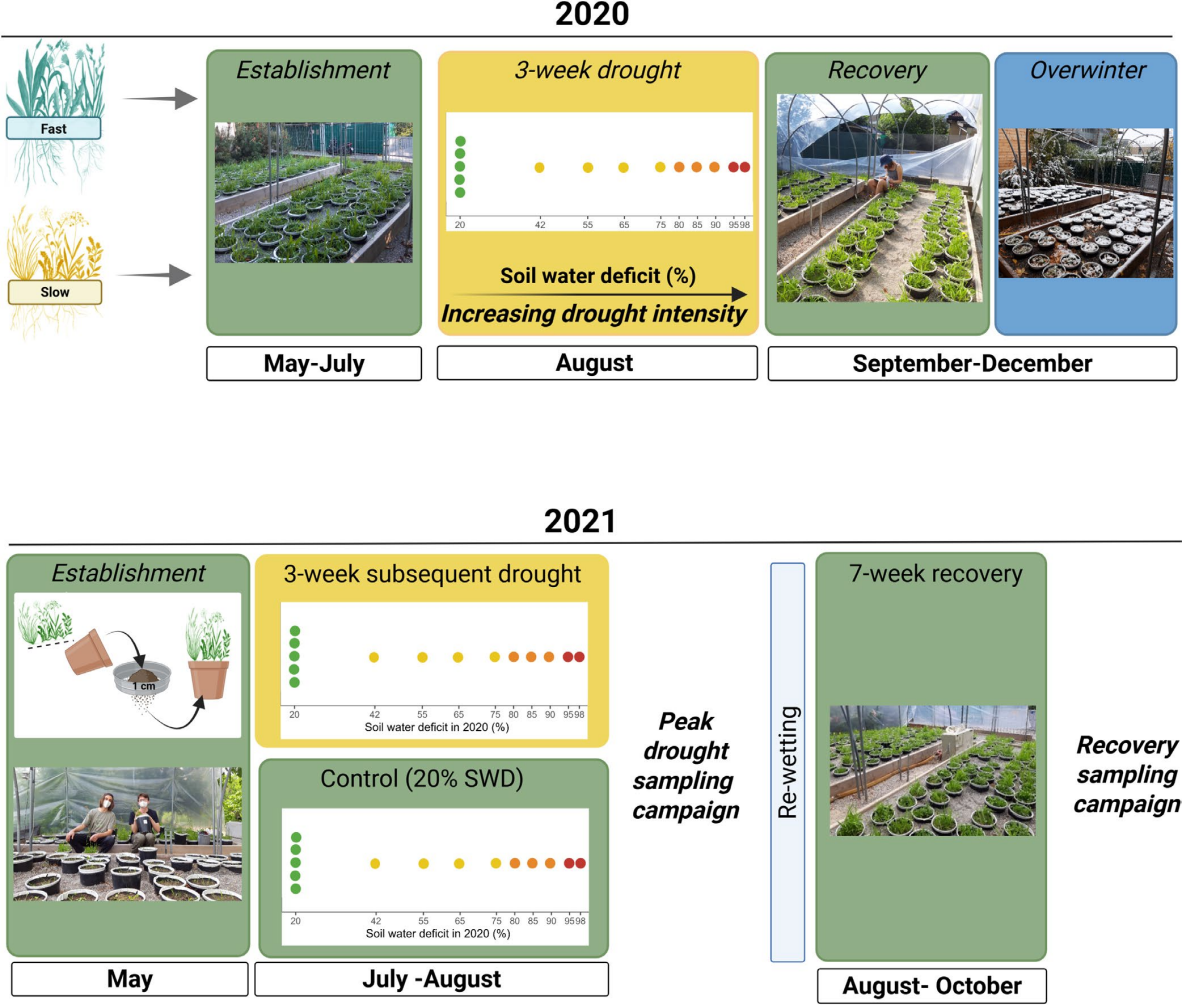
Experimental design. Two model plant communities with contrasting resource acquisition strategies (a fast and a slow strategy) were established in May 2020 (*n* = 112) and exposed to a gradient of increasing drought intensity for 3 weeks in August 2020. The gradient had 10 steps, ranging from well‐watered controls (20% SWD, replicated five times) to communities that were severely droughted (98% SWD). We replicated the control treatment five times per plant community to have a well‐established baseline. The drought intensity treatment followed a gradient design, i.e., without replication per drought intensity level. Top panel: In the first year, we established four identical drought intensity gradients per plant community to enable destructive sampling at two timepoints in the second year. This resulted in 112 experimental units (mesocosms) in total: Each gradient consisted of 5 controls (replicated) and a gradient of 9 levels of increasing drought intensity × fast‐ or slow‐strategy plant communities × drought or control in 2021 × 2 timepoints in 2021 (peak drought and recovery). After the 3‐week drought period in 2020, all communities were re‐wet to 20% SWD and left to recover and overwinter. Bottom panel: In 2021, we isolated the soil‐mediated legacies of increasing drought intensity by removing aboveground biomass and sieving soil to 1 cm to remove most of the root biomass. This legacy soil was then returned to its mesocosm, and new seedlings of the same plant species were transplanted. After establishment, communities were exposed to a subsequent 3‐week drought or maintained at 20% SWD as a control. One set of mesocosms was destructively harvested to determine plant and soil microbial community responses at peak drought, and one set was re‐wet to 20% SWD and harvested after 7 weeks to determine plant and microbial recovery responses.

In an outdoor mesocosm experiment, we determined the soil legacy effects of increasing drought intensity on grassland plant and soil microbial (prokaryote and fungal) communities and their responses to a drought in the subsequent year (Figure 1). Using two model grassland plant communities with contrasting strategies, we investigated whether plant resource acquisition strategy (fast‐strategy versus slow‐strategy) modulates the soil legacy effects of increasing drought intensity. We hypothesized that: (1) increasing drought intensity leaves increasingly pronounced soil legacy effects on the composition and function of the soil prokaryote and fungal community in the subsequent year; (2) increasing drought intensity leaves more pronounced soil legacy effects in fast‐strategy, compared to slow‐strategy plant communities; and (3) soil legacies of increasing drought intensity will decrease microbial and plant community resistance to and recovery from a subsequent drought.

## 2 Materials and Methods

### 2.1 Experimental Design

To test our hypotheses, we set up a 2‐year factorial, fully randomised outdoor mesocosm experiment in the Botanical Garden of the University of Innsbruck, Austria (47°16′04.1″ N 11°22′46.3″ E) with three experimental treatments: soil‐mediated drought legacy (the effect of a drought intensity gradient imposed in the first year, i.e., 2020), drought or control in the second year (i.e., 2021), and plant community strategy (fast or slow resource acquisition strategy).

In the first year, we exposed two model plant communities (a fast‐ and a slow‐strategy community) to a gradient of increasing drought intensity for 3 weeks. The gradient included 10 steps in soil water deficit (SWD) ranging from well‐watered controls (20% SWD) to severely droughted (98% SWD). We replicated only the control treatment to have a well‐defined baseline, while the droughted levels were not replicated and followed a gradient design (Kreyling et al. 2018). Thus, the drought intensity gradient included 14 pots—a control (replicated 5 times) and 9 levels of increasing drought intensity (unreplicated). After exposure to the 3‐week drought intensity gradient (20%–98% SWD) in August 2020, communities were re‐wet to their control weight at 20% SWD and allowed to recover and overwinter. We established four identical gradients for each plant community, which would serve as the legacy soil for the second year of the experiment. Thus, per plant community, gradients would be exposed to either drought or control treatment in year 2 and harvested at either peak drought or recovery (7 weeks after re‐wetting in year 2). In total, the experiment had 112 pots (2 plant communities × 4 drought gradients of 14 pots); see Figure 1, top panel.

In the second year, we harvested the soil from each pot, removed the plants (see below), and transplanted newly germinated plant individuals to isolate soil‐mediated legacy effects of increasing drought intensity from legacies that operate via the plants (e.g., plant physiological or maternal legacies). This allowed us to specifically investigate the soil‐mediated legacy effects of increasing drought intensity on plant and soil microbial responses to a subsequent drought (Figure 1, bottom panel).

### 2.2 Pre‐Experiment: Establishing the Soil Drought Legacy Treatment (Year 1)

In 2020, we grew two model plant communities (a fast and a slow resource acquisition strategy plant community) in 7 L mesocosms (21 cm Ø, 25 cm height); see Oram et al. (2023) for a complete description of the methods. Perennial plant species common to European mesotrophic grasslands were selected based on their traits related to their resource acquisition strategy: the fast‐strategy community included species with high specific leaf area, high leaf nitrogen concentration and low leaf dry matter content, and the slow‐strategy community included species with opposite trait values (Reich 2014). Communities were assembled based on a priori trait values reported in the literature (Baxendale et al. 2014; De Long, Jackson, et al. 2019; De Vries and Bardgett 2016) and confirmed in August 2020 (Oram et al. 2023). The fast‐strategy community included the grasses *Lolium perenne, Dactylis glomerata, Phleum pratense* and the forbs: *Plantago lanceolata* , *Leontodon hispidus* , *Rumex acetosa* . The slow‐strategy community included the grasses: *Anthoxanthum odoratum, Briza media, Festuca rubra* and the forbs *Leucanthemum vulgare, Campanula rotundifolia* and *Prunella vulgaris* .

Seedlings were germinated in field soil, and then in May 2020, two individuals per species (12 individuals in total per mesocosm) were transplanted into the mesocosms, which were filled with field soil provided by the Botanical Garden, University of Innsbruck. The soil was classified as sandy loam: 53.5% sand (50–2000 μm), 35.6% silt (2–50 μm) and 10.8% clay (< 2.0 μm). Initial chemical properties were as follows: 7.57% organic matter (loss on ignition method, 550°C), 0.29% total N, 1.10 g kg^−1^ plant available P, 3.58 g kg^−1^ plant available kg^−1^ K, and a pH_CaCl2_ of 7.67. See Oram et al. (2023) for a description of the methods. Prior to filling the mesocosms, the soil was sieved to 1 cm to homogenise and remove large stones. The soil water content was determined at field capacity (g_water_ g_fresh soil_^−1^) and the mesocosm weight at 80% field capacity (i.e., 20% SWD) was recorded.

The soil drought legacy treatment was established by exposing the communities to a gradient of increasing SWD over a period of 3 weeks (July 21st–August 13th, 2020), Figure S1. The SWD gradient ranged from 20% (well‐watered, corresponding to a soil moisture of 0.266 g_water_ g_fresh soil_^−1^) to 98% (severely droughted, 0.020 g_water_ g_fresh soil_^−1^). The SWD gradient was maintained by weighing the mesocosms 5 to 7 times per week, and watering to weight. On August 14, 2020, all mesocosms were re‐wet to their weight at 20% SWD over 3 days, which was maintained until the end of the growing season. Mesocosms remained outside over the winter period. The effects of increasing drought intensity on plant and microbial communities and plant–soil C cycling in 2020 (year 1) are reported in (Oram et al. 2023, 2025) and are not discussed further in this paper.

### 2.3 Focal Experiment: Determining Soil Legacy Effects of Increasing Drought Intensity on Plant and Microbial Response to Subsequent Drought

In 2021, we determined how the soil legacies of increasing drought intensity in 2020 (year 1) affected plant and microbial response to a subsequent drought, compared to control conditions (i.e., in year 2, 2021), in plant communities with a fast or slow resource acquisition strategy. Climatic conditions throughout the experiment in 2021 are reported in Figure S2.

#### 2.3.1 Soil Preparation

We isolated the soil legacy effects of increasing drought intensity by removing the plant's above‐ and belowground biomass from the soil. Aboveground biomass that had grown since October 13, 2020, was harvested on May 10, 2021, sorted per species, dried at 60°C for 3 days and then weighed. Belowground (root) biomass was removed by sieving the fresh soil with a 1 cm sieve. To minimise the effects of sieving on soil aggregates, we chose not to use a smaller sieve, and thus, a small fraction of fine roots remained in the soil. Roots were collected, washed over a 0.5 mm sieve, dried at 60°C for 7 days and weighed. Sieves and the workspace were washed and disinfected with 70% ethanol between each mesocosm. The moisture content of the legacy soil was determined, and the legacy soil from each mesocosm was stored in plastic bags in a cool room (10°C) for 2 days. Because some legacy soil was lost during sieving, we first added 0.78 kg (dry weight equivalent) of the non‐legacy field soil (i.e., the soil that we initially used to fill the mesocosms in 2020) that was steamed at 90°C for 6 h. We then added 5.0 kg (dry weight equivalent) of fresh legacy soil on top. We stacked the soils rather than mixing them to avoid diluting the legacy soil. In this way, the mesocosms were properly filled and the plants would first have contact with the legacy soil only. Mixing ‘conditioned’ soil with steamed or sterilised soil is a common practice in plant–soil feedback and climate‐feedback experiments, generally adding less conditioned soil (5%–30%) than sterilised soil (Crawford and Hawkes 2020; Pernilla Brinkman et al. 2010; Spitzer et al. 2022; Xi et al. 2022). Thus, we are confident that by adding a much larger amount of conditioned soil than steamed soil, we did not compromise the biotic soil drought legacies. Soil moisture was adjusted to 80% of field capacity (20% SWD), and the weight recorded.

#### 2.3.2 Plant Communities

Seeds were surface sterilised with a 1:1 household bleach: tap water for 20 min and then rinsed thoroughly with tap water before germinating in field soil (the same soil that was used in the mesocosms in 2020). Two‐week‐old seedlings were transplanted into the mesocosms on May 17–18, 2021. Due to poor *B. media* germination, the number of species was reduced to two forbs and two grasses per community: *L. perenne, D* *. glomerata* (grasses) and *P. lanceolata, L. hispidus* (forbs) in the fast‐strategy community, and *A. odoratum, F* *. rubra* (grasses) and *L. vulgare, P* *. vulgaris* (forbs) in the slow‐strategy community. These species were dominant in terms of aboveground biomass in 2020 (Oram et al. 2023). Three individuals per species were used so the density remained 12 individuals per mesocosm (346 individuals/m^2^). Seeds were commercially sourced: *D. glomerata* , *F. rubra* and *L. perenne* from Barenbrug BV, the Netherlands and *A. odoratum* , *L. vulgare* , *P. lanceolata, L* *. hispidus* and *P. vulgaris* from Jelitto, Germany.

#### 2.3.3 Drought and Control Treatments

To experimentally induce a drought, we installed a rainout shelter over all mesocosms from July 13, 2021, to August 4, 2021. The rainout shelter was made from an aluminium frame 2.5 m high covered with light‐ and UV‐B‐permeable plastic (Lumisol clear AF, Folitec, Westerburg, Germany, light transmittance *c*. 90%). Control mesocosms were maintained at 20% SWD by watering to weight approximately 5 times per week (Figure S3). We destructively harvested half of the mesocosms on August 4, 2021, to determine soil responses to drought at peak drought. The other half of the mesocosms was kept intact to monitor recovery responses. The droughted mesocosms in this set were re‐wet to 20% SWD over 3 days (August 4–August 6, 2021).

#### 2.3.4 Above‐ and Belowground Biomass

Species‐specific aboveground biomass was harvested from all mesocosms on July 6th, 2021 (*n* = 112), at peak drought (August 3–4, 2021, *n* = 112) and at recovery (7 weeks after re‐wetting, September 22nd, 2021, *n* = 56). At each harvest, we cut aboveground biomass to 3 cm above the soil surface, dried it at 60°C for 4 days, and weighed it. These harvests were needed to standardise the aboveground biomass before the drought treatment and to quantify the drought/control treatment effects on plant biomass during drought and recovery. While cutting could have altered rhizodeposition immediately before the drought treatment started (Rajper et al. 2024; Xu et al. 2025), potentially masking some of the soil drought legacy effects, it should have also reduced differences in plant‐biomass‐related rhizodeposition effects in all mesocosms at the onset of the year 2 drought treatment. We determined belowground biomass at peak drought and recovery by washing the soil from the roots in the mesocosms using a 0.5 mm sieve, drying it at 60°C for at least 5 days and weighing it. Plant community resistance and recovery were determined by dividing community aboveground biomass in the year 2 drought treatment by the aboveground biomass in the year 2 control treatment growing in soil that had been maintained at 20% SWD in year 1. Thus, our continuous baseline (*sensu* Ingrisch and Bahn 2018) was the plant communities growing in soil that were in the control treatment in 2020 and that were in the control treatment in 2021.

#### 2.3.5 Soil Sampling

Soil was sampled directly prior to harvesting soil to set up the experiment to test soil legacies in the spring (April 30, 2021, *n* = 28), at peak drought (August 3, 2021, *n* = 56) and at recovery (September 23, 2021, *n* = 56). At the spring sampling, we took two soil cores (2 cm Ø, 25 cm depth, i.e., to the bottom of the mesocosm) to conserve soil for the experimental setup; at the peak drought and recovery campaigns, we took five soil cores. For all samplings, soil from the cores was pooled per mesocosm and sieved to 2 mm. A sub‐sample of soil for total soluble nitrogen (NO_3_ + NO_2_ and NH_4_), dissolved organic carbon (DOC) and dissolved organic nitrogen (DON), potential extracellular enzyme activity (pEEA) and basal and substrate‐induced respiration analysis was stored in coolers with icepacks on the sampling day and then at 4°C until analysis, which took place within the following 7 days. A sub‐sample of soil for amplicon sequencing of the microbial community was frozen on dry ice and then stored at −80°C until analysis. Sieves and soil cores were washed with water, dried and then disinfected with 70% ethanol between each mesocosm.

#### 2.3.6 Soil Functioning

We measured potential hydrolytic and oxidative enzymatic activities and soluble C and N fractions in fresh soil as indications of soil C and N cycling capacity. Potential activity of β‐glucosidase (pGLC) and β‐N‐acetylglucosaminidase (pNAG) was measured photometrically using *p*NP‐linked substrates (Jackson et al. 2013). Urease (pURE) was analysed by measuring ammonium production after urea addition to the soil (Cordero et al. 2019). Peroxidase (pPER) and phenoloxidase (pPOX) activities were measured by photometrically determining the oxidation of L‐3,4‐dihydroxyphenylalanin (_L_‐DOPA) (Sinsabaugh and Linkins 1988). See Supporting Information methods for a full description.

Soluble N and C fractions were determined by shaking 7.5 g of fresh soil in 30 mL of 1 M KCl for 1 h at 300 rpm on a horizontal shaker (IKA KS 260 basic). Soil suspensions were centrifuged (1218 × g for 10 min) and filtered (Whatman 1 filter paper, pre‐washed with 10 mL 1 M KCl). Extracts were stored at 4°C until analysis, which took place within 48 h. NO_3_ + NO_2_ and NH_4_ were measured on a San^++^ Continuous‐Flow Analyser (Skalar, Netherlands). DOC and DON were measured with a TOC‐L/TNM‐L (Shimadzu, Japan).

Substrate‐induced respiration was determined on an EGA61‐Soil respiration device (ADC BioScientific, UK). Fresh soil (40 g dry weight equivalent) was filled into acrylic glass tubes, closed with polystyrene foam pads, and aerated with a continuous stream of ambient air (humidified and tempered to 22°C). The CO_2_ released from the samples was recorded for 16 h to calculate the basal soil respiration (μg CO_2_ g^−1^ dw h^−1^). Subsequently, glucose (1.5%, w/w dry weight) was added to the samples, and the CO_2_ release was recorded for a further 8 h to determine substrate‐induced respiration. The maximum CO_2_ release was used to calculate the microbial biomass (μg C g^−1^ dw) (Anderson and Domsch 1978).

#### 2.3.7 Microbial Communities

DNA was extracted from fresh‐frozen soil using the DNeasy PowerSoil Pro Kit (Qiagen, Germany), and then the 16S rRNA (prokaryotes) and ITS (fungi) marker genes were sequenced on Illumina NextSeq and MiSeq platforms, respectively. Prokaryote community composition was determined by sequencing the V4–V5 region of the 16S rRNA marker gene with primer pair 515F and 926R (Walters et al. 2016). We targeted ITS2 to determine fungal community composition using primer pair ITS86F–ITS4 (Vancov and Keen 2009). For complete details, see Supporting Information Methods.

We used DADA2 to filter, trim, merge paired‐end reads, remove chimaeras and construct amplicon sequence variants (ASVs) (Callahan et al. 2016). Taxonomy was assigned using the SILVA 138.1 taxonomy database for prokaryotes (Quast et al. 2012) and the UNITE version 9.0 taxonomy database for fungi (Nilsson et al. 2019). Rarefaction curves indicated that there was adequate coverage and sequencing depth (Figure S4). Raw sequencing data were deposited in the NCBI Sequence Read Archive (SRA) and are accessible under the BioProject ID (available upon publication).

### 2.4 Data Analysis

Statistical analysis was carried out in R version 4.3.0 and higher (R Core Team 2024). Data exploration was carried out in tidyverse (Wickham and RStudio 2023), and figures were made with ggplot (Wickham et al. 2023) and cowplot (Wilke 2020). To facilitate analysis that required categorical explanatory variables, we added the *soil legacy group* factor by dividing the 2020 SWD gradient into three levels: control (20% SWD in 2020, *n* = 5 mesocosms per plant community per timepoint), mild drought (40%–75% SWD, *n* = 4 mesocosms per plant community per timepoint) and severe drought (80%–98% SWD, *n* = 5 mesocosms per plant community per timepoint). We chose the cutoff at 76% SWD because this is where the thresholds in plant community aboveground biomass (productivity) occurred in 2020 (Oram et al. 2023). Including the 80% SWD level in the mild drought legacy group did not affect the outcome of any of the statistics.

#### 2.4.1 Soil Microbial Community α‐ and β‐Diversity

Prokaryote and fungal α‐diversity of each community was estimated using the function estimate_richness from phyloseq version 1.16.2 (McMurdie and Holmes 2013). The effects of soil legacy group (control, mild drought, or severe drought in 2020) or soil legacies of increasing drought intensity (i.e., the drought intensity gradient in year 1/SWD in 2020), drought or control in 2021/year 2, plant community (fast or slow resource acquisition strategy) and all 2‐way interactions on prokaryote and fungal α‐diversity (Shannon diversity) with linear mixed effects models using the function lme from the R‐package nlme (Pinheiro et al. 2023). Models were simplified by removing non‐significant interactions, and the model with the lowest Akaike Information Criterion (AIC) value was retained. Models were checked for residual normality and homoscedasticity using the function check_model() from the package performance (Lüdecke et al. 2023).

The effects of the soil legacy group (drought or control in year 2), plant community resource acquisition strategy and all 2‐way interactions on the prokaryote and fungal β‐diversity were determined with PERMANOVA using the adonis2() function from the R‐package vegan (Oksanen et al. 2022). Data were first centred log ratio (CLR‐transformed), and Euclidean distances were used in PERMANOVA (thus, Atchinson distances were used to establish differences between communities). We determined differences between levels of the soil legacy group factor using pairwise multilevel comparisons with the function pairwise.adonis from the package pairwiseAdonis (Martinez Arbizu 2020). Redundancy analysis (constrained ordination) was carried out on CLR transformed data using the function rda(), from the R‐package vegan (Oksanen et al. 2022) to determine the effect of plant and soil variables on fungal or prokaryote community composition. We first included the following variables: SWD 2020, aboveground biomass, belowground biomass, N‐NO_3_^−^, N‐NH_4_^+^, DOC, DON and microbial biomass. We then used the function ordi2step from the R‐package vegan to determine the final model using forward variable selection based on model adjusted‐*R* ^2^. Models were tested for global and term significance using the function anova.cca() and collinearity of terms with vif.cca(), both from the R‐package vegan.

The effects of soil legacies of increasing drought intensity (the drought intensity gradient in year 1/SWD 2020), drought or control in year 2, plant community resource acquisition strategy and all 2‐way interactions on the relative abundance of microbial phyla and families were determined in linear models using the function lm. Models residuals were visualised and checked for normality and homogeneity of variance using the functions check_model from the package performance (Lüdecke et al. 2023), Levene's test using the function leveneTest from package car (Fox et al. 2023), and Anderson‐Darling test (ad.test) from package nortest (Gross and Ligges 2015). In line with Cordero et al. (2023), *p* values were adjusted with the Benjamini‐Hochberg adjustment (Benjamini and Hochberg 1995) using the R function *p*.adjust.

#### 2.4.2 Network Analysis

We used co‐occurrence network analysis to determine structural changes in the prokaryote and fungal community in response to the past drought intensity. Data were first filtered to exclude ASVs with a relative abundance of < 0.00025% and ASVs that were present in < 5 experimental units. Co‐occurrence networks were constructed per soil legacy group (control, mild drought, or severe drought in 2020), timepoint (peak drought or recovery in year 2) and kingdom (prokaryote or fungal) using CLR‐transformed read counts. To infer co‐occurrence patterns, we used an inference method to estimate the conditional independence between any two nodes, rather than using traditional correlational approaches, which overestimate direct links via the implicit effect of indirect correlations (Kurtz et al. 2015). We estimated conditional independence using Meinshausen and Buhlmann's neighbourhood estimation method, which we implemented with the function spiec.easi from the R‐package SpiecEasi (Kurtz et al. 2015). Optimal stability parameters were selected using the StARS selection approach. The validity of each co‐occurrence network was checked with the function getStability from the package SpiecEasi. Networks were visualised using the adj2igraph function from igraph (Csárdi et al. 2024).

We derived three network properties that capture network complexity (transitivity, modularity and within:between link ratio) and one property that captures network stability (robustness). Transitivity, calculated using the function transitivity() from igraph (Csárdi et al. 2024), is a clustering coefficient that measures the probability that adjacent nodes are connected. Modularity, calculated using the function cluster_fast_greedy() from igraph (Csárdi et al. 2024), identifies the presence of dense subgraphs (modules) and is a measure of how compartmentalised a network is. High modularity indicates that nodes are more connected within than between sub‐groups, also known as network community structure. In ecology, networks with high modularity have been found to be more stable because perturbations to individual nodes tend to be confined to the main community or module to which the node belongs, thereby not propagating throughout the network (Stouffer and Bascompte 2011; Yuan et al. 2021). The within: between link ratio calculates the ratio between connections between nodes within a module and connections between nodes in other parts of the network. We calculated robustness as a measure of network stability using the function ‘robustness’ from the R‐package brainGraph (Watson 2020). Robustness is the ability of a network to withstand targeted removal of nodes (i.e., its ability to withstand ‘disturbance’) (Barabási 2016). The function performs a targeted attack on the network and calculates the size of the largest component after node removal. We calculated the area under the size ~ node removal robustness curve as our indication of robustness, where a larger area indicates a more robust (i.e., a more stable) network.

Network patterns may simply emerge from the stochastic associations between nodes (i.e., the randomness or chance that nodes co‐occur). Therefore, we assessed the structural, non‐stochastic role of node identity on the observed network patterns by generating a set of random networks based on fluctuating constraints using a maximum entropy approach (Caruso et al. 2022). We then determined if our observed networks were significantly different from random by comparing them with a random network ensemble (999 random network models derived using a canonical, undirected, binary configuration model), Caruso et al. (2022). Network properties were extracted from each of the 999 random networks, and a *z*‐score was calculated to determine the direction and magnitude of divergence between the observed network and the null model distribution of the random network ensemble (Caruso et al. 2022). For approximately normally distributed metrics, a *z*‐score above or below two or more SD of the mean would imply that the observed network metrics have a probability of being observed in the ensemble below 0.05. In other words, a significant *z*‐score would imply that the degree sequence alone is not sufficient to explain network patterns and that node identity in our observed networks matters to network structure.

#### 2.4.3 Soil Microbial Community Functioning and Soil Physicochemical Properties

We used a constrained ordination of pEEA (pGLC, pNAG, pPOX, pPER, pURE) and soluble soil nitrogen (NO_3_^−^ and NH_4_^+^) as a proxy for soil microbial community functioning, using the function rda() from the R‐package vegan on scaled data (mean ± 1). The effects of soil legacy group (control, mild drought or severe drought in 2020), drought or control in year 2, and plant community (fast or slow resource acquisition strategy) and all 2‐way interactions on multivariate ‘soil functioning’ were determined with PERMANOVA using the function adonis2() from the R‐package vegan (Oksanen et al. 2022). The effect of continuous variables (SWD in 2020 and measured response variables) was determined with the function anova.cca() from the R‐package vegan (Oksanen et al. 2022). As above, we began with the same set of variables for the peak drought and recovery timepoints in year 2: SWD 2020, belowground biomass, aboveground biomass, microbial biomass, DOC, prokaryote and fungal community composition (PCA scores, Figure S12), and prokaryote and fungal α‐diversity (Shannon Index). For the spring timepoint (i.e., before the drought treatment in year 2) we began with the variables listed above, excluding the prokaryote and fungal community α‐ and β‐diversity as we did not carry out amplicon sequencing at this timepoint. We simplified the models using stepwise forward selection with the function ordi2step from the package vegan based on adjusted *R* ^2^. Explanatory variables were checked for collinearity with the function vif.cca() from the R‐package vegan.

The effects of soil legacy group, drought, or control in year 2, plant community resource acquisition strategy, and all 2‐way interactions on the pEEA of individual enzymes and soil physicochemical properties (soluble N and C) were tested with linear mixed effects models using the function lme (Pinheiro et al. 2023). Each model was simplified by stepwise removal of non‐significant interactions, and the model with the lowest AIC value was retained. Model fit was checked with check.model() from the R‐package performance (Lüdecke et al. 2023). Model significance was tested with ANOVA, and post hoc tests on significant factors were carried out using the emmeans() function from the R‐package emmeans (Lenth et al. 2023).

#### 2.4.4 Plant Community Resistance and Recovery

The effect of soil legacies of increasing drought intensity (drought intensity in year 1) on the resistance to and recovery of plant community productivity (community aboveground biomass) from the drought in year 2 was determined with generalised additive models (GAM) using the function gam () from the R package mgcv (Pedersen et al. 2019). We modelled the following relation:

$$Y=\beta_{0}+\beta_{1}\left(\text{plant community}\right)+\beta_{1}\left(\mathrm{SWD}\;2020\right)+f_{1}\left(\mathrm{SWD},\text{plant community}\right)$$

where $\beta_{0}$ represents the intercept, $\beta_{1\left(\text{plant community}\right)}$ the effect of the plant community resource acquisition strategy (fast or slow), $\beta_{1\left(\mathrm{SWD}\;2020\right)}$ the overall effect of the soil water deficit in 2020, and $f_{1}\left(\mathrm{SWD}\;2020,\text{plant community}\right)$ the interactive effect between the soil water deficit in 2020 and plant community resource acquisition strategy. When a significant interaction was detected, we determined the effect of the soil water deficit in 2020 within each plant community resource acquisition strategy by modelling the relation (where plant community is considered an ordered factor: fast‐ or slow‐strategy):

$$Y=\beta_{0}+\beta_{1}\left(\text{plant community}\right)+f_{1}\left(\mathrm{SWD},\text{plant community}\right)$$

Model fit was checked using gam.check() from the R‐package mcgv (Pedersen et al. 2019). Models with different smoothers and families were compared using AIC, and the best fit model (lowest AIC) was retained.

We determined how the soil legacy effects of increasing drought intensity (SWD in 2020) influenced plant community resistance and recovery to the drought in year 2 with rank (Spearman) correlations. We related the soil variables that we measured in the control treatment at the peak drought and recovery timepoints in year 2 to plant community drought resistance and recovery (respectively). This allowed us to isolate the influence of soil biotic and abiotic legacies of increasing drought intensity on the productivity response of a plant community to drought. We used complete‐linkage hierarchical clustering on the values of the correlations to cluster variables in the heatmap to visualise which measured variables had similar relationships with plant community resistance and recovery.

## 3 Results

### 3.1 Soil Legacies of Increasing Drought Intensity Affect Prokaryote and Fungal Community Composition

**FIGURE 2:**
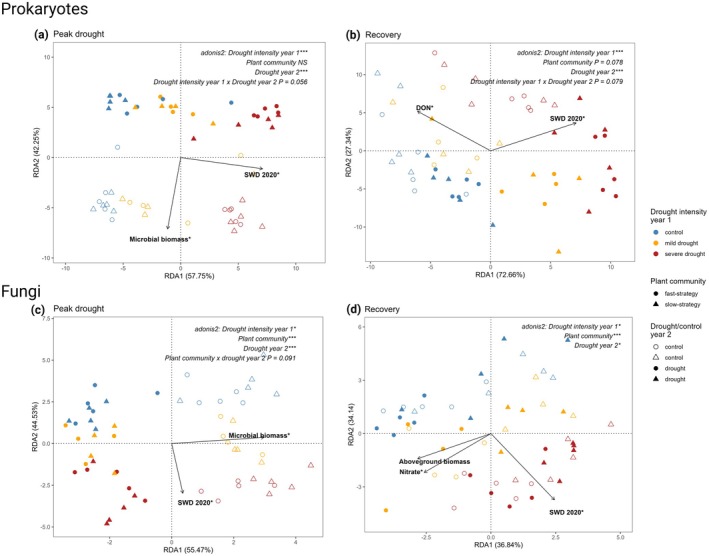
Prokaryote and fungal community compositional responses to soil legacies of increasing drought intensity (soil water deficit in year 1, SWD 2020), drought or control in year 2, and plant community resource acquisition strategy (fast or slow) at peak drought in year 2 (a, c) and 7 weeks after re‐wetting (recovery, b, d). The effects of soil legacy group (control, mild drought, or severe drought in year 1), drought or control in year 2, plant community resource acquisition strategy (fast or slow strategy), and all interactions were determined with PERMANOVA using adonis2 (Table S1). Significance of measured variables (arrows) was tested with anova.cca (*p* < 0.05*, *p* < 0.001***) and includes SWD 2020, aboveground biomass, soil nitrate (nitrate), dissolved organic nitrogen (DON) and microbial biomass (see Table S2 for statistical output).

We found that soil legacies of drought in year 1 significantly affected soil prokaryote and fungal community composition throughout year 2, became more pronounced with increasing past drought intensity (i.e., with increasing SWD in 2020), and were stronger in the prokaryote than in the fungal community (Figure 2a–d, PERMANOVA results are reported in Table S1, anova.cca results for the RDA are reported in Table S2). Prokaryote and fungal α‐diversity (Shannon index) in year 2 was not affected by soil legacies of increasing drought intensity in year 2 (Figure S5, Table S3).

In line with shifts in prokaryote community composition (Figure 2a,b), we found that soil legacies of increasing drought intensity affected the relative abundance of prokaryote phyla and families in year 2 (Figure S6, Table S4). Throughout the experiment, communities exposed to a severe drought in year 1 and no drought in year 2 (i.e., the control treatment in year 2) had significantly higher relative abundance of Crenarchaeota and lower relative abundance of Proteobacteria (Figure S6a,b). The relative abundance of the ammonium‐oxidising archaea (AOA) Nitrososphaeraceae was significantly higher in soil that had experienced a severe drought in year 1, while the relative abundance of the ammonium‐oxidising bacteria (AOB) Nitrosomonadaceae was significantly lower (Figure S6e,f, Table S4). At the recovery timepoint in year 2 (7 weeks after re‐wetting), the relative abundance of Actinobacteriota increased and Acidobacteriota decreased in communities that had experienced a severe drought in year 1, compared to the control in year 1 (Figure S6b).

Regardless of exposure to the drought in year 2, the relative abundance of fungal phyla or families was not affected by soil legacies of increasing drought intensity (Figure S6c,d,g,h, respectively). Thus, while fungal community composition was affected by soil legacies of increasing drought intensity (Figure 2c,d), there were no significant differences in relative abundance at the phyla or family levels between the control, mild drought, or severe drought treatment in year 1 (Figure S6c,d,g,h, respectively).

### 3.2 The Drought in Year 2 Affected Prokaryote and Fungal Communities Regardless of Soil Legacies of Increasing Drought Intensity

The drought in year 2 significantly affected prokaryote and fungal community composition at peak drought and recovery (Figure 2a–d). At the peak drought timepoint in year 2, we found that the year 2 drought treatment significantly interacted with soil legacies of increasing drought intensity (control, mild drought or severe drought in year 1), adonis2: *p* = 0.046, Figure 2a. Despite the significant interaction effect, we found that the year 2 drought significantly shifted prokaryote community composition regardless of the intensity of the previous drought, with only small differences in effect size (Table S1). Based on RDA analysis, the effect of the drought in year 2 on prokaryote community composition was associated with a decrease in soil microbial biomass (Figure 2a, Table S2). The year 2 drought significantly affected the relative abundance of most of the dominant prokaryote families (Figure S6e). Similar to the effects of the soil drought legacies, the drought in year 2 decreased the relative abundance of Nitrosomonadaceae (Figure S6e). The responses of prokaryote family relative abundance to the drought in year 2 were not affected by soil legacies of increasing drought intensity (i.e., there were no significant interaction effects; Figure S6, Table S4).

At the recovery timepoint in year 2 (7 weeks after re‐wetting), prokaryote community composition significantly differed between communities that had experienced the year 2 drought and those maintained at control soil moisture (Figure 2b, Table S2). This was related to lower dissolved organic nitrogen (DON) in the droughted communities, compared to the control (Figure 2b, Table S2). There was no significant effect of soil legacies of increasing drought intensity on the recovery response of the prokaryote community in year 2 (i.e., no significant interactive effect between soil drought legacy and drought in year 2). Soil legacies of increasing drought intensity significantly affected the recovery response of Actinobacteriota, in terms of relative abundance, from the year 2 drought (Figure S6b, Table S4). The relative abundance of Actinobacteriota significantly increased following the year 2 drought if the community had experienced a mild or severe drought in year 1, while its relative abundance remained the same if the community was a control in year 1 (Tukey posthoc test: *p* < 0.001 for drought vs. control year 2 in communities with a mild or severe drought in year 1, *p* = 0.4699 for drought vs. control year 2 in communities with a control in year 1). The year 2 drought significantly affected the relative abundance of most of the dominant prokaryote families at recovery (Figure S6f). At the recovery timepoint, we found that prokaryote α‐diversity (Shannon index) was significantly higher in communities exposed to the year 2 drought and re‐wetting event compared to those maintained at control conditions (Figure S5).

Fungal community composition significantly differed between the year 2 drought and control treatments at peak drought and recovery (Figure 2c,d, respectively; Table S2). Neither plant community resource acquisition strategy nor soil drought legacies affected the response of fungal community composition to the drought in year 2 (i.e., we detected no significant 2‐way interactions). The variables that best explained shifts in fungal community composition were microbial biomass at peak drought, which decreased in the year 2 drought treatment (Figure 2c), and aboveground biomass and soil nitrate at recovery, which were higher in the fast‐strategy plant communities than the slow‐strategy plant communities (Figure 2d). The relative abundance of fungal families Mortierellaceae, Glomeraceae and Ceratobasidiaceae significantly increased when exposed to the year 2 drought, compared to the control (Figure S6g). Seven weeks after re‐wetting the year 2 drought, the shift in fungal community composition was related to increased N‐NO_3_^−^ in post‐droughted communities (Figure 2d, Table S2). However, the year 2 drought no longer significantly affected the relative abundance of fungal phyla or families 7 weeks after re‐wetting (Figure S6d,h, respectively).

### 3.3 Fungal α‐Diversity and Community Composition Differed Between Plant Communities With Slow or Fast Resource Acquisition Strategies

Plant communities with a slow resource acquisition strategy fostered significantly higher fungal α‐diversity than fast‐strategy communities in both the control and drought treatments at the ‘peak drought’ timepoint in year 2 (Figure S5b). Fungal community composition also significantly differed between plant communities with a slow or fast resource acquisition strategy throughout the experiment (Figure 2c,d, respectively). The plant community resource acquisition strategy did not affect the response of the fungal community, in terms of composition, to the year 2 drought (i.e., no significant interaction, Figure 2c,d). Plant community resource acquisition strategy did not affect prokaryote α‐diversity (Figure S5, Table S3) or prokaryote community composition (Figure 2a,b, Table S1). Nor did the plant community resource acquisition strategy modify soil legacy effects or the year 2 drought effects on prokaryote α‐diversity or composition (i.e., we found no significant interactive effects between the plant community resource acquisition strategy and drought in year 1 or year 2) (Figure 2a,b, Figure S5a).

### 3.4 Soil Legacies of Drought Intensity Affected Microbial Co‐Occurrence Network Structure

**FIGURE 3:**
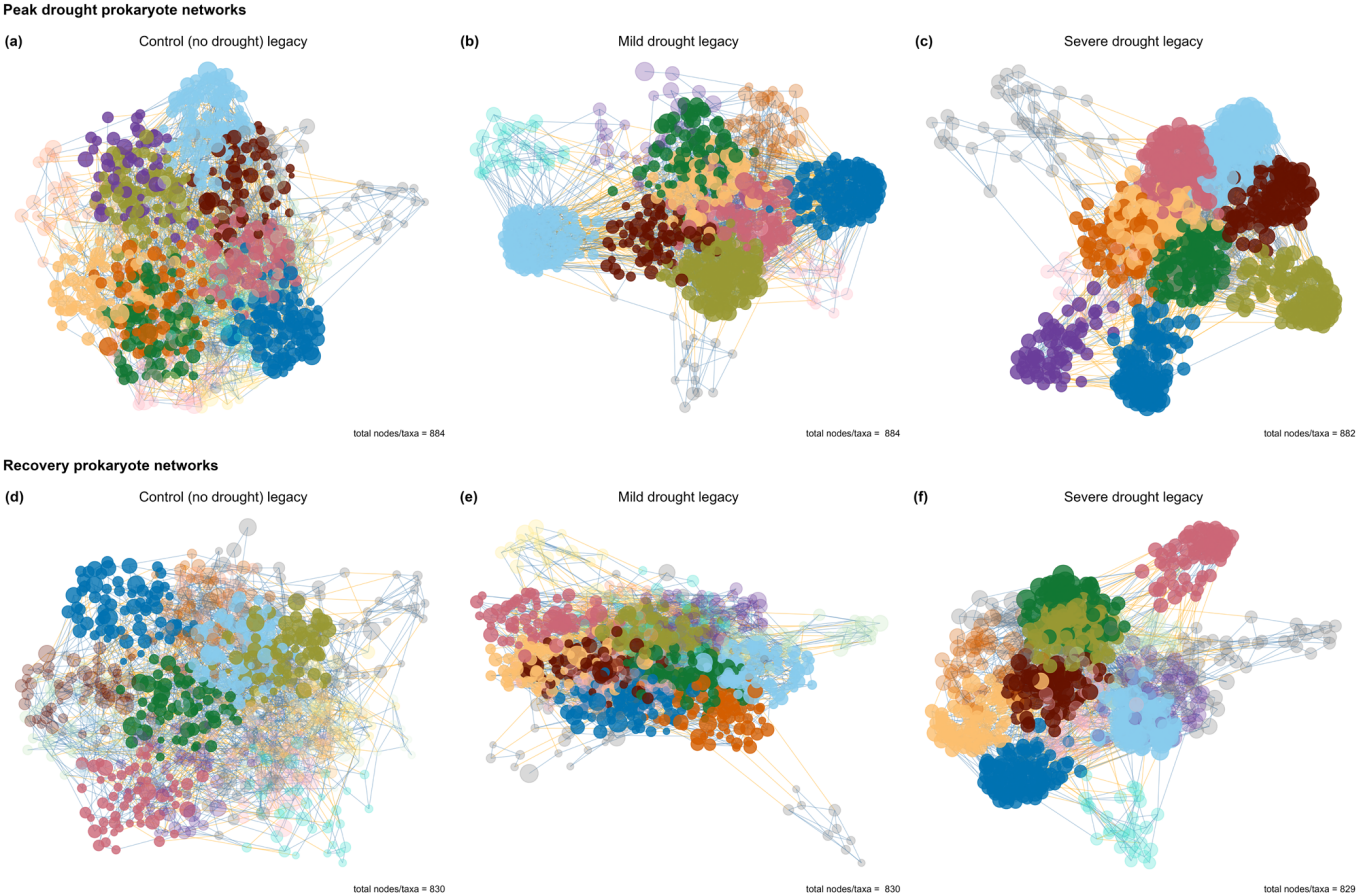
Soil legacies of past drought intensity (control, mild drought, or severe drought in year 1) affected prokaryote co‐occurrence network structure. Prokaryote networks at peak drought in year 2 in soil that had experienced (a) a control, (b) a mild drought, or (c) a severe drought in year 1, and at the recovery timepoint in year 2 that experienced (d) a control, (e) a mild drought, or (f) a severe drought in year 1. Networks are grouped by module (different colours). The size of the points/nodes indicates the relative abundance of the specific taxa, and the number of nodes/taxa in each network is specified in the caption.

**FIGURE 4:**
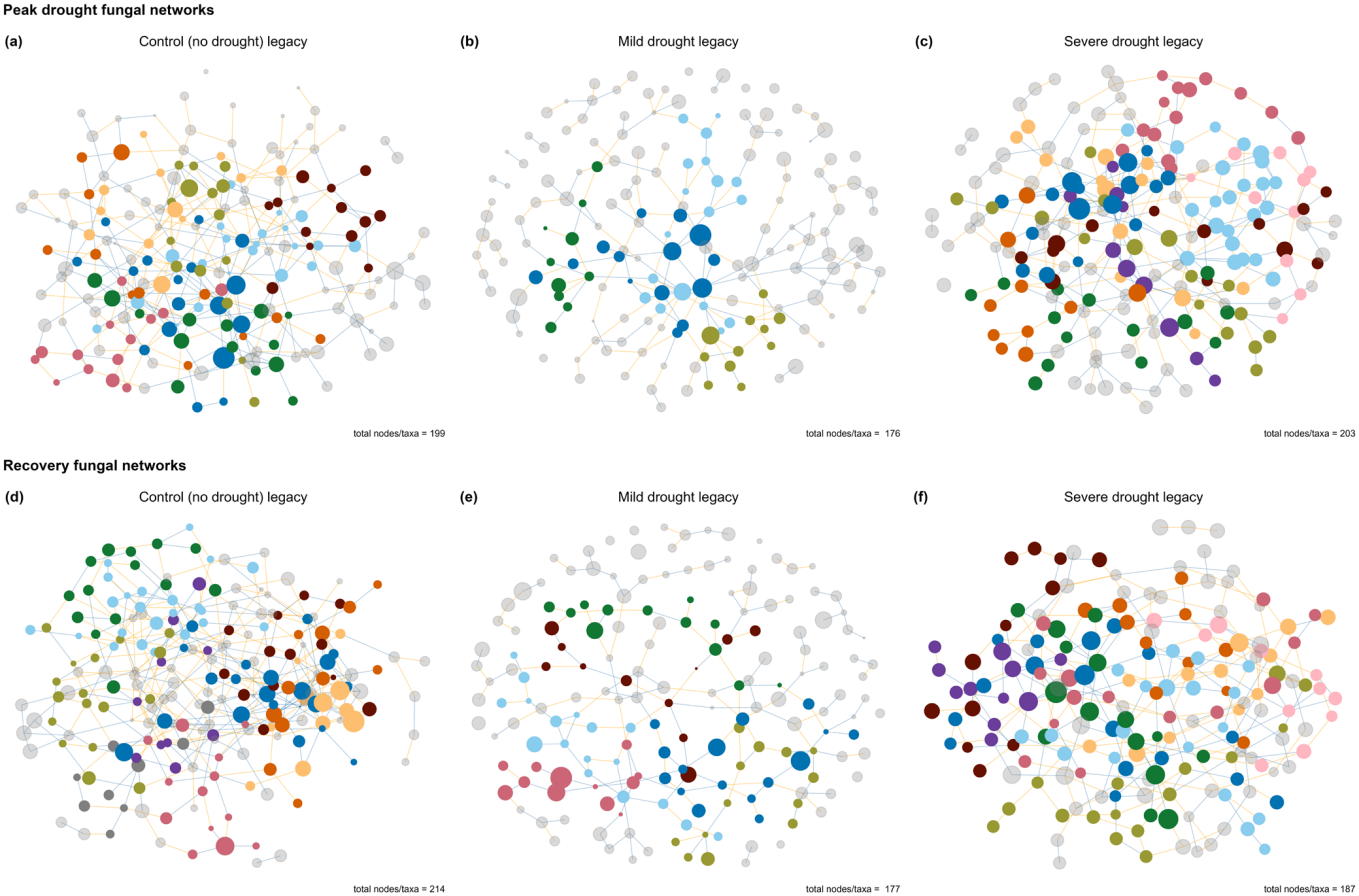
Soil legacies of past drought intensity (control, mild drought, or severe drought in year 1) affected fungal co‐occurrence networks. Fungal networks at peak drought in year 2 in soil that had previously experienced (a) a control, (b) a mild drought, or (c), a severe drought in year 1, and at the recovery timepoint in year 2 that experienced a (d) control, (e) a mild drought, or (f) a severe drought in year 1. Modules are indicated in different colours. The size of the points indicates the relative abundance of the taxa in the network, and the number of taxa/nodes per network is indicated in the caption.

We constructed co‐occurrence networks (Guseva et al. 2022) to determine how increasing drought intensity in year 1 affected prokaryote and fungal community structure in year 2 (Figures 3 and 4, respectively). Network analysis identifies groups of similarly responding (co‐occurring) taxa and provides information about structural characteristics such as complexity and stability (Barberán et al. 2012). We validated that network structure responded to soil legacies of increasing drought intensity because of node (i.e., taxa) identity rather than randomly by comparing observed network structural features to the distribution of 999 null models (the null model ensemble) following Caruso et al. (2022).

**FIGURE 5:**
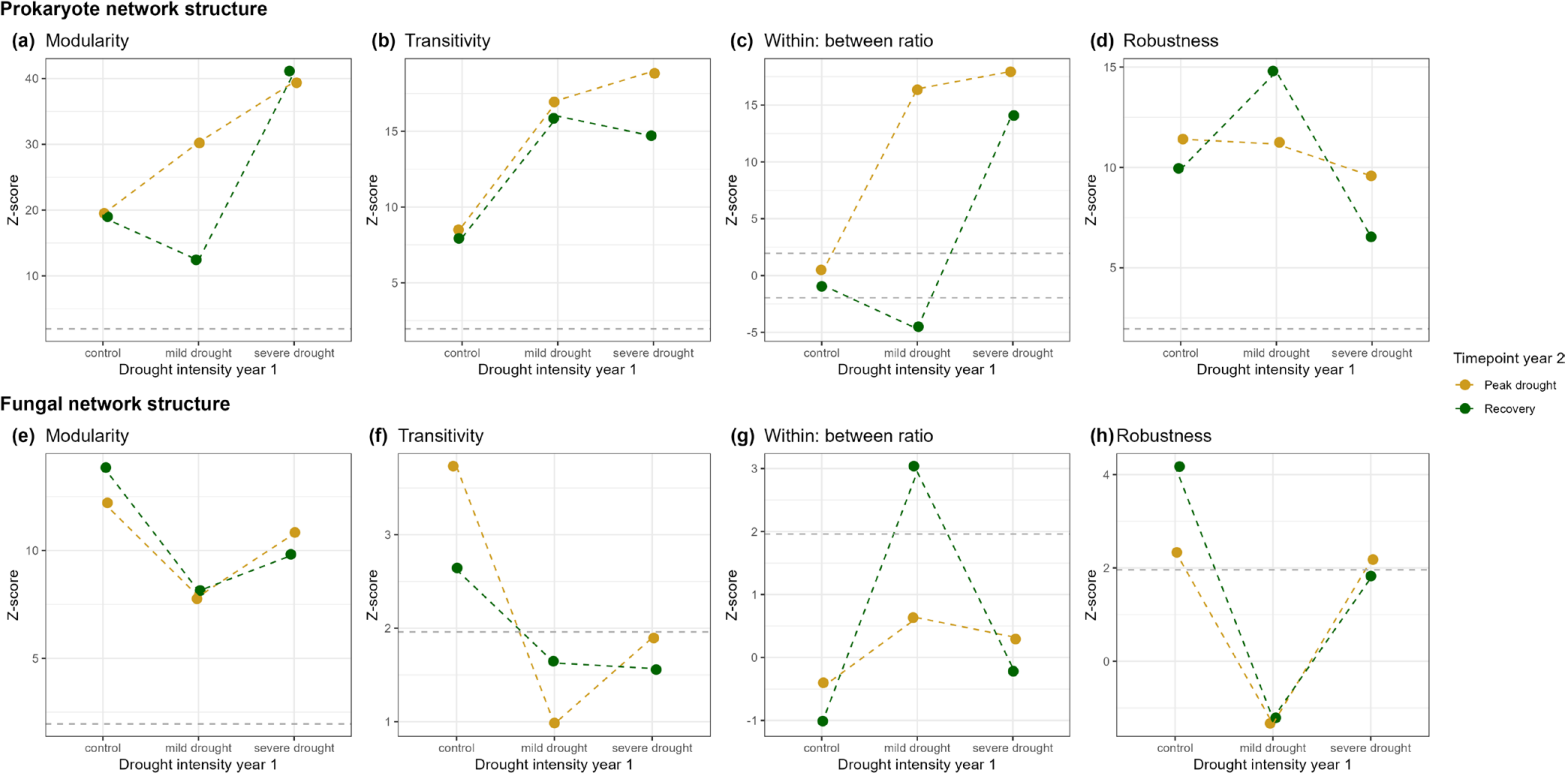
Soil drought legacies (drought intensity in year 1) affected co‐occurrence network topography. Network properties of prokaryote (a–d) and fungal (e–h) networks from communities in soil that had experienced either a control, mild drought, or severe drought in year 1 at the year 2 drought's peak drought or recovery timepoints. Points indicate the value of the observed network property; coloured dashed lines between points are for visual aid only and do not imply a statistical relationship. The *z*‐score denotes the difference between the observed network property and the null model ensemble. Values above 1.96 or below −1.96 (the dotted grey lines) indicate that the observed model property is significantly different from random. Modularity (a, e) indicates how modular the network structure is, while transitivity (b, f) is a measure of clustering, where higher values indicate a more modular or clustered network. The ratio of within‐to‐between module links (c, g) shows how connected a node (taxa) is to other taxa within the same module or to taxa in other parts of the network (between modules). Robustness (d, e) is the ability of the network to withstand targeted removal of nodes without collapsing. A higher value indicates a more stable network.

Prokaryote community network structure was significantly different from the null model ensemble, indicating that the links between nodes and node identity, rather than chance, drove network structure (Figure 5). At the peak drought and recovery timepoints in year 2, prokaryote networks in soil that had experienced a mild or severe drought in year 1 had higher network modularity and transitivity and a larger ratio of within:between links than prokaryote networks in soil that had experienced control conditions in year 1 (Figure 5a–c, respectively). This indicates that prokaryote networks that had a soil legacy of a severe drought were more complex than prokaryote networks in soil that was maintained at control conditions in year 1. Prokaryote network stability (i.e., network robustness) was significantly higher than the null model ensemble, indicating that the non‐random node arrangement and linkages increased network stability (Figure 5d). The prokaryote network that had a soil legacy of severe drought in year 1 was less robust than prokaryote networks that had a soil legacy of mild drought or control conditions in year 1 at the peak drought timepoint in year 2 (Figure 4d). At the recovery timepoint in year 2, the network that had a soil legacy of severe drought in year 1 was less robust than the network that experienced control conditions in year 1 (Figure 5d).

Fungal networks were significantly more modular than the null model ensemble, indicating non‐random co‐occurrence and network module membership. Fungal networks in soils exposed to a mild or severe drought in year 1 were less modular than the fungal network in soil that experienced a control in year 1 (Figure 5e). Only the fungal network in soil that experienced a control in year 1 had significantly higher transitivity than the null model ensemble, indicating that the connectedness of fungal networks in soil exposed to a mild or severe drought in year 1 is random (Figure 5f). Similarly, fungal network robustness only differed from the null model ensemble in the soils exposed to a severe drought or control in year 1 at the peak drought timepoint in year 2 (Figure 5h). Overall, fungal networks were less affected by soil drought legacies, in line with our findings that soil drought legacies have less pronounced effects on fungal community composition than on prokaryote communities (Figure 2c,d).

To define putative common functioning of subsections of the network, we clustered similarly responding microbial taxa into modules. Prokaryote network modularity was significantly different from the null model ensemble (Figure 5a), indicating non‐random module membership (Caruso et al. 2022). We identified shifts in the nitrifier community in the network modules. Specifically, we found that AOB taxa (members of the Nitrosomonadaceae and Nitrospiraceae families) were generally present in the same module, while AOA taxa (members of the Nitrosospheraceae family) were present in other modules at both timepoints (Figures S7 and S8). At the recovery timepoint in year 2, this difference was especially pronounced in the prokaryote networks in soils that had experienced a mild or severe drought in year 1 (Figure S8b,c, respectively). This indicates that AOB responded differently than AOA to soil legacies of increasing drought intensity.

Fungal membership in the largest modules (Figure 4) differed depending on the soil drought legacies (Figure S9). At the peak drought timepoint in year 2, taxa within the Mortierellaceae family dominated the second largest modules in all networks (Figure S9a–c), while taxa from the Mortierellaceae family were less present at the recovery timepoint in year 2 (Figure S9d–f).

### 3.5 Soil Legacies of Increasing Drought Intensity Affected Soil Functioning in Year 2

**FIGURE 6:**
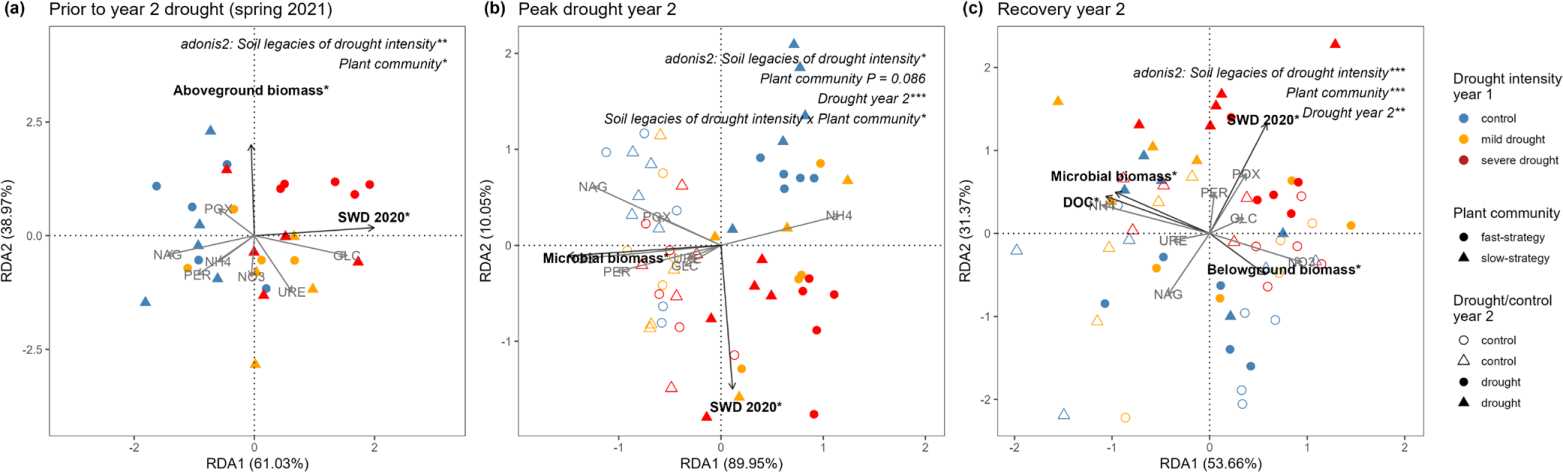
Soil legacies of past drought intensity affected soil functioning in year 2. Constrained ordination (RDA) showing effects of soil legacies of increasing drought intensity (control, mild drought, or severe drought in 2020), plant community resource acquisition strategy (fast or slow), and drought (or control) in year 2 on soil functioning (a) in the spring before the drought in year 2 (b) at peak drought year 2 and (c) at recovery year 2 (7 weeks after re‐wetting). Points indicate the potential extracellular enzyme activity (pEEA) of five enzymes: β‐glucosidase (GLC), β‐N‐acetylglucosaminidase (NAG), urease (URE), peroxidase (PER) and phenoloxidase (POX) and the soluble nitrogen (N‐NO_3_^−^ and N‐NH_4_^+^) in each mesocosm. We first included the same set of explanatory variables in each model and determined the best model with forward selection based on model adjusted *R* ^2^ (see Methods). Final explanatory variables in the models were: soil water deficit in 2020 (SWD 2020), and plant community aboveground biomass (Aboveground biomass), belowground/root biomass (Belowground biomass), microbial biomass and dissolved organic carbon (DOC). A star indicates significance of the explanatory variable (arrow), tested with anova.cca. Significance of the independent variables (soil legacies of drought intensity, drought year 2 and plant community resource acquisition strategy) are indicated as follows: *P* < 0.001***, *p* < 0.01**, *p* < 0.05*. All interactions were tested and if significant, are reported in the figure. For full statistical output, see Table S5.

We found that soil legacies of increasing drought intensity significantly affected soil functioning throughout year 2 (Figure 6, Table S5). In the spring prior to the year 2 drought treatment, we found that soil functioning depended on soil legacies of increasing drought intensity (SWD 2020) and plant community aboveground biomass (Figure 6a; Table S5a). This was also visible in the univariate relationships, where pGLC significantly increased in soil that had been exposed to a mild or severe drought in year 1 (Figure S10d, Table S6), and pNAG significantly decreased in soil that was exposed to a severe drought, compared to a control in year 1 (Figure S10e, Table S6).

The year 2 drought significantly affected soil functioning at peak drought (Figure 6b, Table S5b). This was regardless of soil legacies of increasing drought intensity, i.e., there were no significant interactive effects on multivariate soil functioning (Figure 6b, PERMANOVA, Table S5). We found a significant interaction between plant community resource acquisition strategy and soil drought legacies; however, soil legacies had a similar effect on multivariate soil functioning in both plant communities (Figure 6b, Table S5). We found that in year 2, the drought increased N‐NH_4_^+^ (Figure S10i, Table S6) and decreased N‐NO_3_^−^, microbial biomass carbon, pNAG, the potential activity of phenol oxidase (pPOX, Figure S10, Table S6), and the potential activity of peroxidase (pPER, Figure S10p, Table S6). Soil legacies of increasing drought intensity tended to affect the response of pGLC (Figure S6l, Table S6) and pURE (Figure S10n, Table S6) to drought in year 2. In both cases, we found that the year 2 drought significantly decreased pGLC and pURE only when the soil was maintained at control conditions in year 1. Based on constrained ordination, we found that soil legacies of increasing drought intensity (SWD in 2020) and microbial biomass explained significant variation in soil functioning at peak drought in year 2 (Figure 6b, Table S5e).

At the recovery timepoint of the year 2 drought, soil functioning differed between plant communities and was affected by soil legacies of increasing drought intensity and the drought in year 2 (Figure 6c, Table S5c). There were soil legacies of increasing drought intensity, indicating that the past drought did not affect the recovery response of multivariate soil functioning (Figure 6c, Table S5c). Based on constrained ordination, SWD 2020, belowground biomass, DOC and microbial biomass explained significant variation in soil functioning (Figure 6c, Table S5f). The recovery response of pPER to the drought in year 2 was modified by soil legacies of increasing drought intensity (Figure S10x, Table S6). When the soil had been exposed to a mild or severe drought in year 1, there was overcompensation in pPER following the drought in year 2 (Figure S10x, Table S6). Compared to the slow‐strategy communities, fast‐strategy plant communities had significantly lower N‐NH_4_^+^ (Figure S10q, Table S6), pPOX (Figure S10w, Table S6), and pPER (Figure S10x, Table S6) and significantly higher N‐NO_3_^−^ (Figure S10r, Table S6). Fast‐strategy plant communities also had significantly lower microbial biomass carbon in soil that had experienced the mild drought in year 1 (Figure S10s, Table S6).

### 3.6 Past Drought Intensity Affected Plant Community Response to the Second Drought

**FIGURE 7:**
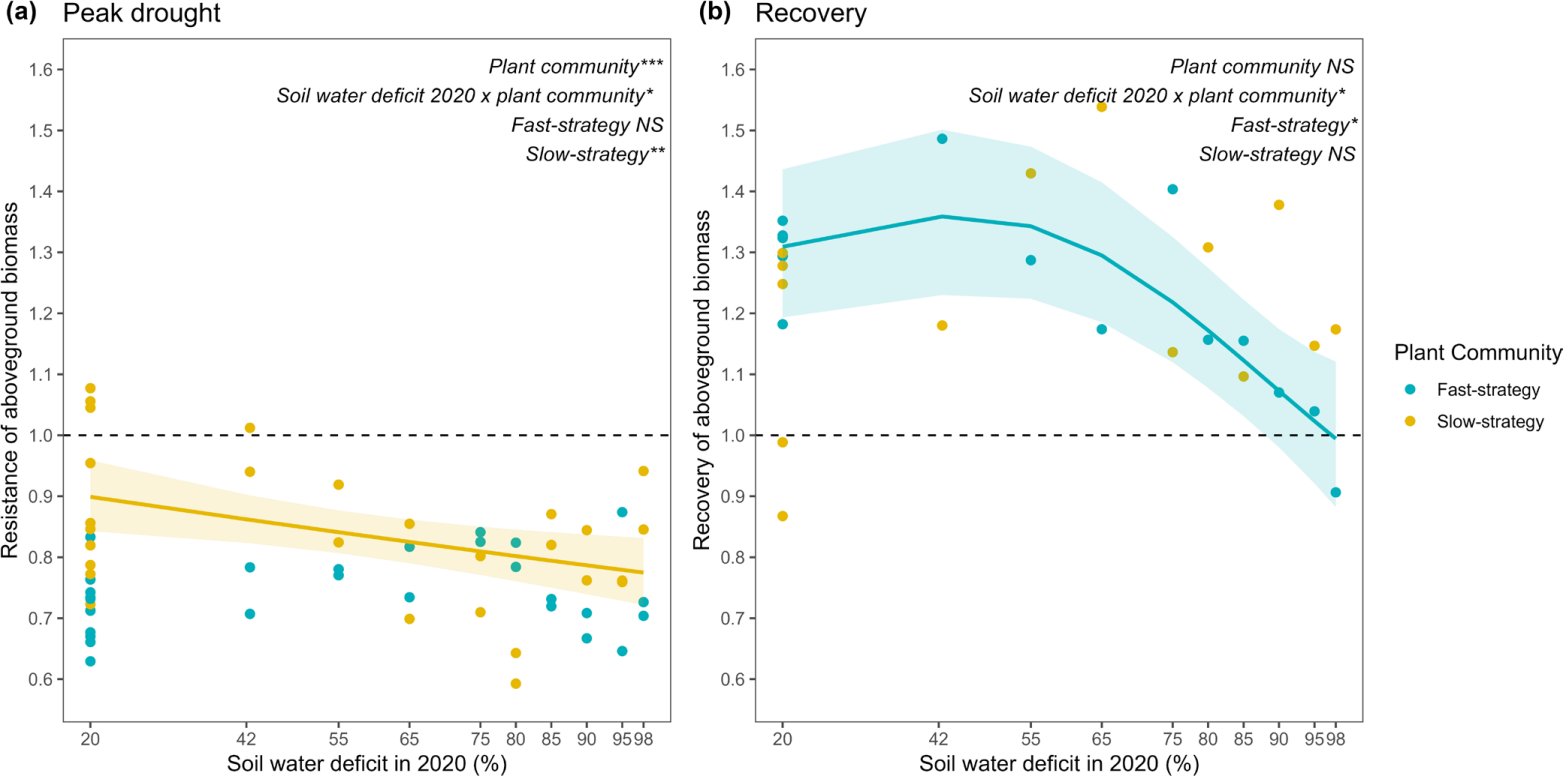
Soil legacies of increasing drought intensity (soil water deficit in 2020/year 1) affected plant community productivity response to drought in year 2. Effects of the soil water deficit in 2020 on (a) the resistance of aboveground biomass at the peak drought in year 2, and (b) the recovery of aboveground biomass 7 weeks after re‐wetting in year 2. Baseline normalised resistance and recovery were calculated using the mean aboveground biomass of communities that were maintained at control conditions (soil water deficit of 20% in year 1 and control in year 2) as the continuous baseline following Ingrisch and Bahn (2018). Resistance was quantified after 3 weeks of drought, and recovery was quantified 7 weeks after re‐wetting. The dashed line at 1.0 indicates no difference in aboveground biomass between drought and control treatments in year 2. Significance was tested using generalised additive models to account for nonlinear responses following Ingrisch et al. (2023). The effect of soil water deficit in 2020, plant community resource acquisition strategy, and their interaction were tested, and a second model tested the effect of soil water deficit within each plant community to understand the significant interaction effect. *p* < 0.05*, *p* < 0.01**, *p* < 0.001***; for statistics, see Table S7.

Soil legacies of increasing drought intensity (SWD 2020) significantly affected plant community resistance to and recovery from the drought in year 2, and this effect depended on plant community resource acquisition strategy (Figure 7, Table S7). Soil legacies of increasing drought intensity decreased slow‐strategy resistance of aboveground biomass to drought in year 2 and reduced the overshoot of aboveground biomass in the fast‐strategy community afterwards (Figure 5, Table S7). The plant community with a slow resource acquisition strategy was significantly more resistant to the year 2 drought than the fast resource acquisition strategy plant community, while there was no difference in post‐drought overshoot between the plant communities (Figure 7, Table S7).

**FIGURE 8:**
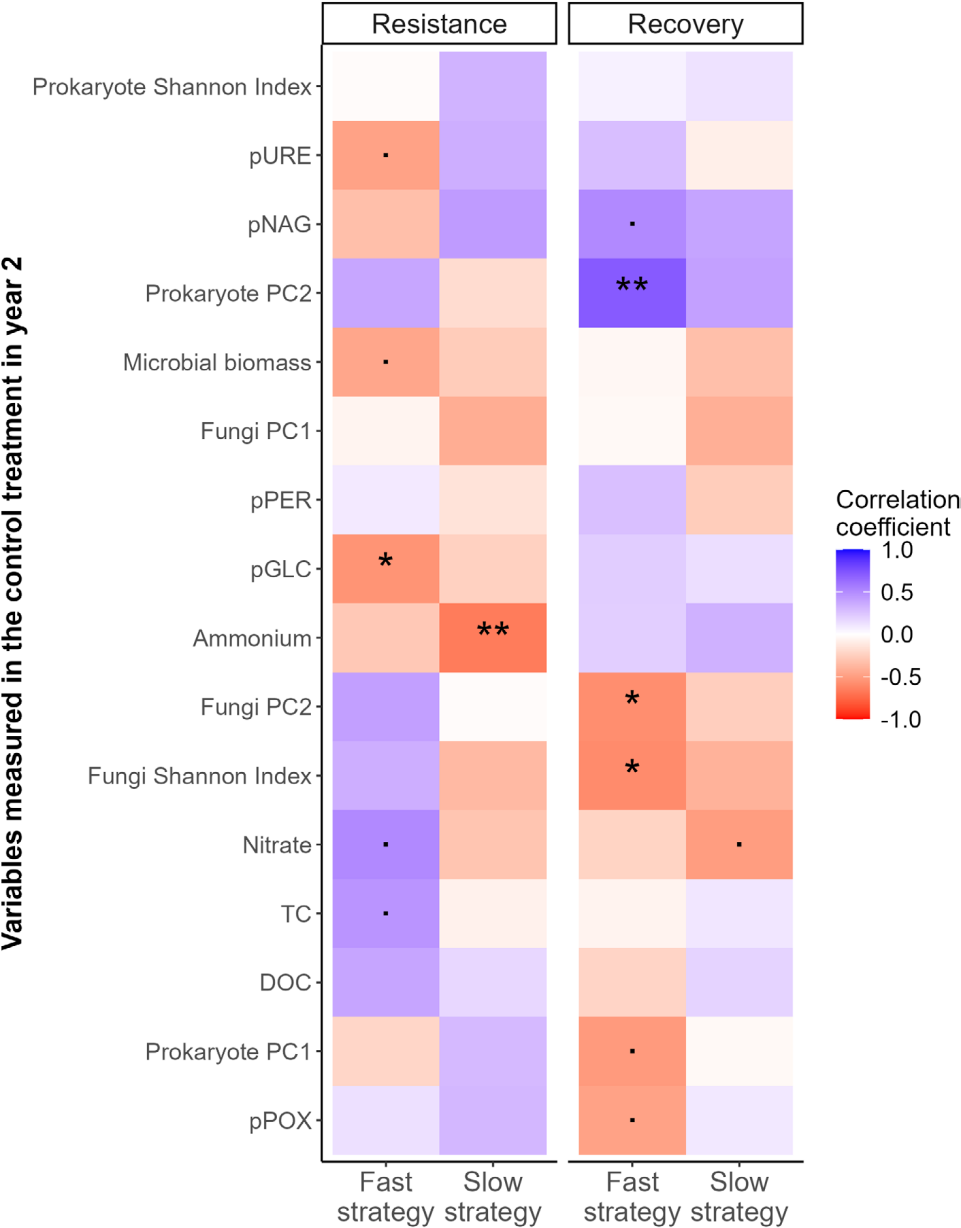
Rank correlations between soil drought legacy effects on soil variables in control conditions in year 2 and plant community resistance and recovery from the year 2 drought. We related soil variables measured in the control treatment in year 2, i.e., communities that had experienced the drought intensity gradient in year 1 but no drought in year 2, to the resistance and recovery of community aboveground biomass in fast and slow‐strategy plant communities in year 2. In this way, we can determine how soil drought legacy effects predict plant community response to subsequent drought. The order of the soil variables measured in the year 2 controls (y‐axis) was determined by complete‐linkage clustering according to their correlations with plant community resistance and recovery. Colours indicate the direction of the Spearman rank correlation, from negative (red) to positive (blue), and stars indicate the significance: *p* < 0.05*, *p* < 0.01**. We used principal component analysis to capture prokaryote and fungal community composition (see Figure S7), shown here as fungi PC1, PC2 and prokaryote PC1, PC2. The Shannon Index was used to quantify the alpha diversity of prokaryotic and fungal communities (see Figure S1). As measures of microbial community functioning, we include potential extracellular enzyme activity: β‐glucosidase (pGLC), β‐N‐acetylglucosaminidase (pNAG), urease (pURE), peroxidase (pPER) and phenoloxidase (pPOX), and soluble nitrogen pools (nitrate, N‐NO_3_^−^ and ammonium, N‐NH_4_^+^) and dissolved organic carbon (DOC) and total dissolved carbon (TC) as measures of the nitrogen and carbon pools, respectively.

We determined whether soil legacies of increasing drought intensity predict plant community response to a subsequent drought by relating soil variables measured in control mesocosms in year 2 (and thus, variation is caused only by soil drought legacies) to the resistance and recovery of plant community aboveground biomass using Spearman rank correlations (Figure 8). At peak drought in year 2, we found that decreases in soil N‐NH_4_^+^ were significantly negatively related to slow‐strategy community drought resistance. While the soil legacies of increasing drought intensity did not significantly affect fast‐strategy drought resistance in year 2 (Figure 7), we found that soil drought legacy effects on pGLC were significantly negatively related to fast‐strategy community resistance, while pURE and microbial biomass tended to be negatively related (Figure 8). Higher nitrate and total dissolved carbon were significantly positively related to fast‐strategy community resistance. Seven weeks after re‐wetting, we found that prokaryote community composition (PC2 axis scores, Figure S11) was significantly related to fast‐strategy community recovery. We also found that increasing fungal alpha diversity (Shannon index, Figure S5) was related to lower overshoot in the fast resource acquisition strategy plant community. Fungal community composition (PC2 axis scores, Figure S11) was significantly negatively related to fast resource acquisition strategy community overshoot (Figure 8). Slow resource acquisition strategy community recovery (overshoot) was not significantly related to any of the soil variables we measured, and thus, soil legacies of increasing drought intensity on soil abiotic and biotic parameters could not explain slow resource acquisition strategy recovery of aboveground biomass (Figure 8).

## 4 Discussion

We found that increasing the intensity of a past drought shaped soil legacy effects on soil microbial community structure and function. Moreover, soil legacies affected plant community response to a subsequent drought: increasing past drought intensity decreased resistance of the slow resource acquisition strategy plant community and decreased the recovery of the fast resource acquisition strategy plant community from a subsequent drought. These findings advance our understanding of drought legacy effects on plant and soil microbial communities by isolating drought legacy effects via the soil and demonstrating how the impact of soil drought legacies depends on past drought intensity.

### 4.1 Drought Intensity Shapes Soil Legacy Effects on Soil Microbial Communities

The impact of the soil drought legacies on prokaryote and fungal communities increased with increasing past drought intensity. Additionally, the soil drought legacy effects were more pronounced in prokaryote than fungal communities. This is broadly in line with our previous findings that the concurrent (initial) effects of increasing drought intensity are stronger in bacterial than in fungal communities (Oram et al. 2025) and that bacterial communities are more drought sensitive than fungal communities (de Vries et al. 2018; Ochoa‐Hueso et al. 2018; Preece et al. 2019). Fungal communities have also been reported to be less responsive to long‐term precipitation legacies (Tang et al. 2023).

We found that soil legacies of increasing past drought intensity increased prokaryote network complexity and decreased network stability. This is in contrast to the classical ecological theory that complexity begets stability (MacArthur 1955) and recent research showing increases in bacterial network complexity result in increased network stability (Yuan et al. 2021). We found that prokaryote networks became more complex due to higher modularity with increasing past drought intensity, with more links within than between modules, creating dense subgroups within the network. This modular structure suggests that taxa within these large sub‐groups (modules) respond similarly to environmental variation caused by the soil drought legacies, leading to both coupling and decoupling of taxa responses. Consequently, when a disturbance occurs, large sections of the network respond in tandem, making the network highly dynamic, more stochastic and less stable (In 't Zandt et al. 2023; May 1974, 197). As such, our findings provide evidence that an increased drought intensity leads to soil legacy effects that reduce the stability of soil prokaryote networks in response to subsequent disturbances, including drought.

We identified shifts in the nitrifier community, from a higher abundance of AOB under low past drought intensity to a higher relative abundance of AOA under high past drought intensity. These shifts were apparent both in the prokaryote β‐diversity and in the network structure, where the clustering of AOB and AOA increased with increasing past drought intensity. Similar decreases in Proteobacteria (the phylum including AOB) in grasslands with drought history have been reported (Canarini et al. 2021); however, our finding of increased AOA (and Crenarchaeota) abundance following severe drought contrasts with previous studies that reported drought‐associated declines in AOA abundance (Séneca et al. 2020; Thion and Prosser 2014). In our study, the soil legacy effects of increasing drought intensity on AOA may have developed in the recovery period following the drought intensity gradient in year 1 because of shifts in plant community composition to a more grass‐dominated system (de Vries et al. 2018; Oram et al. 2025). AOA have been found to be more abundant than AOB in the rhizosphere of grass species (compared to forbs), likely due to their preference for lower ammonium concentrations. This trait allows AOA to outcompete AOB in the rhizospheres of plants with high N demand, where ammonium availability tends to be lower (Thion et al. 2016).

Overall, soil legacies of past drought intensity affected fungal community composition. However, this effect was less pronounced than in prokaryote communities, and we found no significant shifts in the relative abundance of individual fungal families or phyla and small effects of soil drought legacies on fungal network structure. This is broadly in line with de Vries et al. (2018), who found that drought destabilises bacterial but not fungal networks, and the finding that fungi are more drought tolerant than bacteria (Barnard et al. 2013). Our findings are the first to demonstrate that soil legacies of increasing drought intensity have more muted effects on soil fungal communities, compared to prokaryote communities.

In line with our hypothesis, we found that soil legacies of increasing drought intensity affected soil functioning, similar to drought legacy effects in field studies (Broderick et al. 2025; Fuchslueger et al. 2016; Leizeaga et al. 2021). Soil legacies of increasing drought intensity reduced pNAG activity, increased pPOX activity and affected pURE and pPER responses to drought. Notably, the increase in pPOX and pPER activities with increasing past drought intensity suggests a shift in microbial community composition to microbes with the ability to degrade lignin. This finding, which is consistent with past work showing that drought legacies result in increased degradation of more chemically complex substrates (Canarini et al. 2021), may be related to the release of easily degradable substrates following re‐wetting of the past drought event, which was likely more pronounced with increasing drought intensity. Thus, in the second growing season, soil previously exposed to a severe drought could have lower availability of these easier‐to‐degrade carbon sources, i.e., a dampened Birch effect, as has been shown in previous research (Kaisermann et al. 2017). In the longer term, increases in oxidative enzyme activity could lead to soil carbon losses and reduced soil carbon accumulation (Chen et al. 2018).

### 4.2 Plant Community Resource Acquisition Strategy Did Not Mediate Soil Drought Legacy Effects on Microbial Community Composition

In contrast to our second hypothesis, we found no significant interactive effects of plant community resource acquisition strategy and soil legacies of increasing drought intensity on prokaryote or fungal community composition or richness. This is surprising because fast‐ versus slow‐strategy plants have been found to differ in their belowground carbon allocation and nitrogen uptake (Henneron, Cros, et al. 2020; Henneron, Kardol, et al. 2020), which leads to diverging rhizosphere microbial communities (Guyonnet et al. 2017, 2018) that are predicted to differ in their drought responses (Williams and de Vries 2020). Our results show that the fast‐ and slow‐strategy plant communities fostered significantly different fungal communities. Previous research has found that plant strategy explains fungal community composition (Semchenko et al. 2018; Sweeney et al. 2021). The slow resource acquisition strategy plant community fostered a more diverse fungal community at the peak drought timepoint (higher α‐diversity in both the drought and control treatments) and increased the abundance of the mycorrhizal family Diversisporaceae (Redecker et al. 2013). Slow resource acquisition strategy plants are reported to be more reliant on mycorrhizal symbioses for nutrient uptake (Bergmann et al. 2020; Wen et al. 2022), which could explain why they fostered a higher abundance of Diversisporaceae taxa. However, this result should be taken with caution, as we targeted the ITS2 region in this study, which is not the optimal method to quantify arbuscular mycorrhizal community composition (while it is robust for determining overall fungal community composition). Further, our study includes two model plant communities, and therefore the effect of plant community composition cannot be separated from the effect of resource acquisition strategy. A more robust test of plant strategy should consider the natural variation in fast versus slow‐strategy communities by including effects that would include multiple plant species combinations within each resource acquisition strategy.

### 4.3 Past Drought Intensity Affects the Drought Responses of Plant Productivity, but Not Microbial Communities

We found that soil legacies of past drought intensity reduced slow‐strategy plant resistance to the subsequent drought and fast‐strategy plant overshoot 7 weeks after re‐wetting. This finding advances earlier research by isolating soil drought legacy effects on plant response to a subsequent drought and demonstrates that these effects are driven by past drought intensity. Our results support earlier studies showing soil drought legacies affect plant growth (De Long, Semchenko, et al. 2019) and response to a subsequent drought (Kaisermann et al. 2017) and build on this research by providing clear evidence that drought intensity is a key driver of soil drought legacy effects on plant productivity and drought resilience.

We found that soil N‐NH_4_^+^ significantly predicted the drought resistance of slow resource acquisition strategy plant communities: soils with higher N‐NH_4_^+^ in the year 2 control treatment were related to lower plant community resistance to the year 2 drought. Higher concentrations of N‐NH_4_^+^ could signal a reduction in nitrification, leading to decreased availability of N‐NO_3_^−^ for plant uptake, thereby reducing drought resistance. Although nitrification rates were not directly measured, we observed shifts in nitrifier communities with increasing past drought intensity: AOB significantly decreased in relative abundance, while AOA relative abundance increased (see discussion above). Thus, the potential of the prokaryote community to convert NH_4_^+^ to NO_3_^−^ may have been compromised in soils previously subjected to severe drought, resulting in reduced plant N uptake in soils and consequently lower drought resistance. Plant communities with a slow resource acquisition strategy may have been more affected than plant communities with a fast resource acquisition strategy because they maintained growth during drought periods (i.e., slow‐strategy plants generally show higher drought resistance).

Our results show that variation in prokaryote community composition captured by the PC2 axis, rather than the dominant variation represented by PC1, best predicted fast‐strategy community overshoot. This suggests that specific microbial groups, rather than broad community shifts, are key predictors of plant recovery dynamics. Previous research has demonstrated that soil microbial community composition affects the impact of drought on plant performance (Buchenau et al. 2022), with dry‐adapted microbiota alleviating negative drought effects on plant biomass (O'Brien et al. 2018). However, soil legacies of severe drought intensity reduced the ability of the fast resource acquisition strategy plant community to overshoot (in terms of aboveground biomass), indicating that drought‐induced changes in the prokaryote community composition dampen this response. Plants with a fast resource acquisition strategy are frequently shown to capitalise on the Birch effect—i.e., the increase in N availability shortly after re‐wetting (de Vries et al. 2018; Lavallee et al. 2024). However, Birch effects (in terms of C and N) have been shown to be less pronounced when soils have previously experienced drought (Kaisermann et al. 2017). This reduction may be partly driven by drought legacy‐induced shifts in the prokaryote community composition (see discussion above) and their ability to cycle C and N (Fuchslueger et al. 2016; Hawkes and Keitt 2015). Consistent with this, we observed reduced activity of pNAG in soils previously exposed to severe drought. pNAG activity has been positively related to soil N (Cenini et al. 2016), organic N acquisition by the microbial community (Sinsabaugh et al. 2009), N turnover (Burns et al. 2013) and gross N mineralisation rates (Darby et al. 2020), which could reduce plant N uptake during early recovery and may influence the magnitude of productivity overshoot later on.

In contrast to our hypothesis, soil legacies of increasing drought intensity did not affect prokaryote or fungal community response to a subsequent drought. Previous research has shown that a history of repeated drought influences microbial response to subsequent drying and re‐wetting events (De Nijs et al. 2019; Leizeaga et al. 2021; Tang et al. 2023), but also that this effect develops over longer time scales rather than in response to a single drought event (Canarini et al. 2021). Thus, with more frequent intense droughts, soil legacy effects on microbial responses to subsequent stress events are likely to become more pronounced. Regarding microbial community functioning, we found that increasing the intensity of the past drought enhanced the pPER recovery from the subsequent drought. An increase in the potential activity of lignin oxidises (such as peroxidase, pPER, or phenoloxidase, pPOX; see discussion above) can indicate a shift in the microbial community towards a greater abundance of organisms that can break down lignin. This shift can alter decomposition dynamics and soil C cycling (Janusz et al. 2017), leading to higher soil carbon losses and reduced soil carbon accumulation (Chen et al. 2018). Additionally, we identified a buffering effect of soil legacies from past drought intensity on the activity of pURE and pGLC, which only decreased in microbial communities that were not previously exposed to drought in year 1. This aligns with previous studies showing that drought legacies modulate microbial functional responses to subsequent droughts (Evans and Wallenstein 2012; Fuchslueger et al. 2016; Kaisermann et al. 2017) and provides evidence that increasing intensity of the past drought has lasting effects on soil functioning, increasing the functional capacity of microbial communities to degrade complex carbon sources.

## 5 Conclusions

Soil legacies of increasing drought intensity had increasingly pronounced effects on prokaryote and fungal community composition, restructured prokaryote networks and compromised their stability in the following year, with repercussions for soil functioning. We show that these soil legacies of increasing drought intensity affect the resistance and recovery of plant community productivity (aboveground biomass) to a subsequent drought, with responses varying according to plant community resource acquisition strategy. We also provide evidence that soil microbial communities and functions explain variation in plant community resistance and recovery from a subsequent drought. Our findings indicate that as intense droughts become more frequent in our rapidly changing climate, soil legacy effects will play an increasingly prominent role in grassland functioning and stress responses.

## Author Contributions

**Natalie J. Oram:** conceptualization, formal analysis, investigation, writing – original draft, writing – review and editing. **Nadine Praeg:** conceptualization, formal analysis, investigation, writing – original draft, writing – review and editing. **Richard D. Bardgett:** conceptualization, writing – original draft, writing – review and editing. **Fiona Brennan:** conceptualization, writing – original draft, writing – review and editing. **Tancredi Caruso:** conceptualization, formal analysis, writing – review and editing. **Paul Illmer:** conceptualization, writing – review and editing. **Johannes Ingrisch:** conceptualization, writing – review and editing. **Michael Bahn:** conceptualization, investigation, writing – original draft, writing – review and editing.

## Conflicts of Interest

The authors declare no conflicts of interest.

## Supporting information


**Figure S1:** The soil water deficit (SWD, % of field capacity) during the drought period in 2020 (year 1), which is referred to in this study as soil legacies of increasing drought intensity. This figure is originally published in Oram et al. (2023) and reprinted in Oram et al. (2025). (A) Realised SWD indicates the measured SWD over time (day of the calendar year). The grey horizontal line at 20% realised SWD indicates the target control, the colours indicate the target SWD. (B) The relationship between realised and target SWD at peak drought (225 days of the calendar year). The diagonal dashed line indicates the 1:1 line, the solid black line is a linear regression and the *R* ^2^ indicates the adjusted *R* ^2^ of the linear regression between realised and target SWD.
**Figure S2:** Climatic conditions throughout the experimental period in 2021. (A) Daily precipitation, (B) air temperature and (C) vapour pressure deficit, (Year‐Month‐Day). Data were obtained from GeoSphere Austria (https://data.hub.geosphere.at).
**Figure S3:** Dry‐down dynamics in 2021 for the fast‐ and the slow‐strategy communities in the control and the drought treatment during the 3‐week drought period. Soil water deficit (SWD) indicates the percent deficit from field capacity. Points are jittered for visualisation.
**Figure S4:** Sequencing depth. Rarefaction curves of prokaryote (A, B) and fungal (C, D) taxa based on sequence reads in soil samples taken after 21 days of drought (peak drought) and 7 weeks after re‐wetting (recovery). Species refers to the number of taxa; sample size indicates the number of reads. Each line represents one experimental unit/sample, indicated by the number in the square.
**Figure S5:** Prokaryote and fungal α‐diversity (Shannon Index). The effect of soil legacies of increasing drought intensity (control, mild drought, or severe drought in year 1), plant community (fast‐ or slow‐strategy), and drought (or control) in year 2 on the Shannon Index of prokaryote (A) or fungal (B) communities at peak drought in year 2 and the prokaryote (C) or fungal (D) communities 7 weeks after re‐wetting. Captions indicate significance based on ANOVA (see Table S3 for complete statistical output).
**Figure S6:** The relative abundance of prokaryote and fungal phyla and families. Soil legacies of increasing drought intensity (control, mild drought, or severe drought in year 1), drought or control in year 2, and their interactive effects on the relative abundance of prokaryote and fungal phyla (A–D) and the 20 most abundant families, excluding ‘unknown’ families (E–H) at the peak drought and recovery timepoints in year 2. Shades of the same colour indicate that the family belongs to the same phylum. Significance was tested using linear models and ANOVA (see Methods). Symbols beside the family name in the legend denote the significance of experimental treatments in the following order: Soil legacies of increasing drought intensity in control conditions in year 2, soil legacies of increasing drought intensity in drought conditions in year 2, the year 2 drought versus control, and the interaction between soil legacies and the year 2 drought or control (*p* < 0.05*, *p* > 0.05−), see Table S4 for statistical output.
**Figure S7:** Prokaryote network module membership at peak drought year 2. Prokaryote module membership in the largest six modules at the peak drought timepoint in year 2 in the networks exposed to (A) a control, (B) mild drought and (C) severe drought in year 1. The colour indicates the family (also indicated on the Y axis); the number beside the bar denotes the number of ASVs within each family. The title of each panel indicates the module number and the number of nodes (ASVs) included in that module. For aesthetic clarity, only the 10 most abundant families (based on read number) are shown.
**Figure S8:** Prokaryote network module membership at recovery year 2. Prokaryote module membership in the largest six modules at the recovery timepoint in year 2 in the networks exposed to (A) a control, (B) mild drought and (C) severe drought in year 1. The colour indicates the family (also indicated on the Y axis); the number beside the bar denotes the number of ASVs within each family. The title of each panel indicates the module number corresponding to that in Figure 2D–F, along with the number of nodes (ASVs) included in that module. For aesthetic clarity, only the 10 most abundant families (based on read number) are shown in the figure.
**Figure S9:** Fungal network module membership in year 2. Fungal module membership in the largest three modules per network at the (A–C) peak drought and (D–F) recovery timepoints in year 2, in fungal networks in soil that experienced either a control, mild drought, or severe drought in year 1. The colour indicates the family (also indicated on the Y axis); the number beside the bar denotes the number of ASVs within each family. The title of each panel indicates the module number and the number of nodes (ASVs) included in that module. All families with membership in a module are shown.
**Figure S10:** Soil functioning in year 2 (univariate relations). Soil ammonium (N‐NH_4_), nitrate (N‐NO_3_), microbial biomass and the potential enzyme activity of β‐glucosidase (pGLC), β‐N‐acetylglucosaminidase (pNAG), urease (pURE), peroxidase (pPER) and phenoloxidase (pPOX) before the subsequent drought (A–H), at peak drought (I–P) and at recovery (Q–X). Effects of the drought intensity in year 1 (called here soil legacy for brevity: control, mild drought, or severe drought in 2020), the drought (or control) in year 2 (blue or yellow points), plant community (fast‐ or slow‐strategy), and all 2‐way interactions were tested with linear models (N‐NH_4_, N‐NO_3_, microbial biomass) and linear mixed effects models (all potential enzyme activities to account for laboratory replicates). Letters indicate significant differences between levels of a treatment based on a Tukey post hoc test. *p* < 0.05*, *p* < 0.01**, *p* < 0.001***.
**Figure S11:** Principal component analysis of microbial communities in the control treatment in year 2 at the ‘peak drought’ and ‘recovery’ timepoints. Note that these communities were not exposed to the year 2 drought but were maintained at control soil moisture throughout year 2. Read numbers were centre log ratio transformed before principal component analysis using Euclidean distances. Shapes indicate the plant community (fast‐ or slow‐strategy) and colour indicates the soil drought legacy (control, mild drought, or severe drought in year 1). Scores of the principal component axes (PC1, PC2) of prokaryote and fungal communities were used to explain the resistance and recovery of plant community aboveground biomass to the drought in year 2 (see Figure 8 in the main text).
**Figure S12:** Principal component analysis of microbial communities. Data were centre log ratio transformed before principal component analysis using Euclidean distances. Prokaryote communities at peak drought (A) and recovery (C), and fungal communities at peak drought (B) and recovery (D). Shapes indicate the plant community (fast‐ or slow‐strategy), shape fill indicates the drought (or control) in year 2 (2021), and colour indicates the drought intensity in year 1 (control, mild drought, or severe drought in 2020). Scores of the principal component axes (PC1, PC2) of each community in the subsequent drought treatment (filled shapes) were used to explain variation in soil functioning (Figure 6).
**Table S1:** Prokaryote and fungal community composition. PERMANOVA (adonis2) showing differences in prokaryote and fungal community β‐diversity at peak drought and recovery. The interactions between soil legacies of increasing drought intensity (control, mild drought, or severe drought in year 1) and drought (or control) in year 2 that tended to affect community composition were further explored by dividing the dataset into the soil legacy groups (control, mild drought, or severe drought) and testing the effect of drought (compared to control) on prokaryote community composition.
**Table S2:** Variables associated with prokaryote and fungal community composition. Significance of constrained ordination (RDA) on prokaryote and fungal community β‐diversity at peak drought and recovery. Significance was tested using anova.cca() to determine the global model significance (‘Model’), the significance of each term (arrow) and the significance of each axis. Soil legacies of increasing drought intensity (soil water deficit, SWD in 2020), microbial biomass, dissolved organic nitrogen (DON), community aboveground productivity (aboveground biomass, g m^−2^) and soil N‐NO_3_^−^ (nitrate).
**Table S3:** Prokaryote and fungal community α‐diversity (Shannon Index). Prokaryote and fungal communities after 3 weeks of drought or control (peak drought) and 7 weeks after re‐wetting (recovery) in year 2. The significance of plant community (fast‐ or slow‐strategy plant community), soil legacies of increasing drought intensity (control, mild drought, or severe drought in year 1), drought (or control) in year 2, and all interactions were tested in linear models. Models were simplified to achieve the best‐fit parsimonious model, and significance was determined with ANOVA.
**Table S5:** Soil functioning in year 2 (multivariate relationships). (A) PERMANOVA (adonis2 output) showing the effect of soil legacies of increasing drought intensity (control, mild drought, or severe drought in 2020), the drought (or control) in year 2 and plant community (fast‐ or slow‐strategy) on soil functioning in year 2 at three sampling points: the spring before the drought, at peak drought and at recovery. The interactions between soil legacies of increasing drought intensity and plant community were further explored by dividing the dataset into the year 1 control, mild drought and severe drought treatments and testing the effect of plant strategy on soil functioning. (B) Significance of constrained ordination (RDA) on soil functioning in year 2 (Figure 6) in the spring before the drought, at peak drought and at recovery. Significance was tested using anova.cca() to determine the global model significance (‘Model’), the significance of each term (arrow) and the significance of each axis. Soil legacies of increasing drought intensity (SWD 2020), plant community aboveground biomass.
**Table S6:** Soil functioning in year 2 (univariate relationships). The effects of soil legacies of increasing drought intensity (control, mild drought, or severe drought in 2020), the year 2 drought (or control), plant community (fast‐ or slow‐strategy), and all 2‐way interactions on individual soil functions: soil ammonium (N‐NH_4_^+^), nitrate (N‐NO_3_^−^), microbial biomass carbon, and the potential enzyme activity of β‐glucosidase (pGLC), β‐N‐acetylglucosaminidase (pNAG), urease (pURE), peroxidase (pPER) and phenoloxidase (pPOX) at three timepoints in year 2: before the drought, at peak drought and at recovery. Significance was tested with linear models (N‐NH_4_^+^, N‐NO_3_^−^, microbial biomass) and linear mixed effects models (all potential enzyme activities; the experimental unit was used as the random factor to account for laboratory replicates). Significant differences between levels of a treatment are based on a Tukey post hoc test.
**Table S7:** The effect of soil legacies of increasing drought intensity (soil water deficit, SWD, in 2020) on the resistance and recovery of plant community aboveground biomass to drought in year 2 (2021). Baseline normalised resistance and recovery were calculated using the mean aboveground biomass of communities that were maintained at control conditions in both years as the continuous baseline (Ingrisch and Bahn 2018). Resistance was determined after 3 weeks of drought (peak drought) and recovery was determined 7 weeks after re‐wetting. Generalized additive models (GAMs) were used to determine significant relations between the soil water deficit in year 1 (SWD 2020) and resistance or recovery of fast‐ and slow‐strategy plant communities. The GAM model tested the effect of plant community (factor) and SWD 2020 (continuous) and their interaction on resistance or recovery in year 2. The significant interaction between SWD 2020 and plant community necessitated a second GAM, which tested the effect of SWD 2020 on resistance or recovery within each plant community. The Gamma (log = link) family was chosen to fit the GAMs as this resulted in the best fit.
**Table S8:** Soil community structure and function relationships with plant resistance and recovery. Relationships between measured soil variables and year 2 drought resistance and recovery of aboveground net primary productivity (ANPP) to the subsequent drought. Only resistance of plant communities that were destructively harvested at peak drought are included (i.e., in the experimental units where soil parameters were measured, *n* = 28). Relationships were determined with GAM models to first establish if there was an interaction between the plant community and the measured variable, and then the relationships within each plant community were tested. The Gamma(log = link) family was chosen to fit the GAMs, as this resulted in the best fit.

**Table S4:** The relative abundance of prokaryote and fungal phyla and families. Soil legacies of increasing drought intensity (control, mild drought, or severe drought in year 1), drought (or control) in year 2, plant community (fast‐ or slow‐strategy), and their interactions on the relative abundance of prokaryote and fungal phyla, and the 20 most abundant prokaryote and fungal families (excluding families that were ‘unknown’). We tested the effects of soil legacies, drought, plant community and their interactions at peak drought year 2 (after 4 weeks of drought) and at recovery (7 weeks after re‐wetting). We used linear models and ANOVA with a Benjamini‐Hochberg adjustment (Benjamini and Hochberg 1995) using the R function *p*.adjust to correct *p* values for multiple comparisons.

## Data Availability

Amplicon sequencing data has been deposited in the NCBI database under BioProject numbers PRJNA1308790 (ITS sequences) and PRJNA1307182 (16S rRNA sequences). All data are available on figshare (https://doi.org/10.6084/m9.figshare.29958197, https://doi.org/10.6084/m9.figshare.29958161) as well as the R code to reproduce the figures (https://doi.org/10.6084/m9.figshare.29958134).
