# Supplementary material for: Drought Intensity Shapes Soil Legacy Effects on Grassland Plant and Soil Microbial Communities and Their Responses to Future Drought
Source: Glob Chang Biol. 2025 Sep 18;31(9):e70495. doi: 10.1111/gcb.70495 (PMC12445341; doi:10.1111/gcb.70495)
Supplement: Supplementary file 1 — Figure S1: The soil water deficit (SWD, % of field capacity) during the drought period in 2020 (year 1), which is referred to in this study as soil legacies of increasing drought intensity. This figure is originally published in Oram et al. (2023) and reprinted in Oram et al. (2025). (A) Realised SWD indicates the measured SWD over time (day of the calendar year). The grey horizontal line at 20% realised SWD indicates the target control, the colours indicate the target SWD. (B) The relationship between realised and target SWD at peak drought (225 days of the calendar year). The diagonal dashed line indicates the 1:1 line, the solid black line is a linear regression and the R 2 indicates the adjusted R 2 of the linear regression between realised and target SWD. Figure S2: Climatic conditions throughout the experimental period in 2021. (A) Daily precipitation, (B) air temperature and (C) vapour pressure deficit, (Year‐Month‐Day). Data were obtained from GeoSphere Austria (https://data.hub.geosphere.at). Figure S3: Dry‐down dynamics in 2021 for the fast‐ and the slow‐strategy communities in the control and the drought treatment during the 3‐week drought period. Soil water deficit (SWD) indicates the percent deficit from field capacity. Points are jittered for visualisation. Figure S4: Sequencing depth. Rarefaction curves of prokaryote (A, B) and fungal (C, D) taxa based on sequence reads in soil samples taken after 21 days of drought (peak drought) and 7 weeks after re‐wetting (recovery). Species refers to the number of taxa; sample size indicates the number of reads. Each line represents one experimental unit/sample, indicated by the number in the square. Figure S5: Prokaryote and fungal α‐diversity (Shannon Index). The effect of soil legacies of increasing drought intensity (control, mild drought, or severe drought in year 1), plant community (fast‐ or slow‐strategy), and drought (or control) in year 2 on the Shannon Index of prokaryote (A) or fungal (B) communities [file GCB-31-e70495-s002.docx]

# Title: Drought intensity shapes soil legacy effects on grassland plant and soil microbial communities and their responses to future drought

**Running Title:** Drought intensity shapes soil drought legacies

**Author List:**

Natalie J. Oram^1,2,3*^, Nadine Praeg^4^, Richard D. Bardgett^5,6^, Fiona Brennan^2^, Tancredi Caruso^7^, Paul Illmer^4^, Johannes Ingrisch^1^, Michael Bahn^1^

**Author ORCID #:**

Natalie J. Oram ([0000-0002-3529-5166](https://orcid.org/0000-0002-3529-5166))
Nadine Praeg ([0000-0002-1531-8543](https://orcid.org/0000-0002-1531-8543))
Richard D. Bardgett ([0000-0002-5131-0127](https://orcid.org/0000-0002-5131-0127))
Fiona Brennan ([0000-0002-2949-6180](https://orcid.org/0000-0002-2949-6180))
Tancredi Caruso ([0000-0002-3607-9609](https://orcid.org/0000-0002-3607-9609))
Paul Illmer ([0000-0003-3368-3015](https://orcid.org/0000-0003-3368-3015))
Johannes Ingrisch ([0000-0002-8461-8689](https://orcid.org/0000-0002-8461-8689))
Michael Bahn ([0000-0001-7482-9776](https://orcid.org/0000-0001-7482-9776))

**Institutional affiliations:**

^1^ Department of Ecology, Universität Innsbruck, Innsbruck, Austria

^2^ Environment, Soils and Land Use Department, Teagasc, Johnstown Castle, Ireland

^3^ Institute for Biodiversity and Ecosystem Dynamics, University of Amsterdam, Amsterdam, The Netherlands (current affiliation)

^4^ Department of Microbiology, Universität Innsbruck, Innsbruck, Austria

^5^ Department of Earth and Environmental Sciences, The University of Manchester, Manchester, UK

^6^Lancaster Environment Centre, Lancaster University, Lancaster LA1 4YQ, UK.

^7^ School of Biology & Environmental Science, University College Dublin, Dublin 4, Belfield, Ireland

**Corresponding Author Contact Information:**

[n.j.oram@uva.nl](mailto:n.j.oram@uva.nl), +31 6 17723885

**Supplementary Methods**

*Soil functioning – potential extracellular enzyme activity*

We measured pEEA of three hydrolytic and two oxidative enzymes involved in C and N cycling: β-glucosidase (pGLC), β-N-acetylglucosaminidase (pNAG), urease (pURE), peroxidase (pPER), and phenoloxidase (pPOX) as indicators of microbial community functioning. We optimized the assays for our soil (other than pPER and pPOX as lignin oxidases do not follow enzyme kinetics) by testing activity over a range of substrate concentrations to determine the minimum substrate concentration that causes maximum potential enzyme activity, which was then used in the assays.

The potential activity of pGLC and pNAG were determined according to (Jackson et al., 2013). Briefly, we stirred 3.75 g fresh soil and 5 mL 50 mM sodium acetate buffer (pH 6.0) on a magnetic stirring plate (IKA RT 10, Germany) for 15 minutes before pipetting 150 µL of the slurry into a deep well plate. Nitrophenol linked substrates (20 mM pNP- β-D-glucopyranoside for pGLC and 2.5 mM 4-Nitrophenyl β-N-acetylglucosaminide for pNAG) were added, respectively, and then the plates were incubated under continuous shaking for 1.5 hours (β-glucosidase) or 3.5 hours (β-N-acetylglucosaminidase) on a mixing block MB-102 (Bioer, China) at 20°C. After incubation, plates were centrifuged (10 minutes 1218 xg) to pellet soil. 100 µL of the supernatant and *p*-nitrophenol standards were mixed with 200 µL of 50 mM NaOH in a 96-well reading plate. Absorbance at 405 nm was read on a Zenyth 3100 plate reader (Anthos Labtec Instruments, Austria). Each soil sample was measured in four analytical replicates.

pURE was determined according to (Cordero et al., 2019). Briefly, we stirred 2.0 g fresh soil with 5 mL 50 mM sodium acetate buffer (pH 6.0) on a magnetic stirring plate (IKA RT 10, Germany) for 15 minutes before pipetting 250 µL of the slurry into a deep well plate (six analytical replicates per sample). We added 100 µL 60 mM urea solution to four of the analytical replicates, leaving two as blanks (adding 50 mM sodium acetate buffer instead). Plates were sealed, vortexed, and incubated under continuous shaking on a mixing block (Bioer, China) for two hours at 20⁰C. After incubation, 1 mL 2M KCl was added, plates were re-sealed, vortexed, and incubated with continuous shaking for a further 30 minutes. We centrifuged the plates (10 minutes, 1218 xg) and transferred 75 µL of the supernatant into a reading plate. Supernatants and ammonium calibration curve (eight concentrations from 0 to 4.5 mg L^-1^ N-NH_4_^+^) were mixed with 75 µL MiliQ water, 75 µL colour reagent (sodium salicylate, sodium nitroprusside dihydrate and NaOH) and 75 µL oxidative solution (dichloroisocyanuric acid sodium salt dihydrate). Absorbance at 650 nm after 30 minutes was read using a microplate reader (Tecan Sunrise Remote, Austria).

Potential activity of lignin oxidases (pPER and pPOX) was determined according to (Sinsabaugh & Linkins, 1988). Briefly, we stirred 0.25 g of soil and 25 mL 50 mM sodium acetate buffer (pH 5.0) for 15 minutes, pipetted 0.4 mL into a deep well plate and added 0.4 mL 20 mM L-3,4-dihydroxyphenylalanin (DOPA). The plate was incubated for 10 minutes with continuous shaking (mixing block MB-102, Bioer, China), centrifuged (10 minutes 1218g) and 250 µL of the supernatant was transferred to a 96-well reading plate (six analytical replicates/sample). To three of the analytical replicates, we added 10 µL of 0.3% H_2_O_2_ to determine peroxidase activity. We measured absorbance at 450 nm immediately (start point), after 1.5 hours (end point for pPER), and after 20 hours (end point for pPOX) all on a Zenyth 3100 plate reader (Anthos Labtec Instruments, Austria).

### *Microbial community composition – amplicon sequencing*

We identified prokaryotes (bacteria and archaea) and fungi, by high-throughput amplicon sequencing the marker genes 16S rRNA and ITS, respectively, on Illumina MiSeq (ITS) and NextSeq (16S rRNA) platforms. DNA was extracted from frozen soil (fresh soil frozen at sampling at -80℃) without thawing using the DNeasy PowerSoil Pro Kit (Qiagen) following manufacturer’s instructions. Sequencing library preparation involved two PCR steps. The first PCR reactions contained 2.5 µL template DNA (5 ng µL^-1^), 12.5 µL KAPA HiFi HotStart ReadyMix (Roche, Ireland), and 0.5 µL 1µM forward and reverse adapted primers (Eurofins, UK). For 16S rRNA, we used primer pair 515F and 926R (GTGYCAGCMGCCGCGGTAA / CCGYCAATTYMTTTRAGTTT) (Walters et al., 2016). For ITS we targeted ITS2 (ITS86F) – ITS4 (TGAATCATCGAATCTTTGAA/ TCCTCCGCTTATTGATATGC) (Vancov & Keen, 2009). PCR conditions for 16S rRNA were: 3 minutes at 94⁰C, 25 cycles of: 45 seconds at 94⁰C, 45 seconds at 50⁰C, and 60 seconds at 72⁰C, and finally five minutes at 72⁰C. PCR conditions for ITS were: 3 minutes at 95⁰C, then 25 cycles of: 30 seconds at 95⁰C, 30 seconds at 54⁰C, and 30 seconds at 72⁰C, and finally five minutes at 72⁰C. Because ITS DNA yields are lower, we ran the first PCR in triplicate (i.e., three laboratory replicates for each experimental unit) and pooled 3 PCR products at this stage. Amplicons were purified with Ampure XP beads (Beckmann Coulter, UK) according to the manufacturer’s instructions, and eluted in 15 µL TE buffer. In a second PCR, Illumina sequencing adaptors were added on 16S rRNA and ITS amplicons using Nextera XT kit in a 50 µL reaction: 5 µL PCR 1 product, 5 µL of each Nextera index primer, 25 µL KAPA HiFi HotStart ReadyMix, and 10 µL nucleic acid free water. The second PCR conditions were: 3 minutes at 95⁰C, then 7 cycles of: 30 seconds at 95⁰C, 30 seconds at 55⁰C, 30 seconds at 72⁰C, and finally 5 mins at 72⁰C. Amplicons were again purified using Ampure XP beads (Beckmann Coulter, UK) according to manufacturer’s instructions, and eluted in 15 µL TE buffer as above. The concentration of each PCR2 product was quantified using the dsDNA HS Assay Kit, Qubit (Thermofisher, Ireland), and the products were pooled in equimolar concentration before sequencing using Illumina MiSeq platform (ITS) or Illumina NexSeq platform (16S rRNA), resulting in paired end 300 base pair reads. Raw sequencing data were deposited in the NCBI Sequence Read Archive (SRA) and are accessible under the BioProject numbers PRJNA1308790 (ITS sequences) and PRJNA1307182 (16S sequences).

**Supplementary Figures**


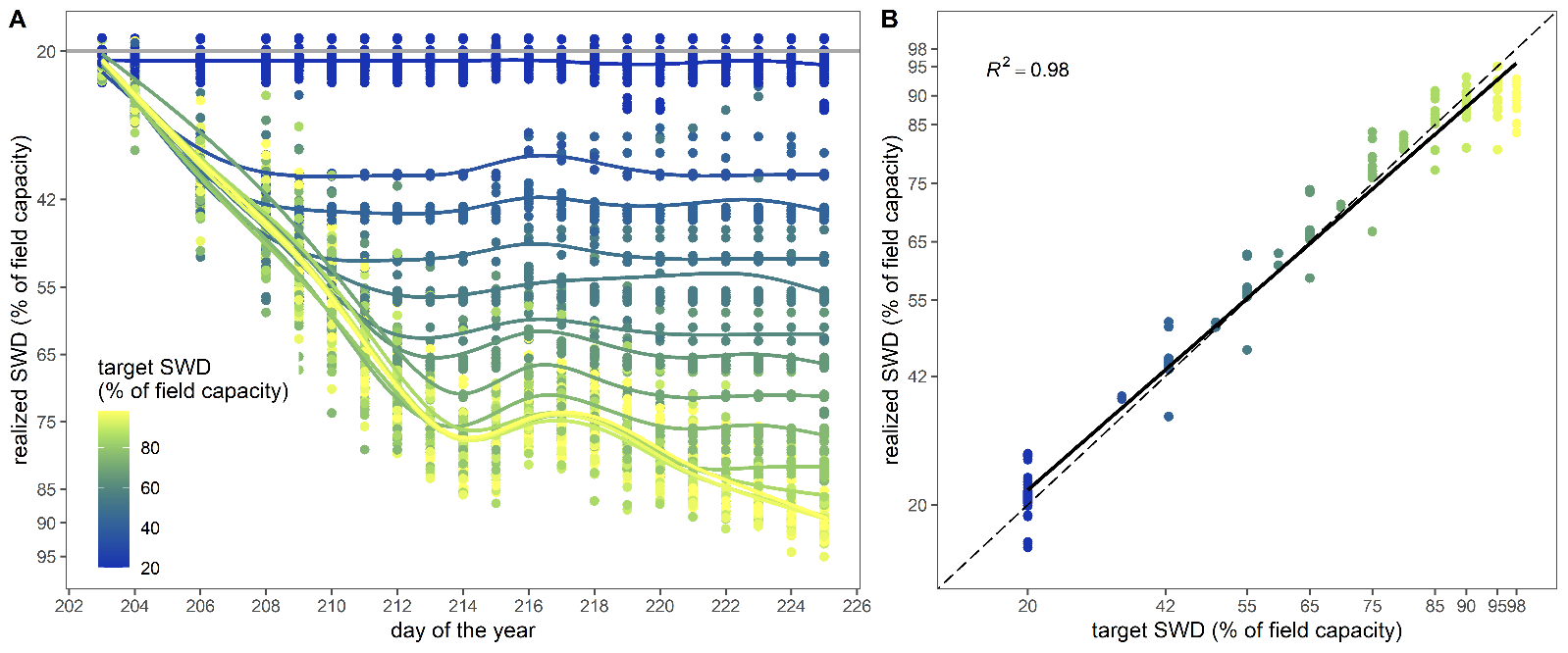


Fig. S1. The soil water deficit (SWD, % of field capacity) during the drought period in 2020 (year 1), which is referred to in this study as soil legacies of increasing drought intensity. This figure is originally published in Oram et al., (2023) and re-printed in Oram et al., (2025). (A) Realized SWD indicates the measured SWD over time (day of the calendar year). The grey horizontal line at 20% realized SWD indicates the target control, the colours indicate the target SWD. (B) The relationship between realized and target SWD at peak drought (225 day of the calendar year). The diagonal dashed line indicates the 1:1 line, the solid black line is a linear regression, the R^2^ indicates the adjusted R^2^ of the linear regression between realized and target SWD.

**
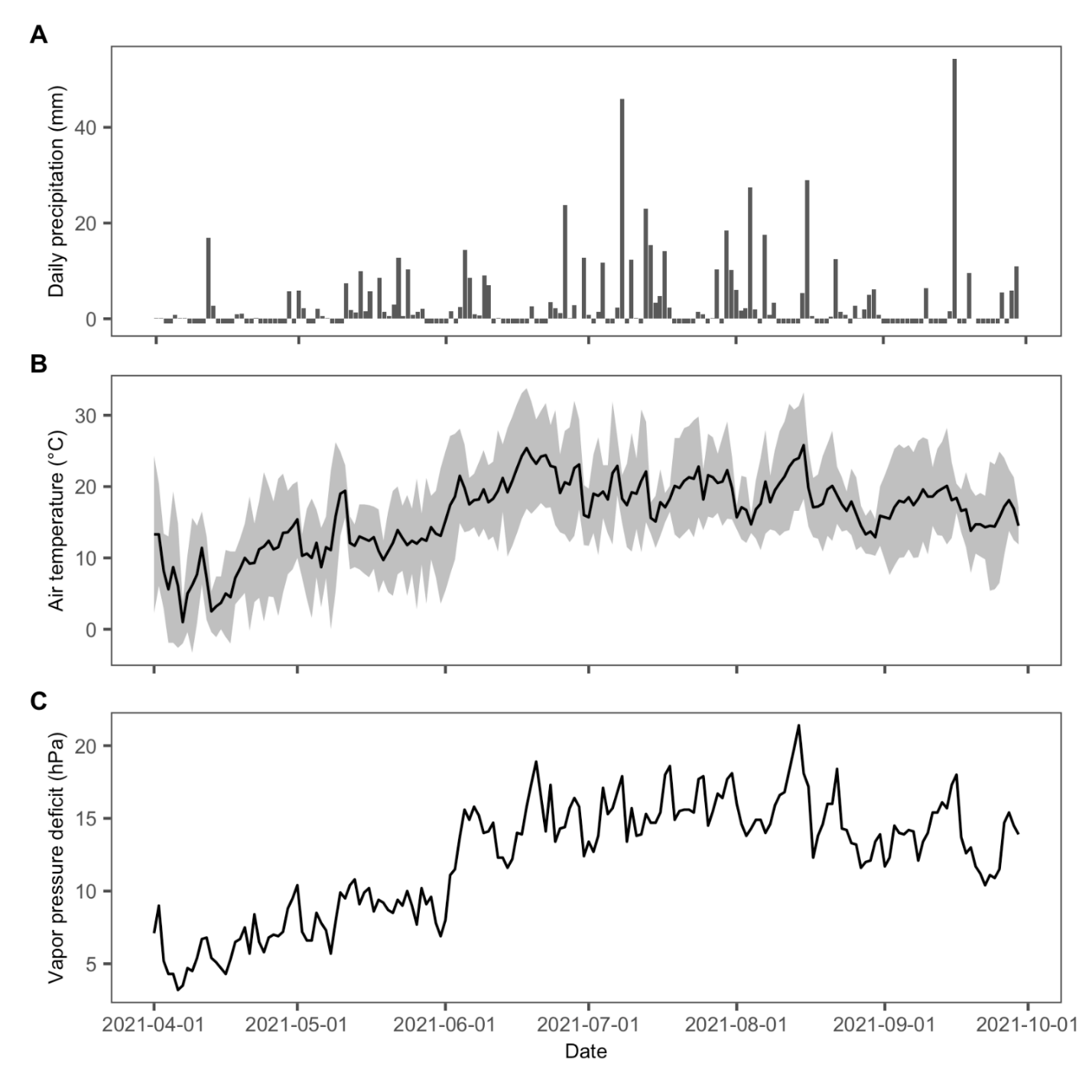
**Fig. S2. Climatic conditions throughout the experimental period in 2021. (A) Daily precipitation, (B) air temperature and (C) vapor pressure deficit, (Year-Month-Day). Data were obtained from GeoSphere Austria (<https://data.hub.geosphere.at>).


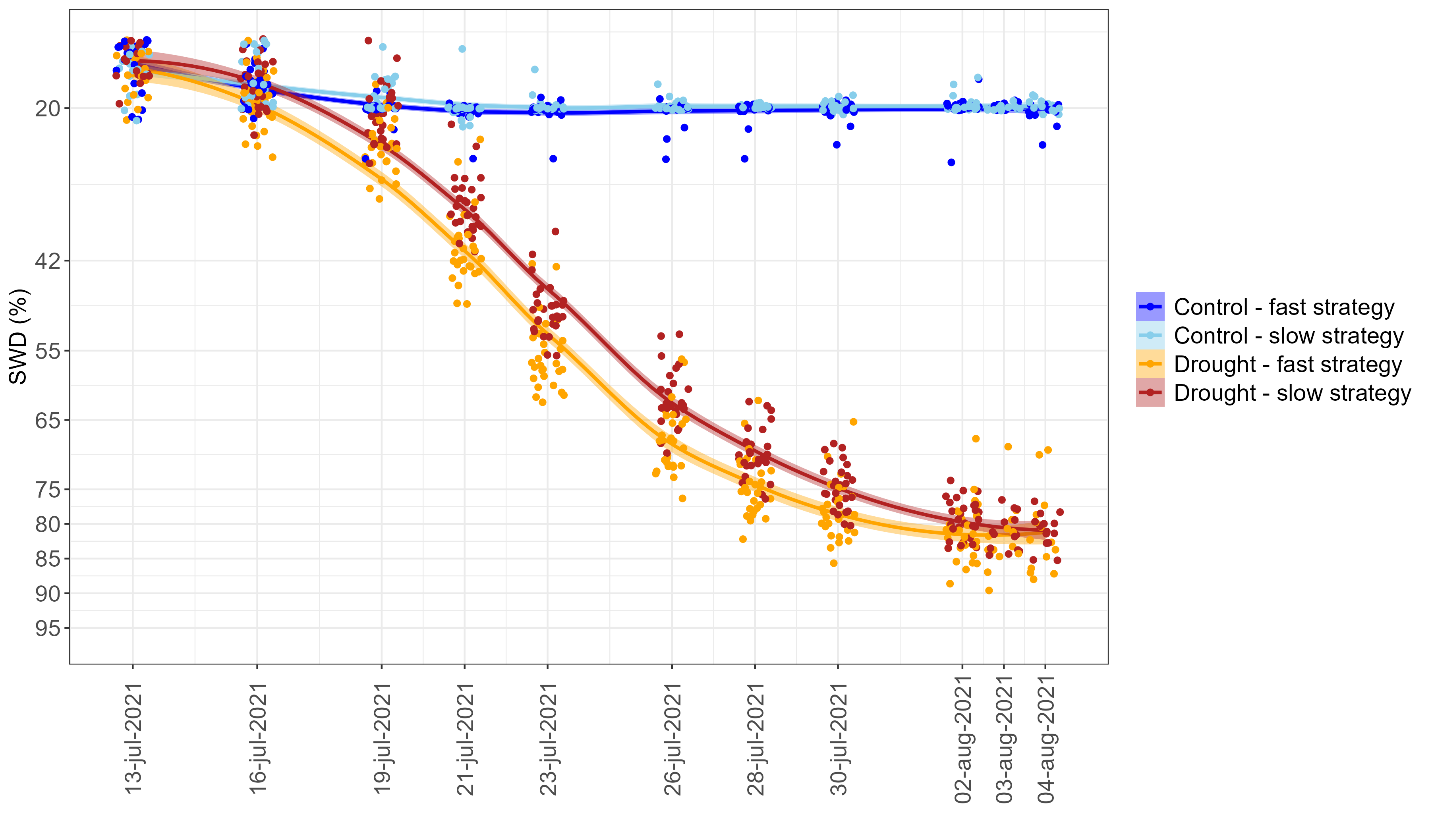


Fig. S3. Dry down dynamics in 2021 for the fast- and the slow-strategy communities in the control and the drought treatment during the three-week drought period. Soil water deficit (SWD) indicates the percent deficit from field capacity. Points are jittered for visualisation.

**
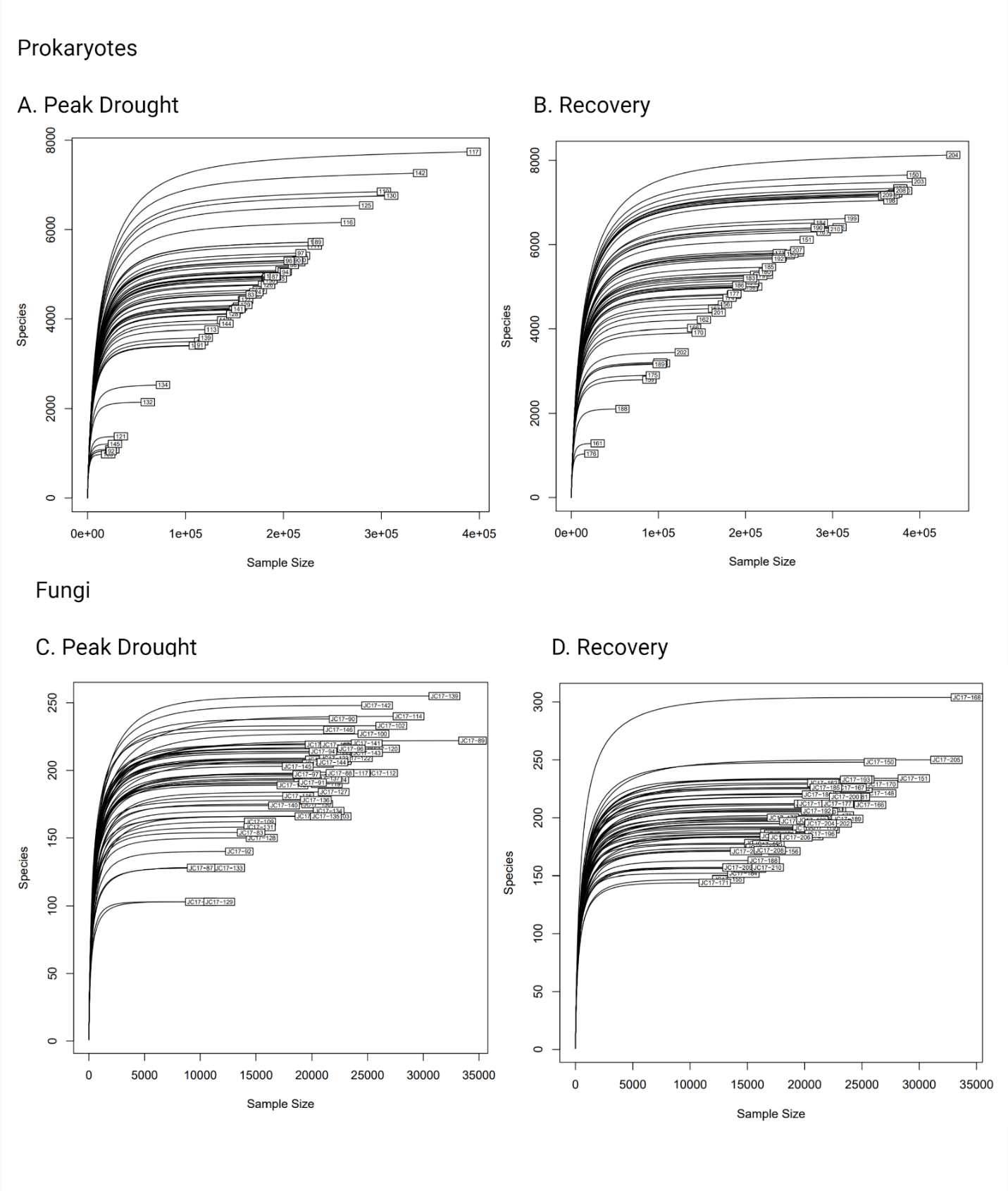
**

Fig. S4. Sequencing depth. Rarefaction curves of prokaryote (A, B) and fungal (C, D) taxa based on sequence reads in soil samples taken after 21 days of drought (peak drought) and seven weeks after rewetting (recovery). Species refer to the number of taxa; sample size indicates the number of reads. Each line represents one experimental unit/sample indicated by the number in the square.


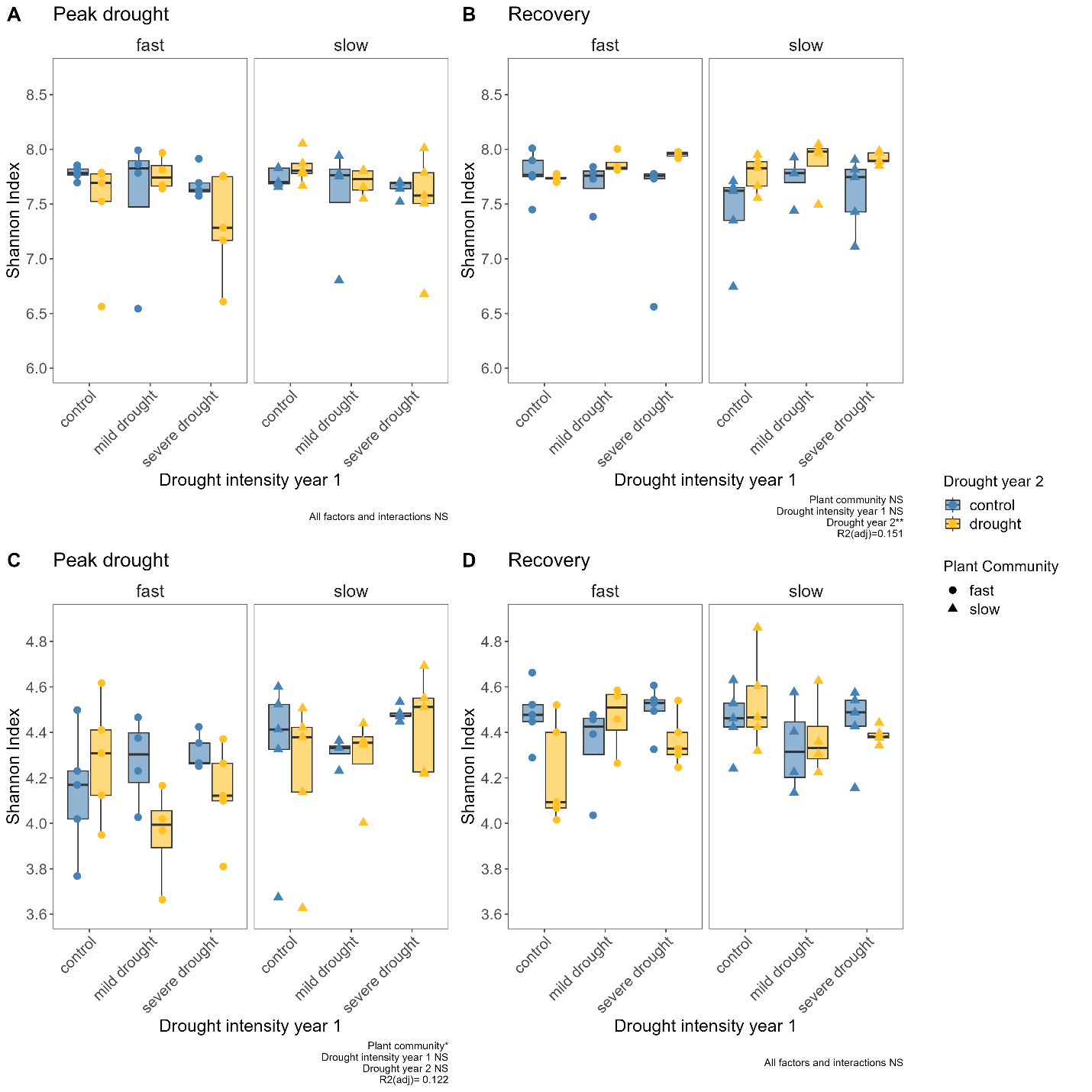


Fig. S5. Prokaryote and fungal α-diversity (Shannon Index). The effect of soil legacies of increasing drought intensity (control, mild drought, or severe drought in year 1), plant community (fast- or slow-strategy), and drought (or control) in year 2 on the Shannon Index of prokaryote (A) or (B) fungal communities at peak drought in year 2 and the prokaryote (C) or fungal (D) communities seven weeks after re-wetting. Captions indicate significance based on ANOVA (see Table S3 for complete statistical output).

**
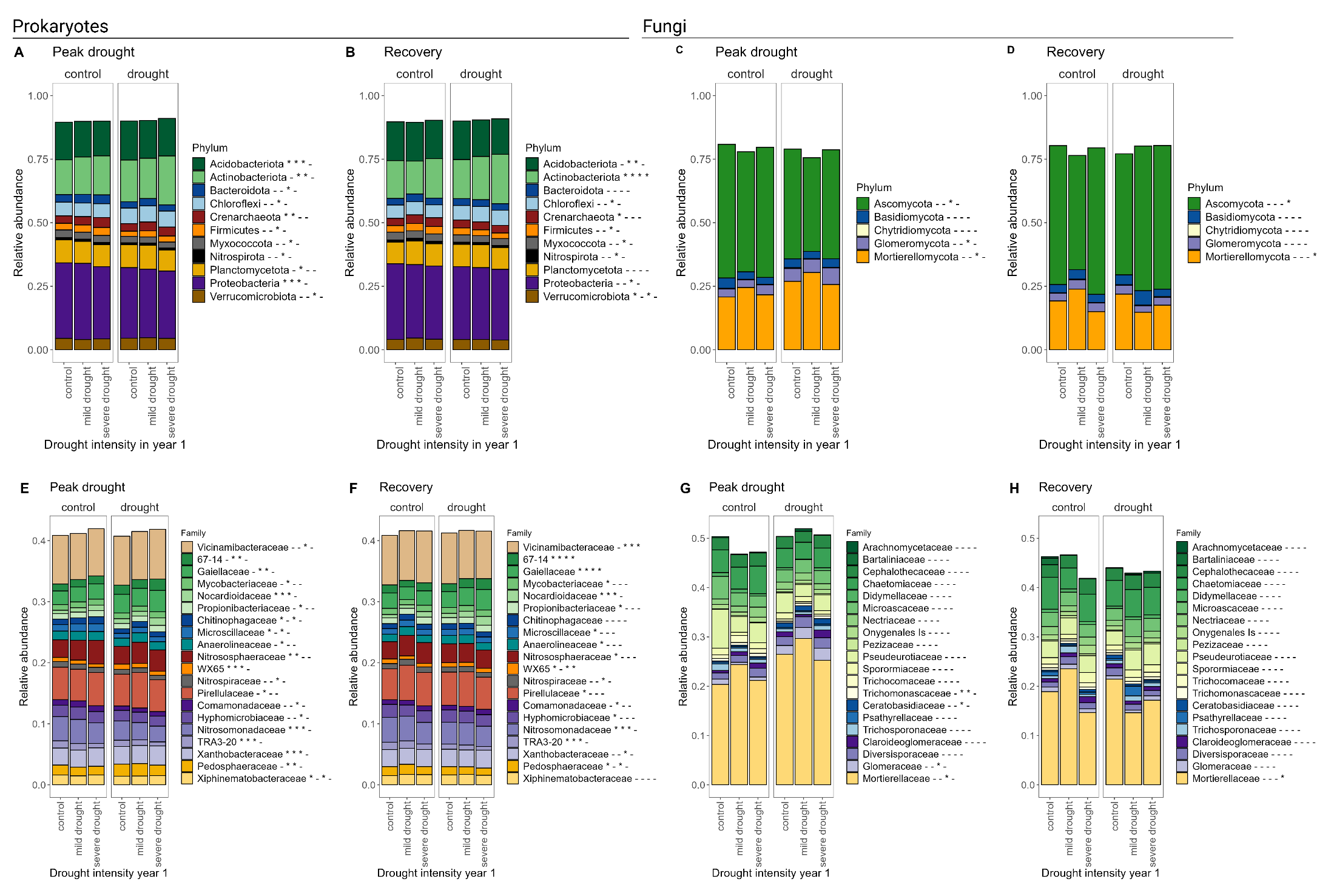
**

Fig. S6. The relative abundance of prokaryote and fungal phyla and families. Soil legacies of increasing drought intensity (control, mild drought, or severe drought in year 1), drought or control in year 2, and their interactive effects on the relative abundance of prokaryote and fungal phyla (A-D) and the 20 most abundant families, excluding ‘unknown’ families (E-H) at the peak drought and recovery timepoints in year 2. Shades of the same colour indicate that the family belongs to the same phylum. Significance was tested using linear models and anova (see Methods). Symbols beside the family name in the legend denote significance of experimental treatments in the following order: Soil legacies of increasing drought intensity in control conditions in year 2, Soil legacies of increasing drought intensity in drought conditions in year 2, the year 2 drought versus control, and the interaction between soil legacies and the year 2 drought or control (P < 0.05 *, P > 0.05 -), see Table S4 for statistical output.


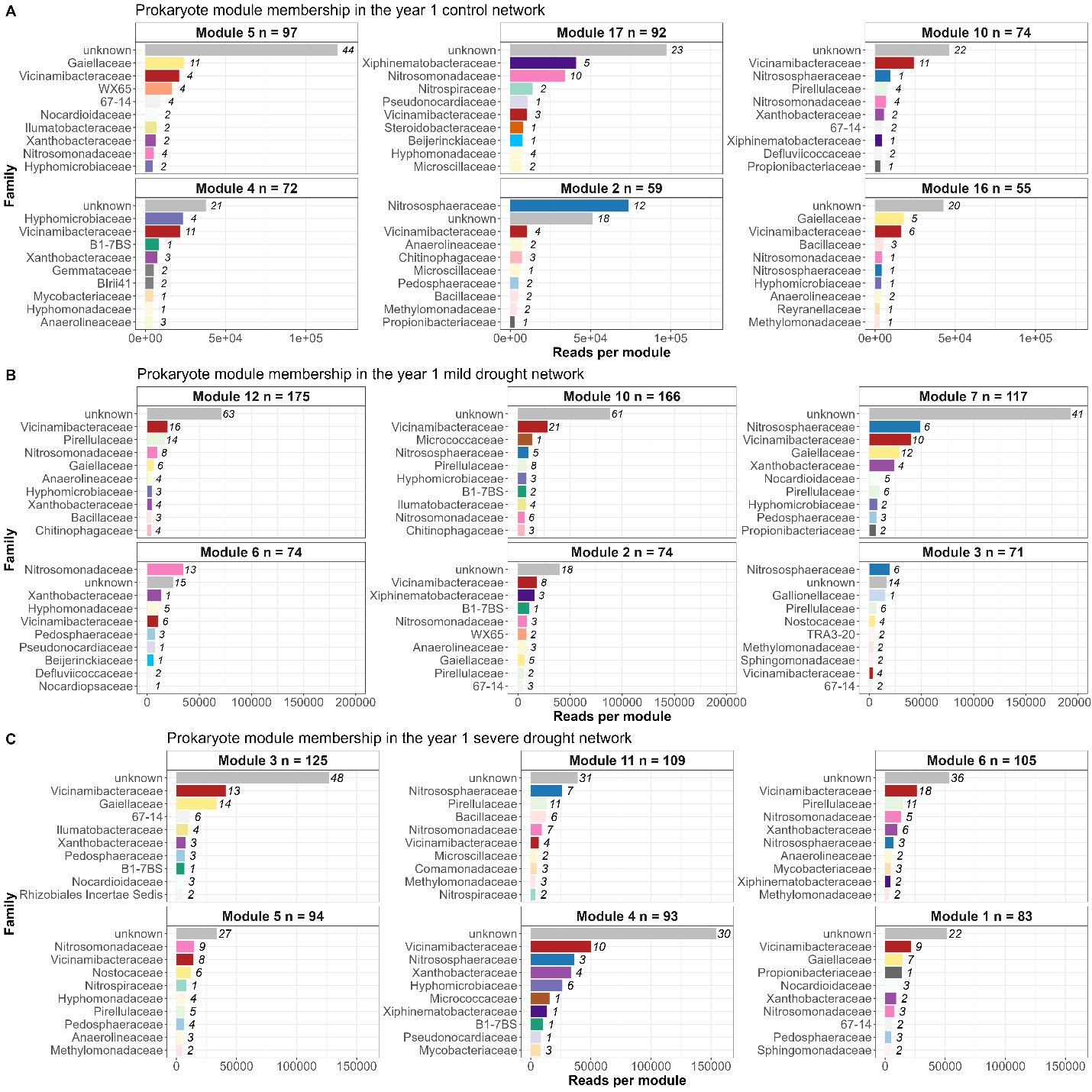


Fig S7. Prokaryote network module membership at peak drought year 2. Prokaryote module membership in the largest six modules at the peak drought timepoint in year 2 in the networks exposed to (A) a control, (B) mild drought, and (C) severe drought in year 1. The colour indicates the family (also indicated on the Y axis), the number beside the bar denotes the number of ASVs within each family. The title of each panel indicates the module number and the number of nodes (ASVs) included in that module. For aesthetic clarity, only the 10 most abundant families (based on read number) are shown.


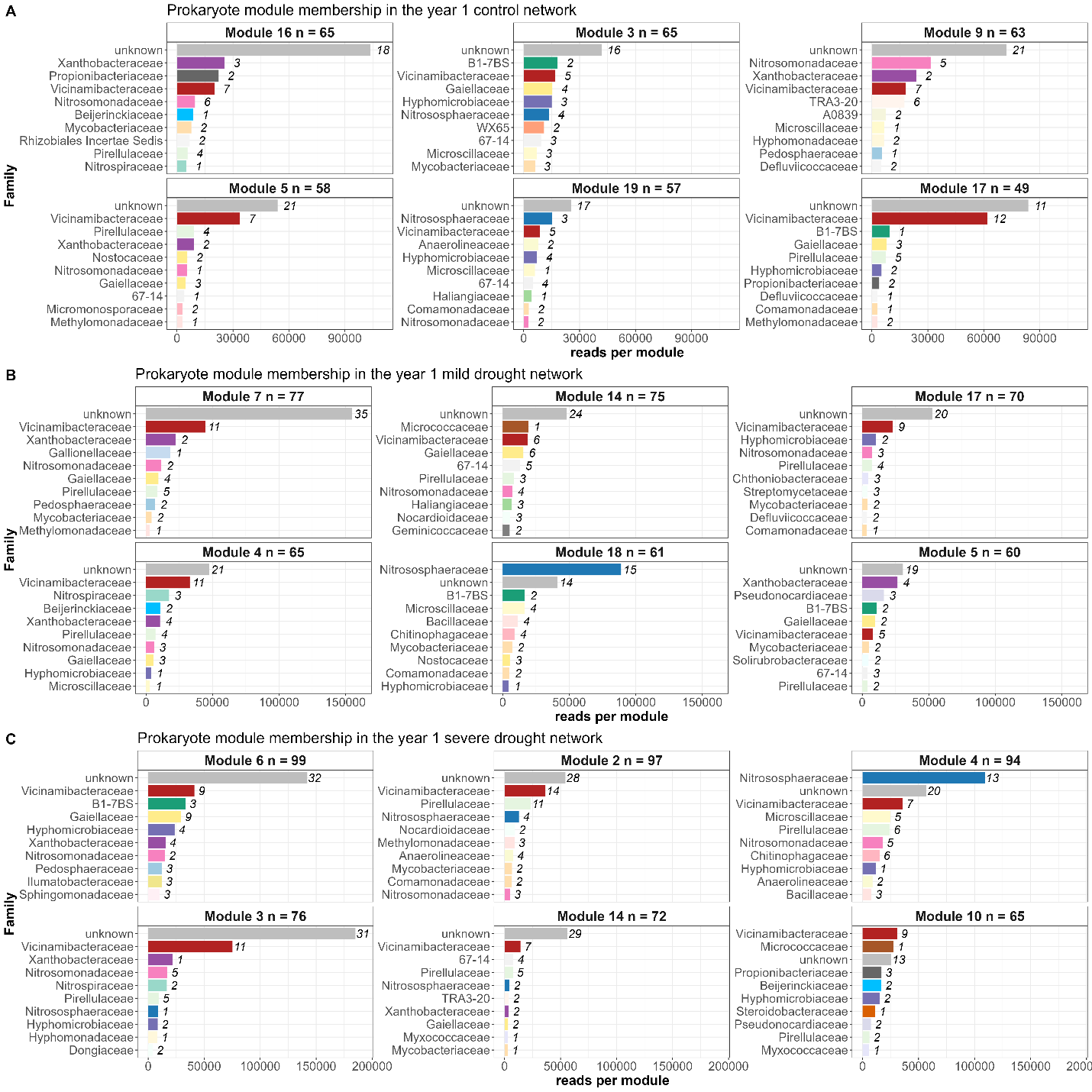


Fig. S8. Prokaryote network module membership at recovery year 2**.** Prokaryote module membership in the largest six modules at the recovery timepoint in year 2 in the networks exposed to (A) a control, (B) mild drought, and (C) severe drought in year 1. The colour indicates the family (also indicated on the Y axis), the number beside the bar denotes the number of ASVs within each family. The title of each panel indicates the module number corresponding to that in Fig. 2 D-F, along with the number of nodes (ASVs) included in that module. For aesthetic clarity, only the 10 most abundant families (based on read number) are shown in the figure.


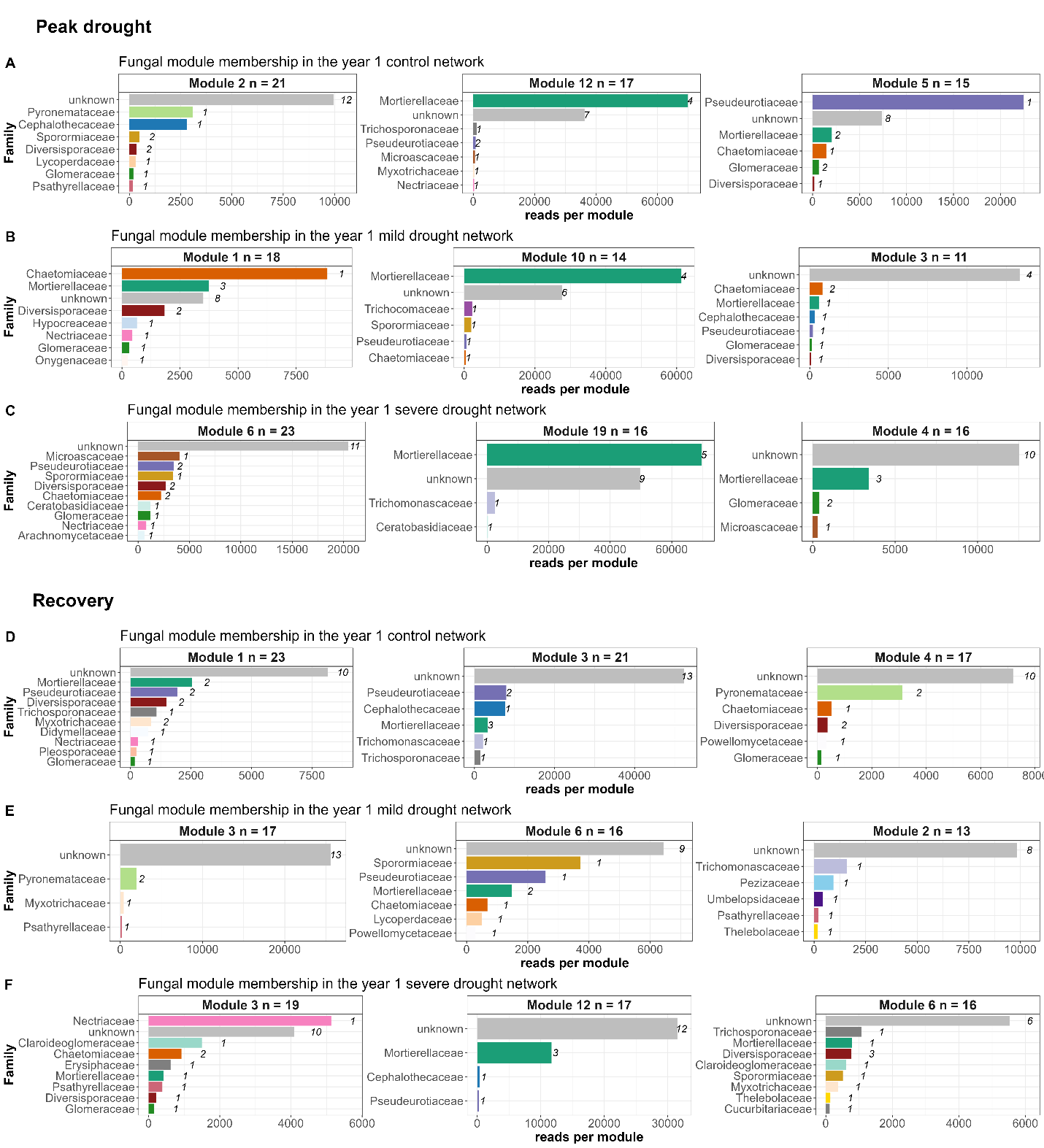


Fig S9. Fungal network module membership in year 2. Fungal module membership in the largest three modules per network at the (A-C) peak drought and (D-F) recovery timepoint in year 2, in fungal networks in soil that experienced either a control, mild drought, or severe drought in year 1. The colour indicates the family (also indicated on the Y axis), the number beside the bar denotes the number of ASVs within each family. The title of each panel indicates the module number and the number of nodes (ASVs) included in that module. All families with membership in a module are shown.

**
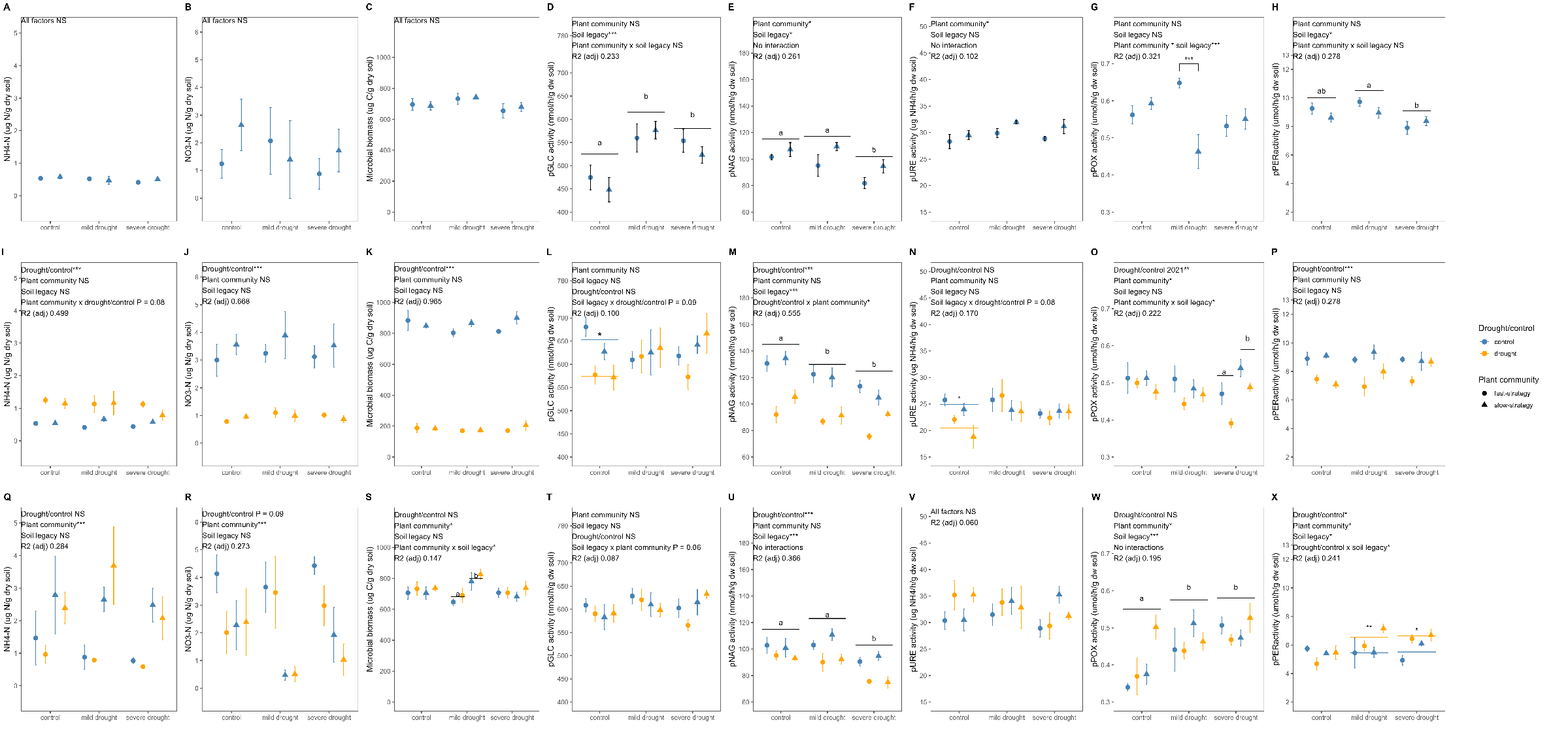
**

Fig. S10. Soil functioning in year 2 (univariate relations). Soil ammonium (N-NH4), nitrate (N-NO3), microbial biomass, and the potential enzyme activity of β-glucosidase (pGLC), β-N-acetylglucosaminidase (pNAG), urease (pURE), peroxidase (pPER), and phenoloxidase (pPOX) before the subsequent drought (A-H), at peak drought (I-P), and at recovery (Q-X). Effects of the drought intensity in year 1 (called here soil legacy for brevity: control, mild drought, or severe drought in 2020), the drought (or control) in year 2 (blue or yellow points), plant community (fast- or slow-strategy) and all 2-way interactions were tested with linear models (N-NH_4_, N-NO_3_, microbial biomass) and linear mixed effects models (all potential enzyme activities to account for laboratory replicates). Letters indicate significant differences between levels of a treatment based on a Tukey post-hoc test. P < 0.05*, P < 0.01**, P < 0.001***


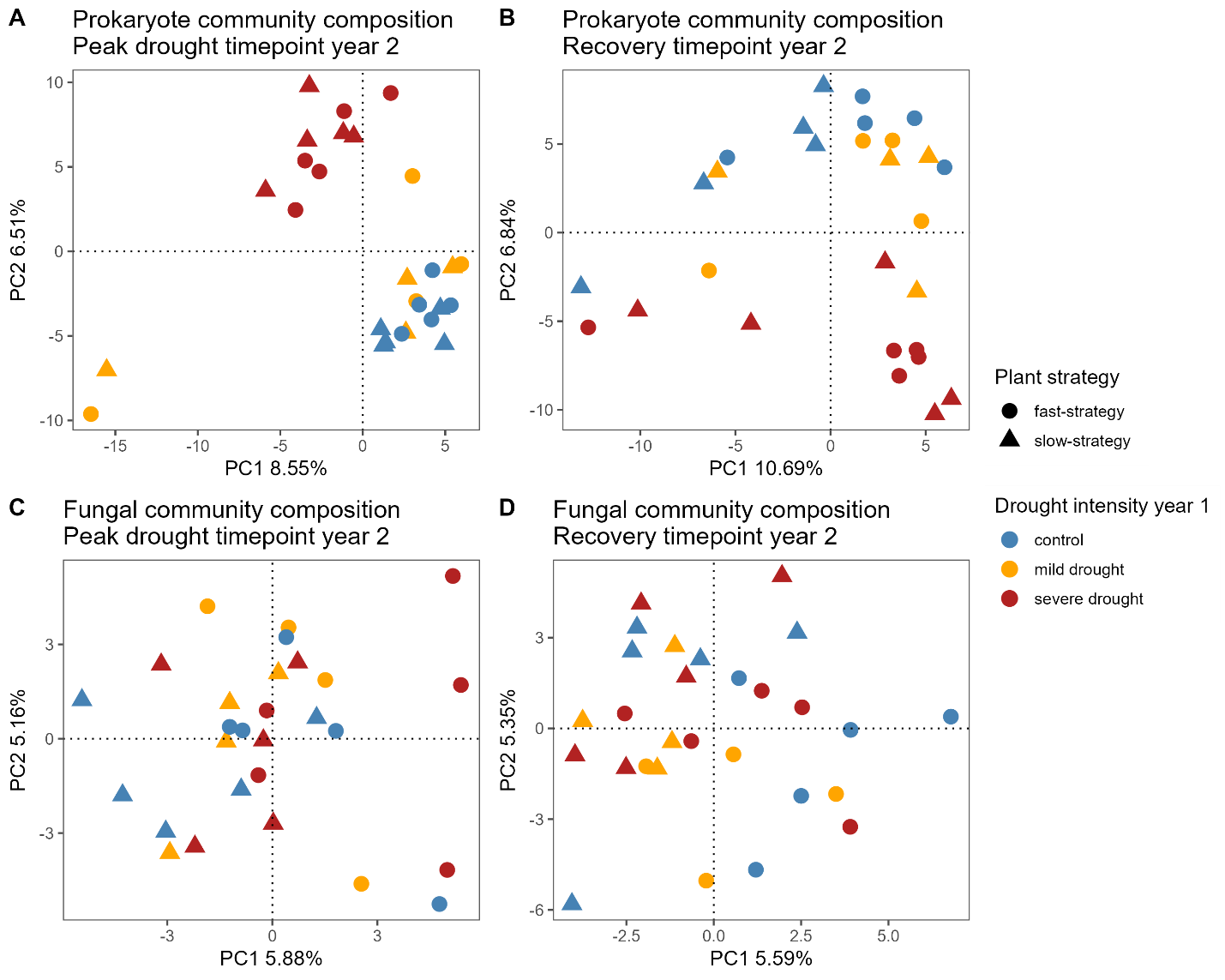


Fig. S11. Principal component analysis of microbial communities in the control treatment in year 2 at the ‘peak drought’ and ‘recovery’ timepoints. Note that these communities were not exposed to the year 2 drought but were maintained at control soil moisture throughout year 2. Read numbers were centre log ratio transformed before principal component analysis using Euclidean distances. Shapes indicate the plant community (fast- or slow-strategy) and colour indicates the soil drought legacy (control, mild drought, or severe drought in year 1). Scores of the principal component axes (PC1, PC2) of prokaryote and fungal communities were used to explain the resistance and recovery of plant community aboveground biomass to the drought in year 2 (see Fig. 8 in the main text).


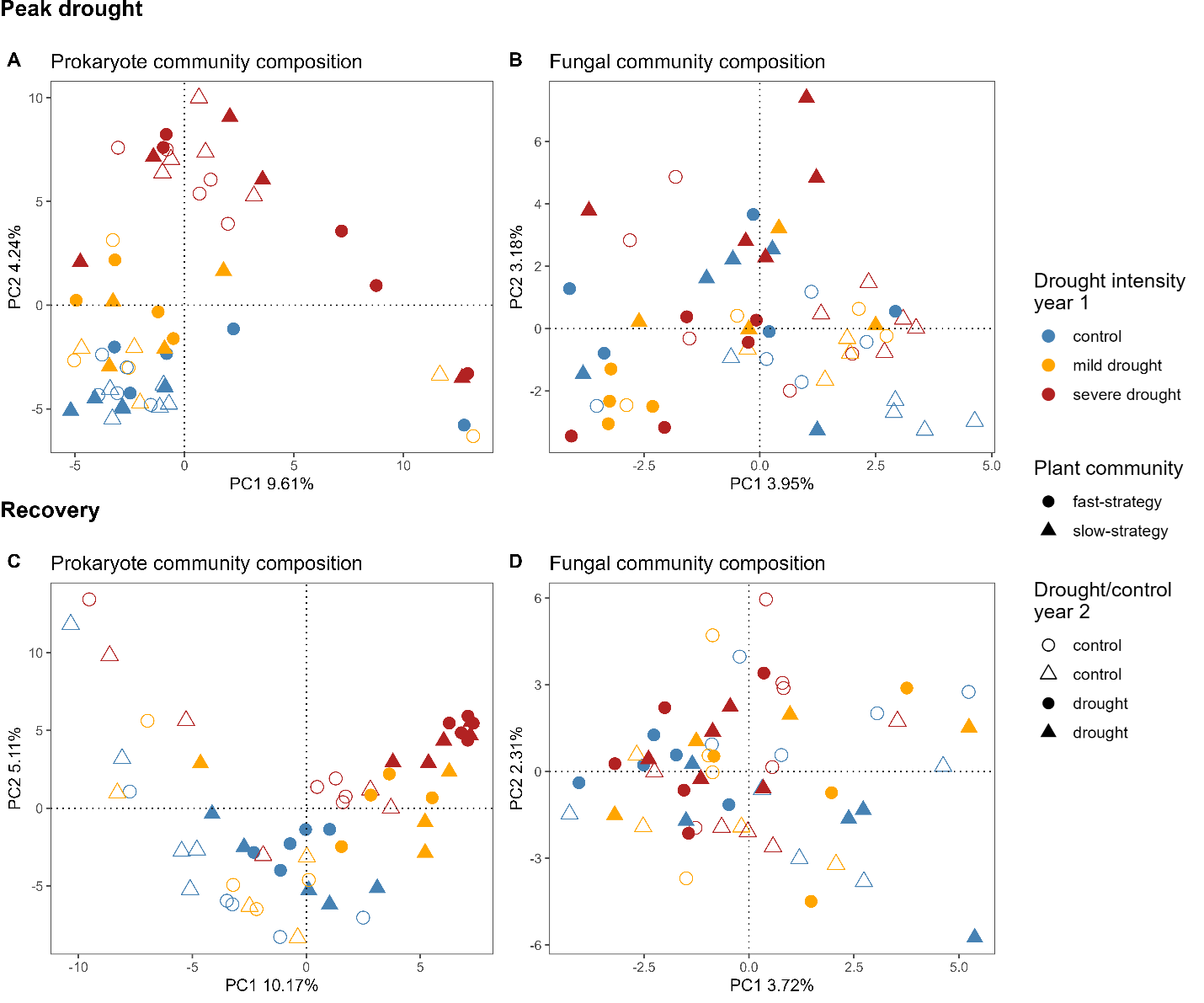


Fig. S12. Principal component analysis of microbial communities. Data were centre log ratio transformed before principal component analysis using Euclidean distances. Prokaryote communities at peak drought (A) and recovery (C), fungal communities at peak drought (B), and recovery (D). Shapes indicate the plant community (fast- or slow-strategy), shape fill indicates the drought (or control) in year 2 (2021), and colour indicates the drought intensity in year 1 (control, mild drought, or severe drought in 2020). Scores of the principal component axes (PC1, PC2) of each community in the subsequent drought treatment (filled shapes) were used to explain variation in soil functioning (Fig. 6).

Table S1. Prokaryote and fungal community composition. PERMANOVA (adonis2) showing differences in prokaryote and fungal community β-diversity at peak drought and recovery. The interactions between soil legacies of increasing drought intensity (control, mild drought or severe drought in year 1) and drought (or control) in year 2 that tended to affect community composition were further explored by dividing the dataset into the soil legacy groups (control, mild drought, or severe drought) and testing the effect of drought (compared to control) on prokaryote community composition.

| **Prokaryote community composition** | | |  |  |  |
| --- | --- | --- | --- | --- | --- |
| ***Peak Drought*** | *df* | *SumOfSqs* | *R2* | *statistic* | *p.value* |
| Drought year 2 | 1 | 54158.78 | 0.032 | 1.85 | 0.002 |
| Plant community | 1 | 30219.96 | 0.018 | 1.03 | 0.28 |
| Drought intensity year 1 | 2 | 103295.01 | 0.061 | 1.76 | 0.001 |
| Drought year 2 x Plant community | 1 | 31874.69 | 0.019 | 1.09 | 0.189 |
| Drought year 2 x Drought intensity year 1 | 2 | 69109.56 | 0.041 | 1.18 | 0.056 |
| Plant community x Drought intensity year 1 | 2 | 54190.55 | 0.032 | 0.93 | 0.789 |
| Residual | 46 | 1346813.12 | 0.797 |  |  |
| Total | 55 | 1689661.68 | 1 |  |  |
| *Drought intensity year 1: Control* |  |  |  |  |  |
| Drought year 2 | 1 | 38212.51 | 0.069 | 1.32 | 0.001 |
| Residual | 18 | 519204.18 | 0.931 |  |  |
| Total | 19 | 557416.69 | 1 |  |  |
| *Drought intensity year 1: Mild drought* |  |  |  |  |  |
| swd_2021 | 1 | 36122.2 | 0.088 | 1.35 | 0.025 |
| Residual | 14 | 375450.09 | 0.912 |  |  |
| Total | 15 | 411572.29 | 1 |  |  |
| *Drought intensity year 1: Severe drought* |  |  |  |  |  |
| swd_2021 | 1 | 41649.26 | 0.079 | 1.54 | 0.011 |
| Residual | 18 | 485291.5 | 0.921 |  |  |
| Total | 19 | 526940.76 | 1 |  |  |
| ***Recovery*** |  |  |  |  |  |
| Drought year 2 | 1 | 95709.51 | 0.053 | 3.15 | 0.001 |
| Plant community | 1 | 36583.63 | 0.02 | 1.2 | 0.078 |
| Drought intensity year 1 | 2 | 119222.06 | 0.066 | 1.96 | 0.001 |
| Drought year 2 x Plant community | 1 | 32402.01 | 0.018 | 1.06 | 0.229 |
| Drought year 2 x Drought intensity year 1 | 2 | 70579.29 | 0.039 | 1.16 | 0.079 |
| Plant community x Drought intensity year 1 | 2 | 59360.18 | 0.033 | 0.98 | 0.513 |
| Residual | 46 | 1399753.7 | 0.772 |  |  |
| Total | 55 | 1813610.38 | 1 |  |  |
| *Drought intensity year 1: Control* |  |  |  |  |  |
| Drought year 2 | 1 | 40226.594 | 0.072 | 1.392 | 0.009 |
| Residual | 18 | 520025.132 | 0.928 |  |  |
| Total | 19 | 560251.725 | 1.000 |  |  |
| *Drought intensity year 1: Mild drought* |  |  |  |  |  |
| Drought year 2 | 1 | 51633.138 | 0.105 | 1.639 | 0.002 |
| Residual | 14 | 440944.410 | 0.895 |  |  |
| Total | 15 | 492577.549 | 1.000 |  |  |
| *Drought intensity year 1: Severe drought* |  |  |  |  |  |
| Drought year 2 | 1 | 67666.037 | 0.118 | 2.419 | 0.001 |
| Residual | 18 | 503459.646 | 0.882 |  |  |
| Total | 19 | 571125.683 | 1.000 |  |  |
| **Fungal community composition** |  |  |  |  |  |
| ***Peak Drought*** | *df* | *SumOfSqs* | *R2* | *statistic* | *p.value* |
| Drought year 2 | 1 | 2417.81 | 0.027 | 1.53 | 0.001 |
| Plant community | 1 | 2174.47 | 0.024 | 1.37 | 0.001 |
| Drought intensity year 1 | 2 | 3541.85 | 0.04 | 1.12 | 0.013 |
| Drought year 2 x Plant community | 1 | 1740.55 | 0.02 | 1.1 | 0.091 |
| Drought year 2 x Drought intensity year 1 | 2 | 3159.94 | 0.035 | 1 | 0.492 |
| Plant community x Drought intensity year 1 | 2 | 3215.59 | 0.036 | 1.02 | 0.339 |
| Residual | 46 | 72792.65 | 0.818 |  |  |
| Total | 55 | 89042.87 | 1 |  |  |
| ***Recovery*** |  |  |  |  |  |
| Drought year 2 | 1 | 1774.39 | 0.02 | 1.13 | 0.025 |
| Plant community | 1 | 2101.16 | 0.024 | 1.34 | 0.001 |
| Drought intensity year 1 | 2 | 3456.98 | 0.039 | 1.1 | 0.021 |
| Drought year 2 x Plant community | 1 | 1671.62 | 0.019 | 1.07 | 0.138 |
| Drought year 2 x Drought intensity year 1 | 2 | 3301.18 | 0.038 | 1.06 | 0.136 |
| Plant community x Drought intensity year 1 | 2 | 3221.27 | 0.037 | 1.03 | 0.241 |
| Residual | 46 | 71997.89 | 0.823 |  |  |
| Total | 55 | 87524.48 | 1 |  |  |

Table S2. Variables associated with prokaryote and fungal community composition. Significance of constrained ordination (RDA) on prokaryote and fungal community β-diversity at peak drought and recovery. Significance was tested using anova.cca() to determine the global model significance (‘Model’), the significance of each term (arrow) and the significance of each axis. Soil legacies of increasing drought intensity (soil water deficit, SWD in 2020), microbial biomass, dissolved organic nitrogen (DON), community aboveground productivity (aboveground biomass, g m^-2^), and soil N-NO_3_^-^ (nitrate).

| **Prokaryote communities** | | | | |
| --- | --- | --- | --- | --- |
| ***Peak drought*** |  |  |  |  |
| term | df | Variance | statistic | p.value |
| Model | 2 | 2255.03 | 2.125 | 0.001 |
| Residual | 53 | 28127.24 |  |  |
| SWD 2020 | 1 | 1293.90 | 2.438 | 0.001 |
| Microbial biomass | 1 | 961.13 | 1.811 | 0.001 |
| Residual | 53 | 28127.24 |  |  |
| RDA1 | 1 | 1302.26 | 2.454 | 0.001 |
| RDA2 | 1 | 952.77 | 1.795 | 0.001 |
| Residual | 53 | 28127.24 |  |  |
| Model R2 adjusted |  |  |  | 0.039 |
| ***Recovery*** |  |  |  |  |
| Model | 2 | 2547.95 | 2.233 | 0.001 |
| Residual | 53 | 30232.60 |  |  |
| SWD 2020 | 1 | 1609.74 | 2.822 | 0.001 |
| DON | 1 | 938.21 | 1.645 | 0.007 |
| Residual | 53 | 30232.60 |  |  |
| RDA1 | 1 | 1851.38 | 3.246 | 0.001 |
| RDA2 | 1 | 696.57 | 1.221 | 0.052 |
| Residual | 53 | 30232.60 |  |  |
| Model R2 adjusted |  |  |  | 0.043 |
| **Fungal communities** |  |  |  |  |
| ***Peak drought*** |  |  |  |  |
| Model | 2 | 78.96 | 1.359 | 0.001 |
| Residual | 53 | 1540.00 |  |  |
| SWD 2020 | 1 | 35.28 | 1.214 | 0.001 |
| Microbial biomass | 1 | 43.68 | 1.503 | 0.001 |
| Residual | 53 | 1540.00 |  |  |
| RDA1 | 1 | 43.80 | 1.507 | 0.001 |
| RDA2 | 1 | 35.16 | 1.210 | 0.004 |
| Residual | 53 | 1540.00 |  |  |
| Model R2 adjusted |  |  |  | 0.013 |
| ***Recovery*** |  |  |  |  |
| Model | 3 | 102.79 | 1.198 | 0.001 |
| Residual | 52 | 1487.31 |  |  |
| SWD 2020 | 1 | 35.83 | 1.253 | 0.001 |
| Nitrate | 1 | 33.72 | 1.179 | 0.010 |
| Aboveground biomass | 1 | 33.24 | 1.162 | 0.011 |
| Residual | 52 | 1487.31 |  |  |
| RDA1 | 1 | 37.86 | 1.324 | 0.002 |
| RDA2 | 1 | 35.09 | 1.227 | 0.010 |
| RDA3 | 1 | 29.84 | 1.043 | 0.284 |
| Residual | 52 | 1487.31 |  |  |
| Model R2 adjusted |  |  |  | 0.011 |

Table S3. Prokaryote and fungal community α-diversity (Shannon Index). Prokaryote and fungal communities after three weeks of drought or control (peak drought) and seven weeks after re-wetting (recovery) in year 2. Significance of plant community (fast- or slow-strategy plant community), soil legacies of increasing drought intensity (control, mild drought, or severe drought in year 1), drought (or control) in year 2, and all interactions were tested in linear models. Models were simplified to achieve the best fit parsimonious model, and significance was determined with ANOVA.

| **Peak drought - fungal communities** | df | sumsq | meansq | statistic | p.value |
| --- | --- | --- | --- | --- | --- |
| Plant community | 1 | 0.344 | 0.344 | 6.449 | 0.014 |
| Soil legacies of increasing drought intensity | 2 | 0.188 | 0.094 | 1.757 | 0.183 |
| Drought or control in year 2 | 1 | 0.089 | 0.089 | 1.663 | 0.203 |
| Residuals | 51 | 2.723 | 0.053 |  |  |
| **Recovery - fungal communities** | df | sumsq | meansq | statistic | p.value |
| Plant community | 1 | 0.014 | 0.014 | 0.488 | 0.488 |
| Soil legacies of increasing drought intensity | 2 | 0.02 | 0.01 | 0.346 | 0.709 |
| Drought or control in year 2 | 1 | 0.024 | 0.024 | 0.813 | 0.372 |
| Residuals | 51 | 1.498 | 0.029 |  |  |
| **Peak drought - prokaryote communities** | df | sumsq | meansq | statistic | p.value |
| Plant community | 1 | 0.09 | 0.09 | 0.728 | 0.397 |
| Soil legacies of increasing drought intensity | 2 | 0.286 | 0.143 | 1.159 | 0.322 |
| Drought or control in year 2 | 1 | 0.086 | 0.086 | 0.695 | 0.408 |
| Residuals | 51 | 6.281 | 0.123 |  |  |
| **Recovery - prokaryote communities** | df | sumsq | meansq | statistic | p.value |
| Plant community | 1 | 0.025 | 0.025 | 0.364 | 0.549 |
| Soil legacies of increasing drought intensity | 2 | 0.121 | 0.061 | 0.893 | 0.416 |
| Drought or control in year 2 | 1 | 0.792 | 0.792 | 11.663 | 0.001 |
| Residuals | 51 | 3.466 | 0.068 |  |  |

Table S4. The relative abundance of prokaryote and fungal phyla and families. Soil legacies of increasing drought intensity (control, mild drought, or severe drought in year 1), drought (or control) in year 2, plant community (fast- or slow-strategy), and their interactions on the relative abundance of prokaryote and fungal phyla, and the 20 most abundant prokaryote and fungal families (excluding families that were ‘unknown’). We tested the effects of soil legacies, drought, plant community and their interactions at peak drought year 2 (after four weeks of drought) and at recovery (seven weeks after re-wetting). We used linear models and anova, with a Benjamini-Hochberg adjustment (Benjamini & Hochberg, 1995) using the R function p.adjust to correct P values for multiple comparisons.

{see excel sheet}

Table S5. Soil functioning in year 2 (multivariate relationships). (A) PERMANOVA (adonis2 output) showing the effect of soil legacies of increasing drought intensity (control, mild drought, or severe drought in 2020), the drought (or control) in year 2 and plant community (fast- or slow-strategy) on soil functioning in year 2 at three sampling points: the spring before the drought, at peak drought, and at recovery. The interactions between soil legacies of increasing drought intensity and plant community were further explored by dividing the dataset into the year 1 control, mild drought, and severe drought treatments and testing the effect of plant strategy on soil functioning. (B) Significance of constrained ordination (RDA) on soil functioning in year 2 (Fig. 6) in the spring before the drought, at peak drought, and at recovery. Significance was tested using anova.cca() to determine the global model significance (‘Model’), the significance of each term (arrow) and the significance of each axis. Soil legacies of increasing drought intensity (SWD 2020), plant community aboveground biomass.

| PERMANOVA |  |  |  |  |  |
| --- | --- | --- | --- | --- | --- |
| ***(A) Baseline*** | ***df*** | ***SumOfSqs*** | ***R2*** | ***statistic*** | ***p.value*** |
| Plant community | 1 | 12.796 | 0.068 | 2.321 | 0.035 |
| Soil legacies of increasing drought intensity | 2 | 36.817 | 0.195 | 3.338 | 0.001 |
| Plant community x Soil legacies | 2 | 18.075 | 0.096 | 1.639 | 0.069 |
| Residual | 22 | 121.312 | 0.642 |  |  |
| Total | 27 | 189.000 | 1.000 |  |  |
|  |  |  |  |  |  |
| ***Pairwise Adonis - Soil legacies*** | ***df*** | ***SumsOfSqs*** | ***F.Model*** | ***R2*** | ***p.adjusted*** |
| control vs mild drought | 1 | 16.811 | 2.530 | 0.137 | 0.072 |
| control vs severe drought | 1 | 23.290 | 4.601 | 0.204 | 0.006 |
| mild drought vs severe drought | 1 | 14.582 | 2.182 | 0.120 | 0.171 |
|  |  |  |  |  |  |
| ***(B) Peak drought*** | ***df*** | ***SumOfSqs*** | ***R2*** | ***statistic*** | ***p.value*** |
| Drought year 2 | 1 | 127.993 | 0.332 | 30.255 | 0.001 |
| Plant community | 1 | 8.246 | 0.021 | 1.949 | 0.086 |
| Soil legacies of increasing drought intensity | 2 | 16.894 | 0.044 | 1.997 | 0.047 |
| Drought year 2 x Plant community | 1 | 6.182 | 0.016 | 1.461 | 0.173 |
| Drought year 2 x Soil legacies | 2 | 13.729 | 0.036 | 1.623 | 0.113 |
| Plant community x Soil legacies | 2 | 17.355 | 0.045 | 2.051 | 0.036 |
| Residual | 46 | 194.600 | 0.505 |  |  |
| Total | 55 | 385.000 | 1.000 |  |  |
| ***Peak drought interaction effects (Plant community x Soil legacies of increasing drought intensity)*** | | | | | |
| ***Control year 1*** | ***df*** | ***SumOfSqs*** | ***R2*** | ***statistic*** | ***p.value*** |
| Plant community | 1 | 4.682 | 0.035 | 0.657 | 0.568 |
| Residual | 18 | 128.318 | 0.965 |  |  |
| Total | 19 | 133.000 | 1.000 |  |  |
| ***Mild drought year 1*** |  |  |  |  |  |
| Plant community | 1 | 3.544 | 0.034 | 0.489 | 0.823 |
| Residual | 14 | 101.456 | 0.966 |  |  |
| Total | 15 | 105.000 | 1.000 |  |  |
| ***Severe drought year 1*** |  |  |  |  |  |
| Plant community | 1 | 14.026 | 0.105 | 2.122 | 0.087 |
| Residual | 18 | 118.974 | 0.895 |  |  |
| Total | 19 | 133.000 | 1.000 |  |  |
|  |  |  |  |  |  |
| ***Pairwise Adonis - Soil legacies*** | ***df*** | ***SumsOfSqs*** | ***F.Model*** | ***R2*** | ***p.adjusted*** |
| control vs mild drought | 1 | 7.659 | 1.032 | 0.029 | 1.000 |
| control vs severe drought | 1 | 12.741 | 1.975 | 0.049 | 0.273 |
| mild drought vs severe drought | 1 | 4.464 | 0.636 | 0.018 | 1.000 |
|  |  |  |  |  |  |
| ***(C) Recovery*** | ***df*** | ***SumOfSqs*** | ***R2*** | ***statistic*** | ***p.value*** |
| Drought year 2 | 1 | 22.537 | 0.059 | 4.264 | 0.002 |
| Plant community | 1 | 39.835 | 0.103 | 7.538 | 0.001 |
| Soil legacies of increasing drought intensity | 2 | 40.526 | 0.105 | 3.834 | 0.001 |
| Drought year 2 x Plant community | 1 | 5.847 | 0.015 | 1.106 | 0.354 |
| Drought year 2 x Soil legacies | 2 | 18.561 | 0.048 | 1.756 | 0.058 |
| Plant community x Soil legacies | 2 | 14.588 | 0.038 | 1.380 | 0.171 |
| Residual | 46 | 243.106 | 0.631 |  |  |
| Total | 55 | 385.000 | 1.000 |  |  |
|  |  |  |  |  |  |
| ***Pairwise Adonis - Soil legacies*** | ***df*** | ***SumsOfSqs*** | ***F.Model*** | ***R2*** | ***p.adjusted*** |
| control vs mild drought | 1 | 12.318 | 1.746 | 0.049 | 0.303 |
| control vs severe drought | 1 | 31.008 | 5.054 | 0.117 | 0.003 |
| mild drought vs severe drought | 1 | 16.268 | 2.562 | 0.070 | 0.057 |
|  |  |  |  |  |  |
| RDA |  |  |  |  |  |
| ***(D) Baseline*** | ***df*** | ***Variance*** | ***statistic*** | ***p.value*** |  |
| Model | 2 | 1.345 | 2.972 | 0.002 |  |
| Residual | 25 | 5.655 |  |  |  |
| SWD 2020 | 1 | 0.818 | 3.618 | 0.002 |  |
| Aboveground biomass | 1 | 0.526 | 2.327 | 0.033 |  |
| Residual | 25 | 5.655 |  |  |  |
| RDA1 | 1 | 0.821 | 3.628 | 0.004 |  |
| RDA2 | 1 | 0.524 | 2.316 | 0.023 |  |
| Residual | 25 | 5.655 |  |  |  |
| R2 adjusted |  |  |  | 0.127 |  |
| ***(E) Peak drought*** | ***df*** | ***Variance*** | ***statistic*** | ***p.value*** |  |
| Model | 2 | 2.558 | 15.258 | 0.001 |  |
| Residual | 53 | 4.442 |  |  |  |
| SWD 2020 | 1 | 0.269 | 3.208 | 0.010 |  |
| Microbial biomass | 1 | 2.289 | 27.309 | 0.001 |  |
| Residual | 53 | 4.442 |  |  |  |
| RDA1 | 1 | 2.301 | 27.448 | 0.001 |  |
| RDA2 | 1 | 0.257 | 3.068 | 0.008 |  |
| Residual | 53 | 4.442 |  |  |  |
| R2 adjusted |  |  |  | 0.341 |  |
| ***(F) Recovery*** | ***df*** | ***Variance*** | ***statistic*** | ***p.value*** |  |
| Model | 4 | 1.786 | 4.368 | 0.001 |  |
| Residual | 51 | 5.214 |  |  |  |
| SWD 2020 | 1 | 0.587 | 5.738 | 0.001 |  |
| DOC | 1 | 0.609 | 5.961 | 0.001 |  |
| Microbial biomass | 1 | 0.364 | 3.558 | 0.004 |  |
| Belowground biomass | 1 | 0.226 | 2.215 | 0.038 |  |
| Residual | 51 | 5.214 |  |  |  |
| RDA1 | 1 | 0.959 | 9.376 | 0.001 |  |
| RDA2 | 1 | 0.560 | 5.481 | 0.002 |  |
| Residual | 51 | 5.214 |  |  |  |
| R2 adjusted |  |  |  | 0.197 |  |

Table S6. Soil functioning in year 2 (univariate relationships). The effects of soil legacies of increasing drought intensity (control, mild drought, or severe drought in 2020), the year 2 drought (or control), plant community (fast- or slow-strategy) and all 2-way interactions on individual soil functions: Soil ammonium (N-NH_4_^+^), nitrate (N-NO_3_^-^), microbial biomass carbon, and the potential enzyme activity of β-glucosidase (pGLC), β-N-acetylglucosaminidase (pNAG), urease (pURE), peroxidase (pPER), and phenoloxidase (pPOX) at three timepoints in year 2: before the drought, at peak drought, and at recovery. Significance was tested with linear models (N-NH_4_^+^, N-NO_3_^-^, microbial biomass) and linear mixed effects models (all potential enzyme activities, the experimental unit was used as the random factor to account for laboratory replicates). Significant differences between levels of a treatment are based on a Tukey post-hoc test.

| ***pGLC*** |  |  |  |  |  |
| --- | --- | --- | --- | --- | --- |
| **Baseline** | numDF | denDF | F-value | p-value |  |
| (Intercept) | 1 | 84 | 2681.452 | 0.000 |  |
| Plant community | 1 | 22 | 0.589 | 0.451 |  |
| Drought intensity year 1 | 2 | 22 | 9.973 | 0.001 |  |
| Plant community x Drought intensity year 1 | 2 | 22 | 0.52 | 0.602 |  |
| R2 adjusted |  |  |  | 0.233 |  |
| **Peak drought** | numDF | denDF | F-value | p-value |  |
| (Intercept) | 1 | 168 | 5286.014 | 0.000 |  |
| Plant community | 1 | 49 | 0.822 | 0.369 |  |
| Drought intensity year 1 | 2 | 49 | 0.151 | 0.861 |  |
| Drought year 2 | 1 | 49 | 3.006 | 0.089 |  |
| Drought year 2 x Drought intensity year 1 | 2 | 49 | 2.494 | 0.093 |  |
| R2 adjusted |  |  |  | 0.100 |  |
| **Recovery** | numDF | denDF | F-value | p-value |  |
| (Intercept) | 1 | 168 | 11983.843 | 0.000 |  |
| Plant community | 1 | 49 | 0.142 | 0.708 |  |
| Drought intensity year 1 | 2 | 49 | 1.154 | 0.324 |  |
| Drought year 2 | 1 | 49 | 0.548 | 0.463 |  |
| Plant community x Drought intensity year 1 | 2 | 49 | 2.979 | 0.060 |  |
| R2 adjusted |  |  |  | 0.087 |  |
| ***pNAG*** |  |  |  |  |  |
| **Baseline** | numDF | denDF | F-value | p-value |  |
| (Intercept) | 1 | 84 | 2459.573 | 0.000 |  |
| Plant community | 1 | 22 | 7.324 | 0.013 |  |
| Drought intensity year 1 | 2 | 22 | 6.931 | 0.005 |  |
| Plant community x Drought intensity year 1 | 2 | 22 | 0.461 | 0.637 |  |
| R2 adjusted |  |  |  | 0.261 |  |
| **Peak drought** | numDF | denDF | F-value | p-value |  |
| (Intercept) | 1 | 168 | 5095.889 | 0.000 |  |
| Plant community | 1 | 50 | 2.681 | 0.108 |  |
| Drought intensity year 1 | 2 | 50 | 14.919 | 0.000 |  |
| Drought year 2 | 1 | 50 | 104.106 | 0.000 |  |
| Plant community x Drought year 2 | 1 | 50 | 5.831 | 0.019 |  |
| R2 adjusted |  |  |  | 0.555 |  |
| **Recovery** | numDF | denDF | F-value | p-value |  |
| (Intercept) | 1 | 168 | 5777.522 | 0.000 |  |
| Plant community | 1 | 51 | 0.28 | 0.599 |  |
| Drought intensity year 1 | 2 | 51 | 16.151 | 0.000 |  |
| Drought year 2 | 1 | 51 | 30.044 | 0.000 |  |
| R2 adjusted |  |  |  | 0.366 |  |
| ***pPER*** |  |  |  |  |  |
| **Baseline** | numDF | denDF | F-value | p-value |  |
| (Intercept) | 1 | 56 | 3417.022 | 0.000 |  |
| Plant community | 1 | 22 | 0.875 | 0.360 |  |
| Drought intensity year 1 | 2 | 22 | 5.406 | 0.012 |  |
| Plant community x Drought intensity year 1 | 2 | 22 | 1.752 | 0.197 |  |
| R2 adjusted |  |  |  | 0.278 |  |
| **Peak drought** | numDF | denDF | F-value | p-value |  |
| (Intercept) | 1 | 109 | 4714.707 | 0.000 |  |
| Plant community | 1 | 51 | 3.311 | 0.075 |  |
| Drought intensity year 1 | 2 | 51 | 0.357 | 0.702 |  |
| Drought year 2 | 1 | 51 | 31.547 | 0.000 |  |
| R2 adjusted |  |  |  | 0.278 |  |
| **Recovery** | numDF | denDF | F-value | p-value |  |
| (Intercept) | 1 | 112 | 2315.279 | 0.000 |  |
| Plant community | 1 | 49 | 4.515 | 0.039 |  |
| Drought intensity year 1 | 2 | 49 | 3.853 | 0.028 |  |
| Drought year 2 | 1 | 49 | 4.425 | 0.041 |  |
| Drought year 2 x Drought intensity year 1 | 2 | 49 | 4.91 | 0.011 |  |
| R2 adjusted |  |  |  | 0.241 |  |
| ***pPOX*** |  |  |  |  |  |
| **Baseline** | numDF | denDF | F-value | p-value |  |
| (Intercept) | 1 | 56 | 2537.682 | 0.000 |  |
| Plant community | 1 | 22 | 2.516 | 0.127 |  |
| Drought intensity year 1 | 2 | 22 | 0.95 | 0.402 |  |
| Plant community x Drought intensity year 1 | 2 | 22 | 9.149 | 0.001 |  |
| R2 adjusted |  |  |  | 0.321 |  |
| **Peak drought** | numDF | denDF | F-value | p-value |  |
| (Intercept) | 1 | 109 | 5027.549 | 0.000 |  |
| Plant community | 1 | 49 | 3.604 | 0.064 |  |
| Drought intensity year 1 | 2 | 49 | 1.66 | 0.201 |  |
| Drought year 2 | 1 | 49 | 10.375 | 0.002 |  |
| Plant community x Drought intensity year 1 | 2 | 49 | 5.096 | 0.010 |  |
| R2 adjusted |  |  |  | 0.222 |  |
| **Recovery** | numDF | denDF | F-value | p-value |  |
| (Intercept) | 1 | 112 | 2113.435 | 0.000 |  |
| Plant community | 1 | 51 | 6.04 | 0.017 |  |
| Drought intensity year 1 | 2 | 51 | 9.124 | 0.000 |  |
| Drought year 2 | 1 | 51 | 1.427 | 0.238 |  |
| R2 adjusted |  |  |  | 0.195 |  |
| ***pURE*** |  |  |  |  |  |
| **Baseline** | numDF | denDF | F-value | p-value |  |
| (Intercept) | 1 | 84 | 5421.614 | 0.000 |  |
| Plant community | 1 | 22 | 5.21 | 0.032 |  |
| Drought intensity year 1 | 2 | 22 | 1.978 | 0.162 |  |
| Plant community x Drought intensity year 1 | 2 | 22 | 0.192 | 0.827 |  |
| R2 adjusted |  |  |  | 0.102 |  |
| **Peak drought** | numDF | denDF | F-value | p-value |  |
| (Intercept) | 1 | 168 | 2726.922 | 0.000 |  |
| Plant community | 1 | 49 | 2.127 | 0.151 |  |
| Drought intensity year 1 | 2 | 49 | 2.165 | 0.126 |  |
| Drought year 2 | 1 | 49 | 3.409 | 0.071 |  |
| Drought year 2 x Drought intensity year 1 | 2 | 49 | 2.668 | 0.079 |  |
| R2 adjusted |  |  |  | 0.170 |  |
| **Recovery** | numDF | denDF | F-value | p-value |  |
| (Intercept) | 1 | 168 | 2557.763 | 0.000 |  |
| Plant community | 1 | 51 | 1.841 | 0.181 |  |
| Drought intensity year 1 | 2 | 51 | 0.842 | 0.437 |  |
| Drought year 2 | 1 | 51 | 0.92 | 0.342 |  |
| R2 adjusted |  |  |  | 0.063 |  |
|  |  |  |  |  |  |
| ***N-NO3-*** |  |  |  |  |  |
| **Baseline** | Df | Sum Sq | Mean Sq | F value | Pr(>F) |
| Plant community | 1 | 2.582 | 2.582 | 0.694 | 0.414 |
| Drought intensity year 1 | 2 | 2.151 | 1.075 | 0.289 | 0.752 |
| Plant community x Drought intensity year 1 | 2 | 4.996 | 2.498 | 0.672 | 0.521 |
| Residuals | 22 | 81.807 | 3.718 |  |  |
| R2 adjusted |  |  |  |  | -0.097 |
| **Peak drought** | Df | Sum Sq | Mean Sq | F value | Pr(>F) |
| Plant community | 1 | 0.905 | 0.905 | 1.229 | 0.273 |
| Drought intensity year 1 | 2 | 0.512 | 0.256 | 0.348 | 0.708 |
| Drought year 2 | 1 | 82.939 | 82.939 | 112.596 | 0 |
| Residuals | 51 | 37.567 | 0.737 |  |  |
| R2 adjusted |  |  |  |  | 0.668 |
| **Recovery** | Df | Sum Sq | Mean Sq | F value | Pr(>F) |
| Plant community | 1 | 52.366 | 52.366 | 17.013 | 0 |
| Drought intensity year 1 | 2 | 4.615 | 2.307 | 0.750 | 0.478 |
| Drought year 2 | 1 | 8.98 | 8.98 | 2.917 | 0.094 |
| Residuals | 51 | 156.977 | 3.078 |  |  |
| R2 adjusted |  |  |  |  | 0.241 |
| ***N-NH4+*** |  |  |  |  |  |
| **Baseline** | Df | Sum Sq | Mean Sq | F value | Pr(>F) |
| Plant community | 1 | 481.662 | 481.662 | 0.089 | 0.768 |
| Drought intensity year 1 | 2 | 22484.816 | 11242.408 | 2.085 | 0.148 |
| Plant community x Drought intensity year 1 | 2 | 1334.044 | 667.022 | 0.124 | 0.884 |
| Residuals | 22 | 118644.45 | 5392.929 |  |  |
| R2 adjusted |  |  |  |  | -0.019 |
| **Peak drought** | Df | Sum Sq | Mean Sq | F value | Pr(>F) |
| Plant community | 1 | 0.003 | 0.003 | 0.034 | 0.854 |
| Drought intensity year 1 | 2 | 0.202 | 0.101 | 1.206 | 0.308 |
| Drought year 2 | 1 | 4.531 | 4.531 | 54.182 | 0 |
| Plant community x Drought year 2 | 1 | 0.262 | 0.262 | 3.136 | 0.083 |
| Residuals | 50 | 4.182 | 0.084 |  |  |
| R2 adjusted |  |  |  |  | 0.499 |
| **Recovery** | Df | Sum Sq | Mean Sq | F value | Pr(>F) |
| Plant community | 1 | 41.891 | 41.891 | 24.025 | 0 |
| Drought intensity year 1 | 2 | 2.9 | 1.45 | 0.832 | 0.441 |
| Drought year 2 | 1 | 0.24 | 0.24 | 0.138 | 0.712 |
| Residuals | 51 | 88.926 | 1.744 |  |  |
| R2 adjusted |  |  |  |  | 0.284 |
| ***Microbial biomass carbon*** |  |  |  |  |  |
| **Baseline** | Df | Sum Sq | Mean Sq | F value | Pr(>F) |
| Plant community | 1 | 481.662 | 481.662 | 0.089 | 0.768 |
| Drought intensity year 1 | 2 | 22484.816 | 11242.408 | 2.085 | 0.148 |
| Plant community x Drought intensity year 1 | 2 | 1334.044 | 667.022 | 0.124 | 0.884 |
| Residuals | 22 | 118644.45 | 5392.929 |  |  |
| R2 adjusted |  |  |  |  | -0.019 |
| **Peak drought** | Df | Sum Sq | Mean Sq | F value | Pr(>F) |
| Plant community | 1 | 8425.147 | 8425.147 | 1.965 | 0.167 |
| Drought intensity year 1 | 2 | 4824.898 | 2412.449 | 0.563 | 0.573 |
| Drought year 2 | 1 | 6302750.2 | 6302750.228 | 1469.693 | 0 |
| Residuals | 51 | 218712.58 | 4288.482 |  |  |
| R2 adjusted |  |  |  |  | 0.964 |
| **Recovery** | Df | Sum Sq | Mean Sq | F value | Pr(>F) |
| Plant community | 1 | 21756.028 | 21756.028 | 3.602 | 0.064 |
| Drought intensity year 1 | 2 | 6534.94 | 3267.47 | 0.541 | 0.586 |
| Drought year 2 | 1 | 15160.456 | 15160.456 | 2.510 | 0.12 |
| Plant community x Drought intensity year 1 | 2 | 49953.382 | 24976.691 | 4.135 | 0.022 |
| Residuals | 49 | 295994.79 | 6040.71 |  |  |
| R2 adjusted |  |  |  |  | 0.147 |

Table S7. The effect of soil legacies of increasing drought intensity (soil water deficit, SWD, in 2020) on the resistance and recovery of plant community aboveground biomass to drought in year 2 (2021). Baseline normalized resistance and recovery were calculated using the mean aboveground biomass of communities that were maintained at control conditions in both years as the continuous baseline (Ingrisch & Bahn, 2018). Resistance was determined after 3-weeks of drought (peak drought) and recovery was determined seven weeks after re-wetting. Generalized additive models (GAMs) were used to determine significant relations between the soil water deficit in year 1 (SWD 2020) and resistance or recovery of fast- and slow-strategy plant communities. The GAM model tested the effect of plant community (factor) and SWD 2020 (continuous) and their interaction on resistance or recovery in year 2. The significant interaction between SWD 2020 and plant community necessitated a second GAM, which tested the effect of SWD 2020 on resistance or recovery within each plant community. The Gamma(log = link) family was chosen to fit the GAMs as this resulted in the best fit.

| **Peak drought year 2 (resistance of ANPP)** | | | | | | | | | |
| --- | --- | --- | --- | --- | --- | --- | --- | --- | --- |
| *Parameter* | *Coefficient* | *SE* | *CI_low* | *CI_high* | *t / F* | *df* | *df_error* | *p* | *Component* |
| (Intercept) | -0.237 | 0.015 | -0.266 | -0.207 | -16.115 |  | 51.298 | 0.000 | conditional |
| Plant community | -0.087 | 0.021 | -0.128 | -0.045 | -4.179 |  | 51.298 | 0.000 | conditional |
| Drought intensity year 1 (SWD 2020) |  |  |  |  | 7.797 | 1.000 |  | 0.007 | smooth terms |
| Drought intensity year 1 (SWD 2020) x Plant community |  |  |  |  | 3.879 | 1.702 |  | 0.025 | smooth terms |
| ***Interaction model*** |  |  |  |  |  |  |  |  |  |
| *Parameter* | *Coefficient* | *SE* | *CI_low* | *CI_high* | *t / F* | *df* | *df_error* | *p* | *Component* |
| (Intercept) | 0.743 | 0.016 | 0.711 | 0.774 | 47.490 |  | 49.149 | 0.000 | conditional |
| Plant community | 0.098 | 0.022 | 0.053 | 0.142 | 4.426 |  | 49.149 | 0.000 | conditional |
| Fast-strategy plant community |  |  |  |  | 1.017 | 1.714 |  | 0.351 | smooth terms |
| Slow-strategy plant community |  |  |  |  | 5.234 | 3.137 |  | 0.002 | smooth terms |
| **Recovery year 2 (recovery of ANPP)** | | | | | | | | | |
| *Parameter* | *Coefficient* | *SE* | *CI_low* | *CI_high* | *t / F* | *df* | *df_error* | *p* | *Component* |
| (Intercept) | 0.197 | 0.020 | 0.154 | 0.239 | 9.610 |  | 22.717 | 0.000 | conditional |
| Plant community | 0.001 | 0.029 | -0.059 | 0.061 | 0.044 |  | 22.717 | 0.965 | conditional |
| Drought intensity year 1 (SWD 2020) |  |  |  |  | 3.468 | 2.283 |  | 0.034 | smooth terms |
| Drought intensity year 1 (SWD 2020) x Plant community |  |  |  |  | 8.287 | 1.000 |  | 0.008 | smooth terms |
| ***Interaction model*** |  |  |  |  |  |  |  |  |  |
| *Parameter* | *Coefficient* | *SE* | *CI_low* | *CI_high* | *t / F* | *df* | *df_error* | *p* | *Component* |
| (Intercept) | 1.226 | 0.036 | 1.152 | 1.299 | 34.409 |  | 21.976 | 0.000 | conditional |
| Plant community | -0.006 | 0.050 | -0.111 | 0.098 | -0.126 |  | 21.976 | 0.901 | conditional |
| Fast-strategy plant community |  |  |  |  | 4.745 | 2.009 |  | 0.015 | smooth terms |
| Slow-strategy plant community |  |  |  |  | 1.934 | 2.016 |  | 0.166 | smooth terms |

**Table S8. Soil community structure and function relationships with plant resistance and recovery.** Relationships between measured soil variables and year 2 drought resistance and recovery of aboveground net primary productivity (ANPP) to the subsequent drought. Only resistance of plant communities that were destructively harvested at peak drought are included (i.e., in the experimental units where soil parameters were measured, n = 28). Relationships were determined with GAM models to first establish if there was an interaction between plant community and the measured variable, and then the relationships within each plant community were tested. The Gamma(log = link) family was chosen to fit the GAMs as this resulted in the best fit.

| **Reistance of ANPP** |  |  |  |  |  |  |  |  |  |
| --- | --- | --- | --- | --- | --- | --- | --- | --- | --- |
| Parameter | Coefficient | SE | CI_low | CI_high | t / F | df | df_error | p | Component |
| (Intercept) | -0.239 | 0.032 | -0.305 | -0.173 | -7.51 |  | 24 | 0.000 | conditional |
| Plant community | -0.05 | 0.045 | -0.143 | 0.043 | -1.12 |  | 24 | 0.274 | conditional |
| Diversisporaceae |  |  |  |  | 4.745 | 1 |  | 0.039 | smooth terms |
| Diversisporaceae x plant community |  |  |  |  | 1.285 | 1 |  | 0.268 | smooth terms |
| Fast-strategy plant community |  |  |  |  | 0.056 | 1 |  | 0.814 | smooth terms |
| Slow-strategy plant community |  |  |  |  | 4.882 | 1 |  | 0.037 | smooth terms |
| (Intercept) | -0.235 | 0.02 | -0.277 | -0.194 | -11.716 |  | 24 | 0.000 | conditional |
| Plant community | -0.076 | 0.028 | -0.135 | -0.018 | -2.685 |  | 24 | 0.013 | conditional |
| Mortierellaceae |  |  |  |  | 21.652 | 1 |  | 0.000 | smooth terms |
| Mortierellaceae x plant community |  |  |  |  | 17.864 | 1 |  | 0.000 | smooth terms |
| Fast-strategy plant community |  |  |  |  | 1.378 | 1 |  | 0.252 | smooth terms |
| Slow-strategy plant community |  |  |  |  | 22.237 | 1 |  | 0.000 | smooth terms |
| (Intercept) | -0.245 | 0.028 | -0.303 | -0.187 | -8.742 |  | 24 | 0.000 | conditional |
| Plant community | -0.061 | 0.04 | -0.142 | 0.021 | -1.533 |  | 24 | 0.138 | conditional |
| NAG |  |  |  |  | 6.109 | 1 |  | 0.021 | smooth terms |
| NAG x plant community |  |  |  |  | 3.795 | 1 |  | 0.063 | smooth terms |
| Fast-strategy plant community |  |  |  |  | 0.066 | 1 |  | 0.800 | smooth terms |
| Slow-strategy plant community |  |  |  |  | 6.849 | 1 |  | 0.015 | smooth terms |
| (Intercept) | -0.213 | 0.023 | -0.262 | -0.165 | -9.088 |  | 22.952 | 0.000 | conditional |
| Plant community | -0.099 | 0.033 | -0.168 | -0.03 | -2.984 |  | 22.952 | 0.007 | conditional |
| Microbial biomass |  |  |  |  | 4.595 | 2.048 |  | 0.014 | smooth terms |
| Microbial biomass x plant community |  |  |  |  | 4.131 | 1 |  | 0.054 | smooth terms |
| Fast-strategy plant community |  |  |  |  | 0.146 | 1.35 |  | 0.798 | smooth terms |
| Slow-strategy plant community |  |  |  |  | 8.184 | 1 |  | 0.009 | smooth terms |
| **Recovery of ANPP** |  |  |  |  |  |  |  |  |  |
| (Intercept) | 0.2 | 0.023 | 0.153 | 0.247 | 8.783 |  | 23.39 | 0.000 | conditional |
| Plant community | 0.001 | 0.032 | -0.066 | 0.067 | 0.026 |  | 23.39 | 0.980 | conditional |
| PC1 |  |  |  |  | 1.795 | 1 |  | 0.193 | smooth terms |
| PC1 x plant community |  |  |  |  | 4.978 | 1.61 |  | 0.013 | smooth terms |
| Fast-strategy plant community |  |  |  |  | 4.331 | 1.61 |  | 0.020 | smooth terms |
| Slow-strategy plant community |  |  |  |  | 1.125 | 1.199 |  | 0.257 | smooth terms |
| (Intercept) | 0.198 | 0.024 | 0.148 | 0.248 | 8.212 |  | 24 | 0.000 | conditional |
| Plant community | 0 | 0.034 | -0.07 | 0.071 | 0.013 |  | 24 | 0.990 | conditional |
| pNAG activity |  |  |  |  | 0.21 | 1 |  | 0.651 | smooth terms |
| pNAG activity x plant community |  |  |  |  | 4.071 | 1 |  | 0.055 | smooth terms |
| Fast-strategy plant community |  |  |  |  | 6.428 | 1 |  | 0.018 | smooth terms |
| Slow-strategy plant community |  |  |  |  | 0.213 | 1 |  | 0.648 | smooth terms |
| (Intercept) | 0.181 | 0.027 | 0.126 | 0.236 | 6.809 |  | 24 | 0.000 | conditional |
| Plant community | -0.018 | 0.038 | -0.096 | 0.06 | -0.48 |  | 24 | 0.635 | conditional |
| Nitrosococcaceae |  |  |  |  | 0.181 | 1 |  | 0.674 | smooth terms |
| Nitrosococcaceae x plant community |  |  |  |  | 3.85 | 1 |  | 0.061 | smooth terms |
| Fast-strategy plant community |  |  |  |  | 4.297 | 1 |  | 0.049 | smooth terms |
| Slow-strategy plant community |  |  |  |  | 0.18 | 1 |  | 0.675 | smooth terms |
| (Intercept) | 0.201 | 0.022 | 0.157 | 0.246 | 9.35 |  | 22.638 | 0.000 | conditional |
| Plant community | 0.028 | 0.031 | -0.036 | 0.092 | 0.909 |  | 22.638 | 0.373 | conditional |
| Nitrospiraceae |  |  |  |  | 2.441 | 2.362 |  | 0.067 | smooth terms |
| Nitrospiraceae x plant community |  |  |  |  | 1.232 | 1 |  | 0.279 | smooth terms |
| Fast-strategy plant community |  |  |  |  | 4.576 | 1.804 |  | 0.019 | smooth terms |
| Slow-strategy plant community |  |  |  |  | 2.328 | 2.596 |  | 0.113 | smooth terms |

**References**

Cordero, I., Snell, H., & Bardgett, R. D. (2019). High throughput method for measuring urease activity in soil. *Soil Biology and Biochemistry*, *134*, 72–77. https://doi.org/10.1016/j.soilbio.2019.03.014

Ingrisch, J., & Bahn, M. (2018). Towards a Comparable Quantification of Resilience. *Trends in Ecology & Evolution*, *33*(4), 251–259. https://doi.org/10.1016/j.tree.2018.01.013

Jackson, C. R., Tyler, H. L., & Millar, J. J. (2013). Determination of Microbial Extracellular Enzyme Activity in Waters, Soils, and Sediments using High Throughput Microplate Assays. *Journal of Visualized Experiments*, *80*, 50399. https://doi.org/10.3791/50399

Oram, N. J., Ingrisch, J., Bardgett, R. D., Brennan, F., Dittmann, G., Gleixner, G., Illmer, P., Praeg, N., & Bahn, M. (2023). Drought intensity alters productivity, carbon allocation and plant nitrogen uptake in fast versus slow grassland communities. *Journal of Ecology*, *111*(8), 1681–1699. https://doi.org/10.1111/1365-2745.14136

Sinsabaugh, R. L., & Linkins, A. E. (1988). Exoenzyme activity associated with lotic epilithon. *Freshwater Biology*. https://onlinelibrary.wiley.com/doi/abs/10.1111/j.1365-2427.1988.tb00449.x

Vancov, T., & Keen, B. (2009). Amplification of soil fungal community DNA using the ITS86F and ITS4 primers. *FEMS Microbiology Letters*, *296*(1), 91–96. https://doi.org/10.1111/j.1574-6968.2009.01621.x

Walters, W., Hyde, E. R., Berg-Lyons, D., Ackermann, G., Humphrey, G., Parada, A., Gilbert, J. A., Jansson, J. K., Caporaso, J. G., Fuhrman, J. A., Apprill, A., & Knight, R. (2016). Improved Bacterial 16S rRNA Gene (V4 and V4-5) and Fungal Internal Transcribed Spacer Marker Gene Primers for Microbial Community Surveys. *mSystems*, *1*(1), e00009-15. https://doi.org/10.1128/mSystems.00009-15
